# Supplementary figures and images for: Hepatitis B virus hijacks TSG101 to facilitate egress via multiple vesicle bodies
Source: PLoS Pathog. 2023 May 24;19(5):e1011382. doi: 10.1371/journal.ppat.1011382 (PMC10208485; doi:10.1371/journal.ppat.1011382)

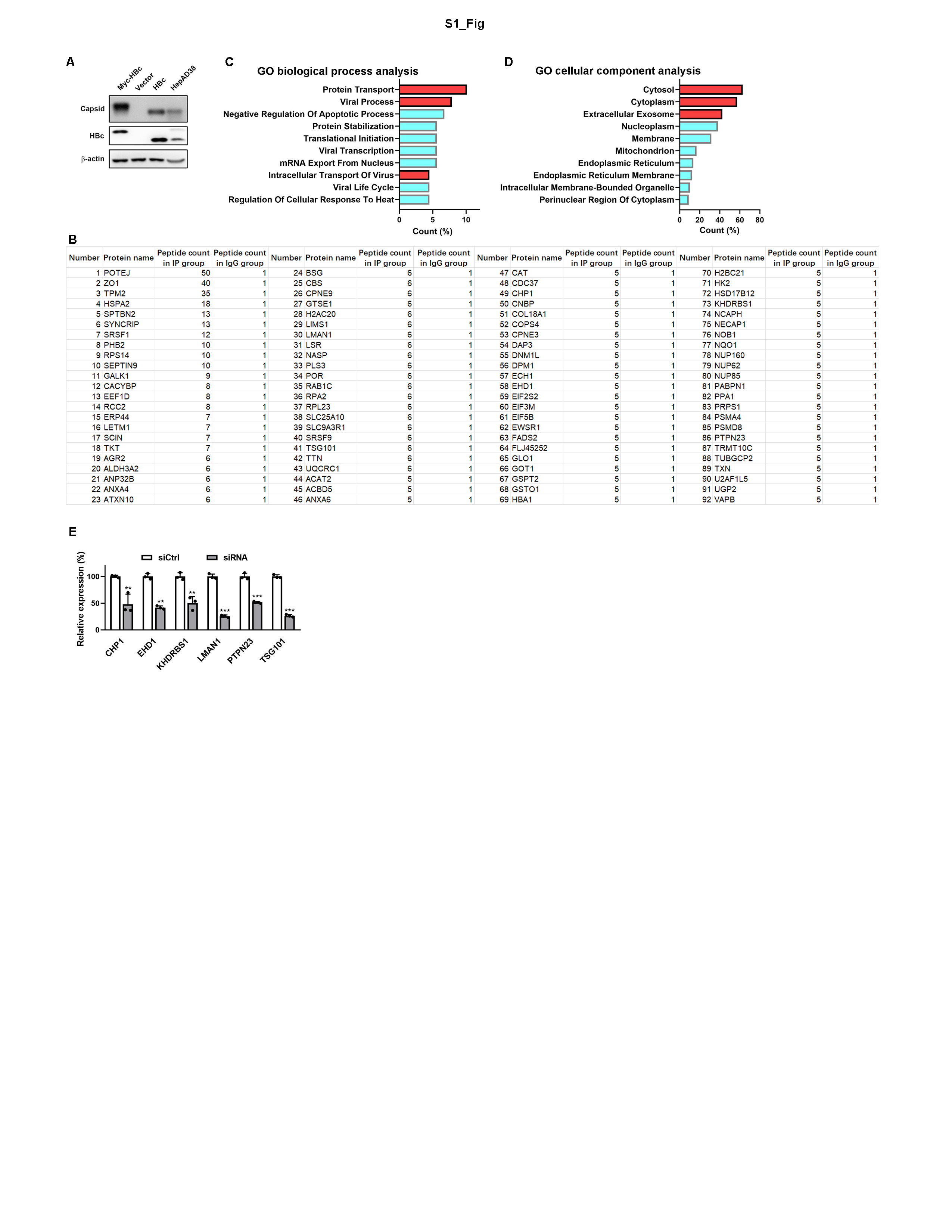

Supplement: S1 Fig — (A) Myc-HBc or HBc expressing plasmid was transfected into Huh7 cells. Intracellular capsids were detected by agarose gel electrophoresis-immunoblot. HepAD38 cells were set as positive control. (B) Myc-HBc was transfected into Huh7 cells. 72 hours after transfection, the cells were suspended with RIPA (weak) lysis buffer. Myc IP experiment was carried out on the cell lysates. IgG IP was set as negative control. Mass spectrometry was performed and identified 92 HBc binding proteins. (C and D) Gene ontology analysis of biological process and cellular component with DAVID bioinformation database for the HBc binding factors. (C) The enrichment of biological processes ranked in top 10 were shown. (D) The enrichment of cellular components ranked in top 10 were shown. (E) HepAD38 cells were transfected with CHP1, EHD1, KHDRBS1, LMAN1, PTPN23, or TSG101 targeted siRNA respectively and maintained in DMEM containing 2% DMSO for 2 days. Levels of the genes mRNA were determined by qPCR assay (% of siRNA). Values show the mean ± SD. **p < 0.01, ***p < 0.001. (TIFF) [file ppat.1011382.s001.tiff]

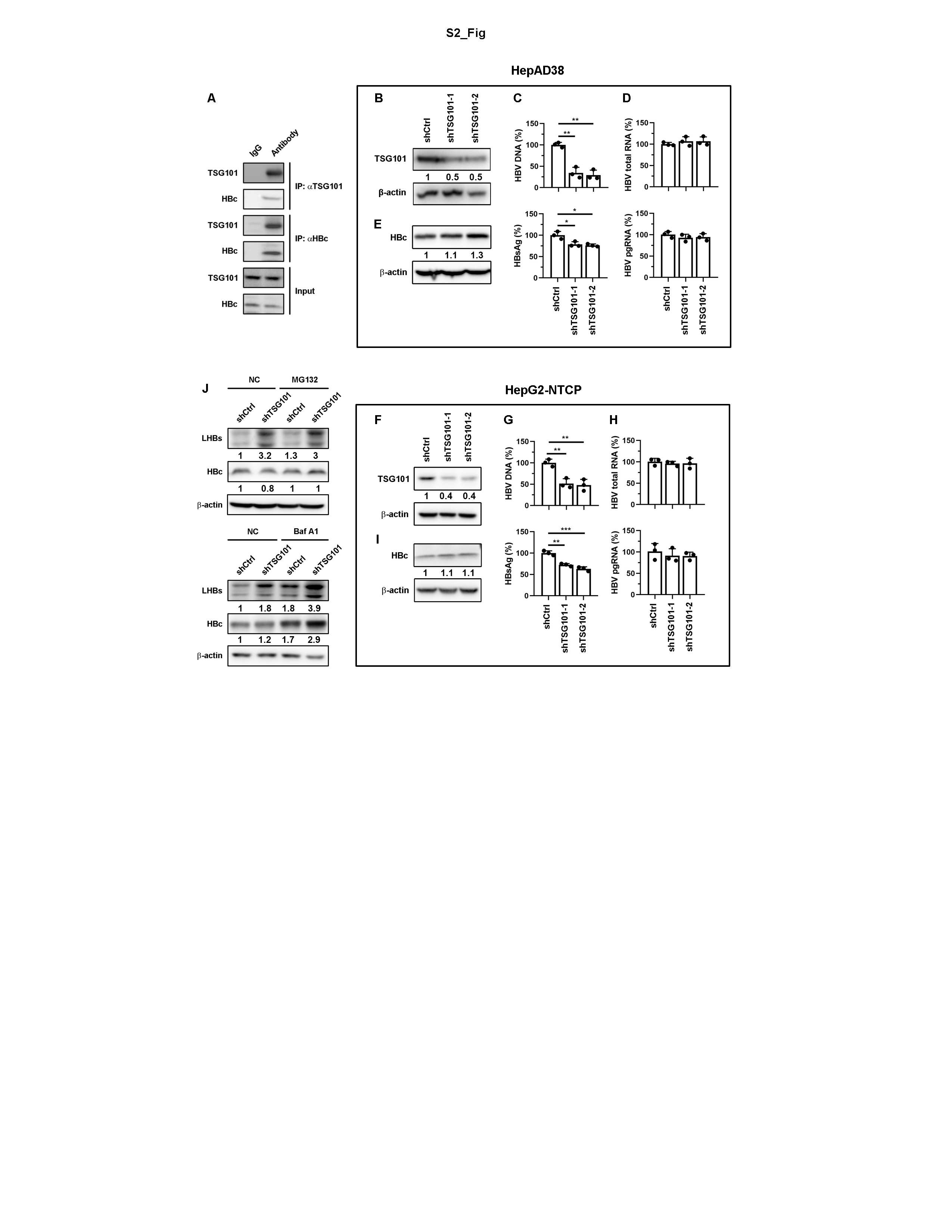

Supplement: S2 Fig — (A) HBc IP and TSG101 IP experiments were conducted on HepAD38 cell lysates. IgG IP was set as a negative control. (B-E) HepAD38 cells with or without stable knockdown of TSG101 were maintained with DMEM supplemented with 2% DMSO for 2 days. (F-I) HepG2-NTCP cells with or without stable knockdown of TSG101 were pretreated with 2.5% DMSO for 2 days following HBV infection at an MOI of 200 and maintained with 2.5% DMSO for 7 days. (B, E, F, and I) Levels of intracellular TSG101 and HBc were determined by WB. (C and G) Levels of HBV DNA and HBsAg in cell culture supernatant were determined by qPCR and ELISA respectively (% of shCtrl). (D and H) Levels of intracellular HBV total RNA and pgRNA were determined by qPCR (% of shCtrl). Values show the mean ± SD. *p < 0.05, **p < 0.01, ***p < 0.001. (J) HepAD38 cells with or without stable knockdown of TSG101 were maintained with DMEM supplemented with 2% DMSO with or without the treatment of 20nM MG132 for 16 hours or 100 nM Bafilomycin A1 for 16 hours. Levels of intracellular L-HBs and HBc were determined by WB. (TIFF) [file ppat.1011382.s002.tiff]

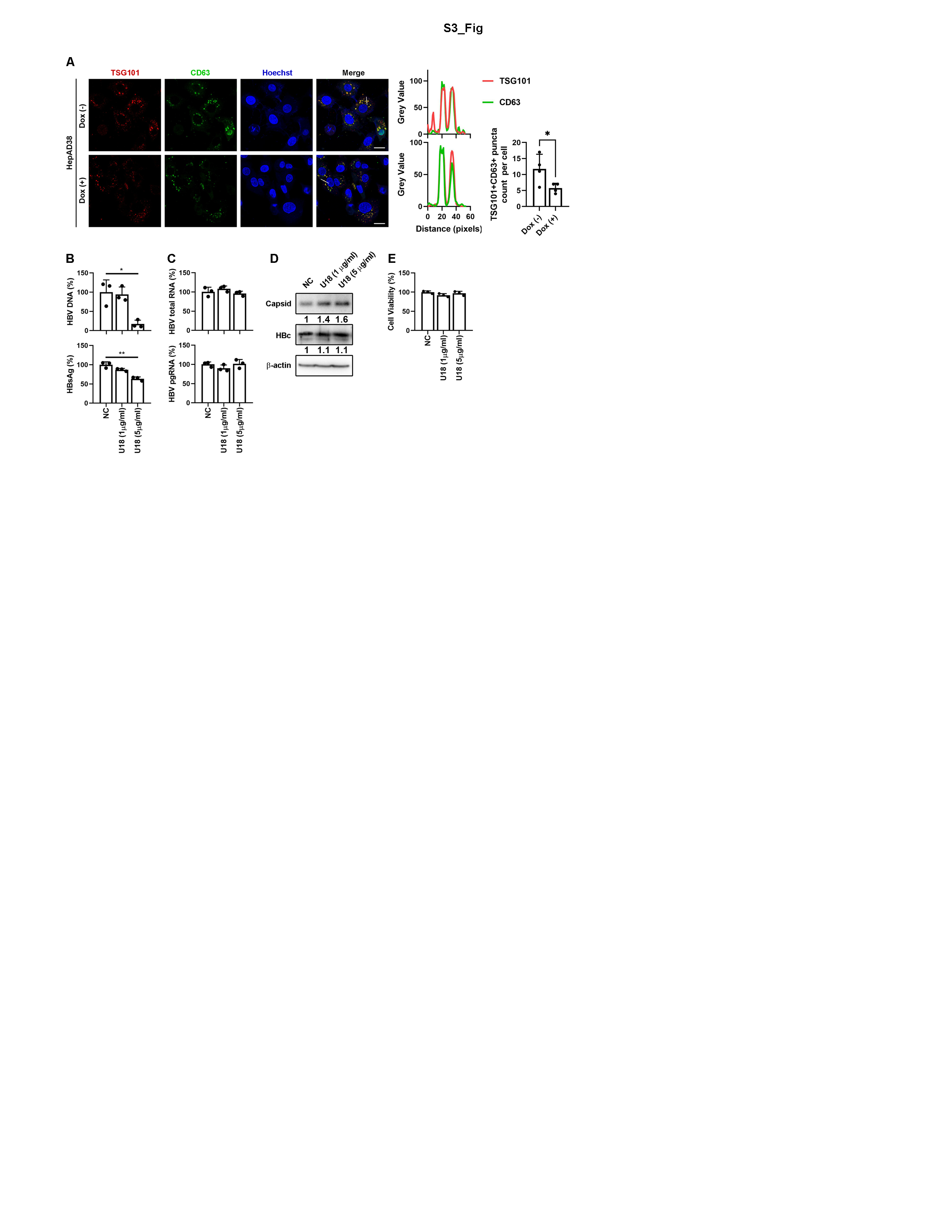

Supplement: S3 Fig — (A) HepAD38 cells were treated with or without doxycycline 1 μg/ml for 10 days. The subcellular distribution of TSG101 and CD63 were detected by immunofluorescence (IF) assay. The fluorescence intensity of TSG101 and CD63 along the indicated line were scanned by software ImageJ. Quantification of TSG101+CD63+ puncta. Four random cells with TSG101+CD63+ puncta were observed. Values show the mean ± SEM, *p < 0.05. (B-E) HepG2-NTCP cells were pretreated with 2.5% DMSO for 2 days following HBV infection at an MOI of 200 and maintained with 2.5% DMSO. The cell was treated with 1 μg/ml or 5 μg/ml U18666A from 3 days post infection. The cell culture supernatants and the cells were harvested at 7 days post infection. (B) Levels of HBV DNA and HBsAg in cell culture supernatant were determined by qPCR and ELISA respectively (% of NC). (C) Levels of intracellular HBV total RNA and pgRNA were determined by qPCR (% of NC). (D) Levels of intracellular HBc were determined by WB. The intracellular HBV capsids were detected by agarose gel electrophoresis-immunoblot assay. (E) Cell viability was evaluated by CCK-8 assay (% of NC). Values show the mean ± SD. *p < 0.05, **p < 0.01. (TIFF) [file ppat.1011382.s003.tiff]

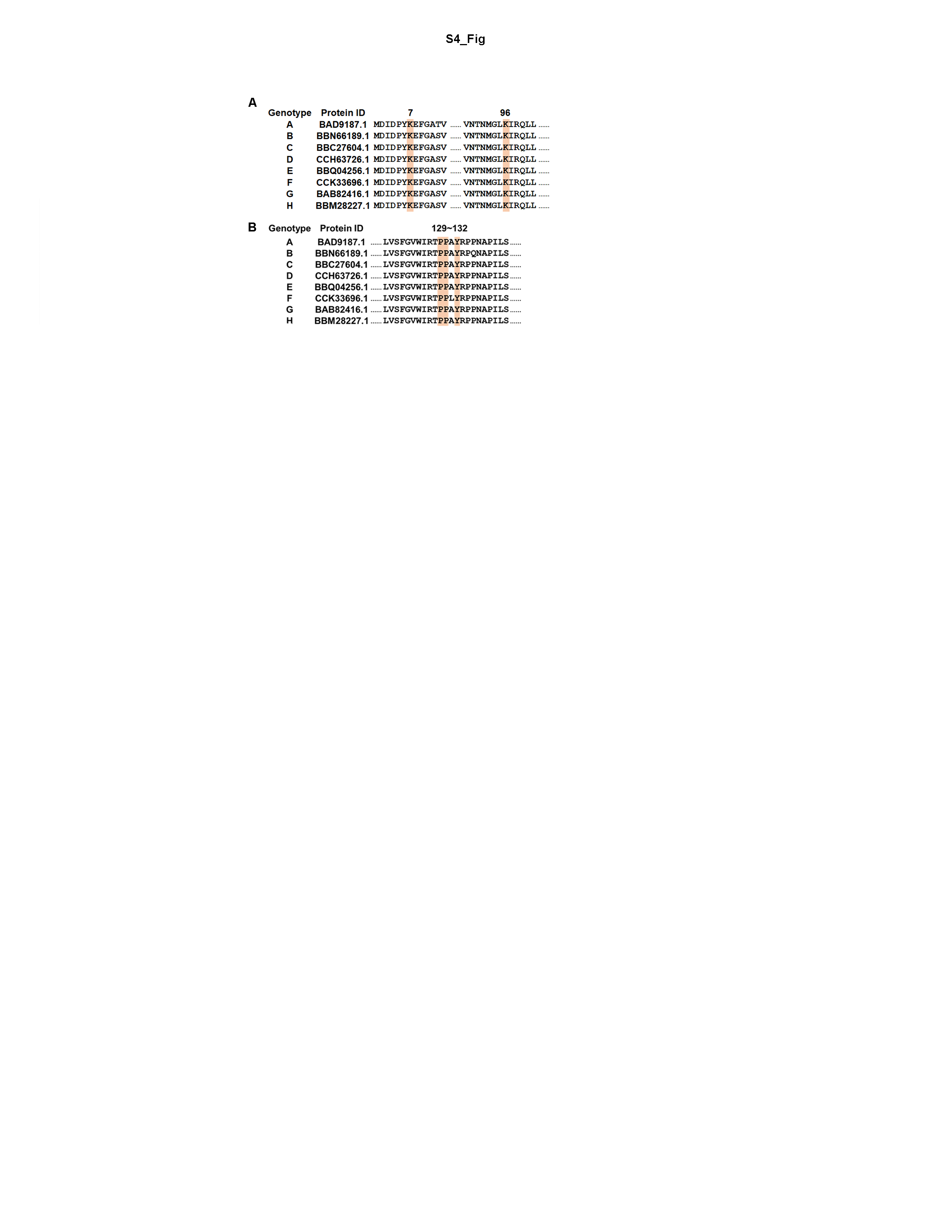

Supplement: S4 Fig — Sequence analysis for lysine sites (A) and PPxY motif (B) of HBc from different genotype of HBV. (TIFF) [file ppat.1011382.s004.tiff]

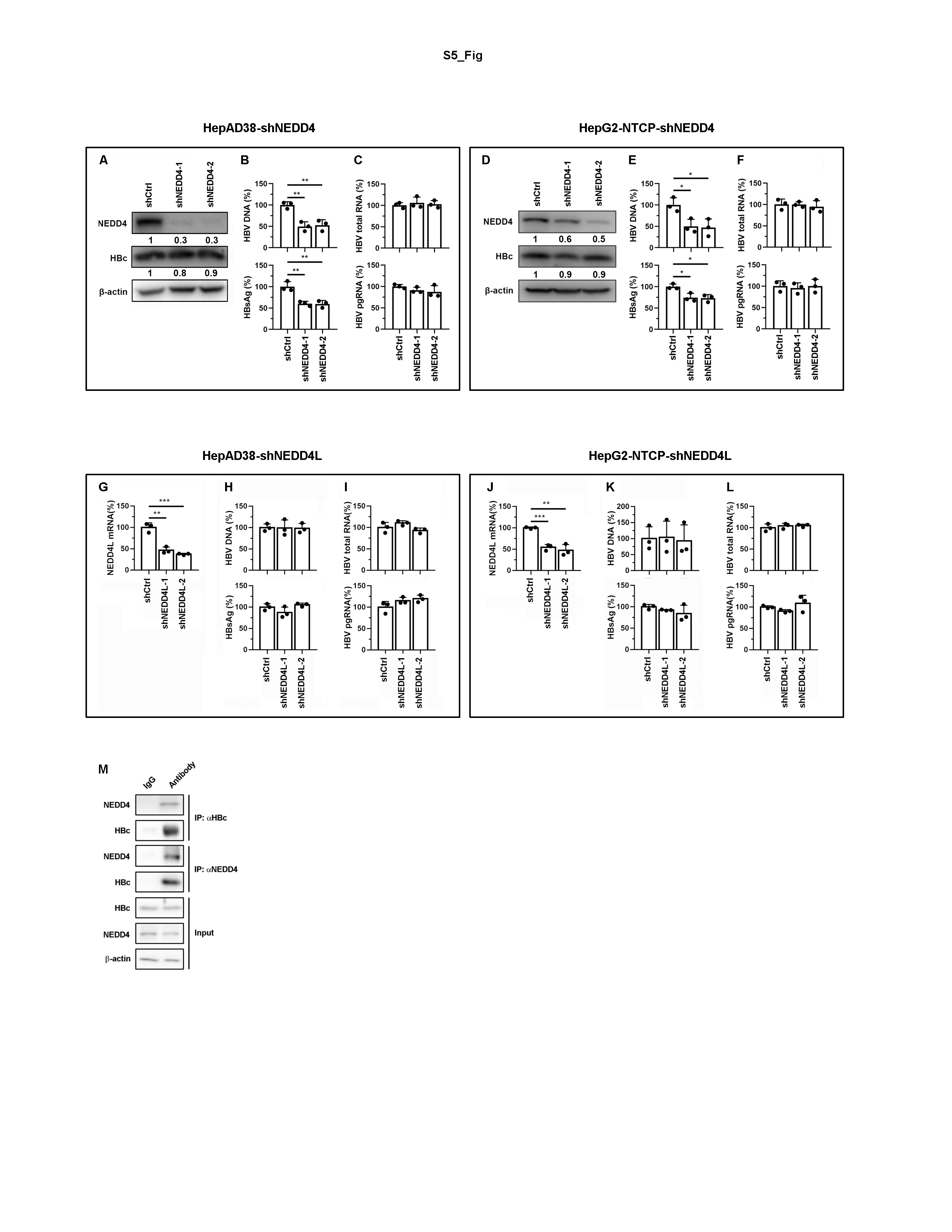

Supplement: S5 Fig — (A-C) HepAD38 cells with or without stable knockdown of NEDD4 were maintained with DMEM supplemented with 2% DMSO for 2 days. (D-F) HepG2-NTCP cells with or without stable knockdown of NEDD4 were pretreated with 2.5% DMSO for 2 days following HBV infection at an MOI of 200 and maintained with 2.5% DMSO for 7 days. (G-I) HepAD38 cells with or without stable knockdown of NEDD4L were maintained with DMEM supplemented with 2% DMSO for 2 days. (J-L) HepG2-NTCP cells infected with lentivirus expressing NEDD4L-targeted shRNAs were pretreated with 2.5% DMSO for 2 days following HBV infection at an MOI of 200 and maintained with 2.5% DMSO for 7 days. (A and D) Levels of NEDD4 and HBc were determined by WB. (B, E, H, and K) Levels of HBV DNA and HBsAg in cell culture supernatant were determined by qPCR and ELISA respectively (% of shCtrl). (C, F, I, and L) Levels of intracellular HBV total RNA and pgRNA were determined by qPCR (% of shCtrl). (G and J) Knockdown efficiency of NEDD4L was confirmed by qPCR (% of shCtrl). Values show the mean ± SD. *p < 0.05, **p < 0.01, ***p < 0.001. (M) HBc IP and NEDD4 IP experiments were conducted on HepAD38 cell lysates. IgG IP was set as negative control. (TIFF) [file ppat.1011382.s005.tiff]
