# Supplementary material for: Hepatitis B virus hijacks TSG101 to facilitate egress via multiple vesicle bodies
Source: PLoS Pathog. 2023 May 24;19(5):e1011382. doi: 10.1371/journal.ppat.1011382 (PMC10208485; doi:10.1371/journal.ppat.1011382)
Supplement: S1 Table — (PDF) [file ppat.1011382.s006.pdf]

S1 Table. Antibodies and chemicals.

Antibody	Source	Identifier
Rabbit anti-TSG101	Proteintech	Cat #: 28283-1-AP
Mouse anti-TSG101	Proteintech	Cat #: 67381-1-Ig
Rabbit anti-NEDD4	Proteintech	Cat #: 21698-1-AP
Rabbit anti-NEDD4	Cell Signaling Technology	Cat #: 52443S
Rabbit anti-CD63	Proteintech	Cat #: 25682-1-AP
Mouse anti-CD63	Proteintech	Cat #: 67605-1-Ig
Rabbit anti-Ubiquitin	Proteintech	Cat #: 10201-2-AP
Rabbit anti-HBc	Gene Technology	Cat #: GB058629
Rabbit anti-HBc	Self-made	N/A
Rabbit anti-HBs	NOVUS Biology	Cat #: NB100-62652
Rabbit anti-β-Actin	Cell Signaling Technology	Cat #: 4970L
Mouse anti-Myc	BIOPRIMACY	Cat #: PMK112M
Mouse anti-Flag	BIOPRIMACY	Cat #: PMK001S
Anti-rabbit IgG, HRP-linked	Cell Signaling Technology	Cat #: 7074
Anti-mouse IgG, HRP-linked	Cell Signaling	Cat #: 7076

	Technology	
Anti-mouse IgG (Alexa Fluor 568)	Invitrogen,	Cat #: A-11031
Anti-rabbit IgG (Alexa Fluor 488)	Invitrogen,	Cat #: A-11034
Rabbit IgG	Proteintech	Cat #: B900610
Mouse IgG	Proteintech	Cat #: B900620

**Chemical**	**Source**	**Identifier**
U18666A	MedChemExpress	Cat #: HY-107433
PEI MAX 40K	Polysciences	Cat #: 24765-1
Lipofectamine RNAiMAX	Invitrogen	Cat #: 13778075
Penicillin and streptomycin sulfate	Gibco	Cat #: 15140-122
Dimethyl sulfoxide	Sigma-Aldrich	Cat #: D5879
Polybrene	Solarbio	Cat #: H8761
Skim milk	Difco	Cat #: 232100
Kanamycin	Sigma-Aldrich	Cat #: E004000
Ampicillin	Sigma-Aldrich	Cat #: A9518
MG-132	MedChemExpress	HY-13259
Bafilomycin A1	MedChemExpress	HY-100558
