# Supplementary material for: Hepatitis B virus hijacks TSG101 to facilitate egress via multiple vesicle bodies
Source: PLoS Pathog. 2023 May 24;19(5):e1011382. doi: 10.1371/journal.ppat.1011382 (PMC10208485; doi:10.1371/journal.ppat.1011382)
Supplement: S2 Table — (PDF) [file ppat.1011382.s007.pdf]

**S2 Table. Oligonucleotides**

| Oligonucleotide | Sequence |
|---|---|
| si*LMAN1* | 5’-GCACATAGTAAAGAGGGACAT-3’ |
| si*KHDRBS1* | 5’-GTACCGGATATGATGGATGAT-3’ |
| si*EHD1* | 5’-CGCTTTCCTCAACAGGTTCAT-3’ |
| si*PTPN23* | 5’-CCGCCAGATCCTTACGCTCAA-3’ |
| si*CHP1* | 5’-CGCATGATGGTCGGAGTAAAT-3’ |
| si*TSG101*-1 | 5’-GCCTTATAGAGGTAATACATA-3’ |
| si*TSG101*-2 | 5’-TGGAGGTTGAGCTCTTCTTAA-3’ |
| si*NEDD4*-1 | 5’-CGGTTGGAGAATGTAGCAATA-3’ |
| si*NEDD4*-2 | 5’-AGTGCTACTCGCAGCTATTTA-3’ |
| sh*TSG101*-1 | 5’ACTGGACACATACCCATATAACTCGAG TTATATGGGTATGTGTCCAGTTTTTTG3’ |
| sh*TSG101*-2 | 5’GCCTTATAGAGGTAATACATACTCGAG TATGTATTACCTCTATAAGGCTTTTTG3’ |
| sh*Tsg101* | 5’GCTATTGAAGACACTATCTTTCTCGAGA AAGATAGTGTCTTCAATAGC3’ |
| sh*NEDD4*-1 | 5’GCCTTTCTCTTGCCTGCATATCTCGAGA TATGCAGGCAAGAGAAAGGCTTTTTG3’ |
| sh*NEDD4*-2 | 5’GCCTTTCTCTTGCCTGCATATCTCGAGA TATGCAGGCAAGAGAAAGGCTTTTTG3’ |
| sh*NEDD4L*-1 | 5’GGATGAGAATAGAGAACTTGCCTCGAG GCAAGTTCTCTATTCTCATCCTTTTTG3’ |
| sh*NEDD4L*-2 | 5’GGAACTAAGCAGAAGGCTTCACTCGAG |

| | |
|---|---|
| | TGAAGCCTTCTGCTTAGTTCCTTTTTG3’ |
| *CHP1* forward primer | 5’-CCTTGCTGTGCTACAACCG-3’ |
| *CHP1* reverse primer | 5’-CGGAACACCTGGGTGGTATG-3’ |
| *EHD1* forward primer | 5’-GACAACAAGCCTATGGTGCTC-3’ |
| *EHD1* reverse primer | 5’-AAGTCCTGCTCGATCAGGTGT-3’ |
| *KHDRBS1* forward primer | 5’-GGAGCCAGAGAACAAGTACCT-3’ |
| *KHDRBS1* reverse primer | 5’-CATGGCGTGAGTGAAGGAC-3’ |
| *LMAN1* forward primer | 5’-AGTTGAGGTGACATTTCGAGTG-3’ |
| *LMAN1* reverse primer | 5’-AGCTGATCCAAACACAGGGC-3’ |
| *PTPN23* forward primer | 5’-ATGCCCATGATCTGGCTGG-3’ |
| *PTPN23* reverse primer | 5’-TACTTGCGGAGGACACTACAG-3’ |
| *TSG101* forward primer | 5’-CTTGGGAGAAGCCTTGAGAA-3’ |
| *TSG101* reverse primer | 5’-TGTTTACGGGACAGAAGACGTA-3’ |
| *NEDD4* forward primer | 5’- TGCCAGACTCACCATTTTTG-3’ |
| *NEDD4* reverse primer | 5’- GCCTCTTCTGCTGGAATGAT-3’ |
| *NEDD4L* forward primer | 5’- GGTGGTGAGGAACCAACG-3’ |
| *NEDD4L* reverse primer | 5’- GCGCCCCTTAGCATCTTT-3’ |
| HBV pgRNA forward primer | 5’ - CTGGGTGGGTGTTAATTTGG - 3’ |
| HBV pgRNA reverse primer | 5’ - TAAGCTGGAGGAGTGCGAAT - 3’ |
| HBV total RNA forward primer | 5’ - CCGTCTGTGCCTTCTCATCTGC - 3’ |
| HBV total RNA reverse primer | 5’ - ACCAATTTATGCCTACAGCCTCC - 3’ |
| *β-actin* forward primer | 5’ - ATCGTGCGTGACATTAAGGAG - 3’ |
| *β-actin* reverse primer | 5’ - GGAAGGAAGGCTGGAAGAGT - 3’ |
