# Supplementary material for: Hepatitis B virus hijacks TSG101 to facilitate egress via multiple vesicle bodies
Source: PLoS Pathog. 2023 May 24;19(5):e1011382. doi: 10.1371/journal.ppat.1011382 (PMC10208485; doi:10.1371/journal.ppat.1011382)
Supplement: S3 Table — (PDF) [file ppat.1011382.s008.pdf]

S3 Table. Commercial Kits.

Commercial kit	Manufacturer	Identifier
ELISA HBV Test Kit (HBsAg)	KHB	
Cell Counting Kit-8	Beyotime	Cat #: C0039
ReverTra Ace qPCR RT Master Mix with gDNA Remover	TOYOBO	Cat #: FSQ-301
HBV DNA Diagnostic Kit	Sansure	
EndoFree Plasmid Midi Kit	CWBIO	Cat #: CW2105S
Ultrapure RNA Kit	CWBIO	Cat #: CW0581
FastPure Plasmid Mini Kit	Vazyme	Cat #: DC201-01
FastPure Gel DNA Extraction Mini Kit	Vazyme	Cat #: DC301-01
TIANamp Genomic DNA Kit	TIANGEN BIOTECH	Cat #: DP304-03
Enzo Ubiquitinylation kit	Enzo life science	Cat #: BML-UW9920
BeyoGol His-tag Purification Resin	Beyotime	Cat #: P2218
BeyoGol GST-tag Purification Resin	Beyotime	Cat #: P2253
