# Supplementary material for: Hepatitis B virus hijacks TSG101 to facilitate egress via multiple vesicle bodies
Source: PLoS Pathog. 2023 May 24;19(5):e1011382. doi: 10.1371/journal.ppat.1011382 (PMC10208485; doi:10.1371/journal.ppat.1011382)

Fig 2A

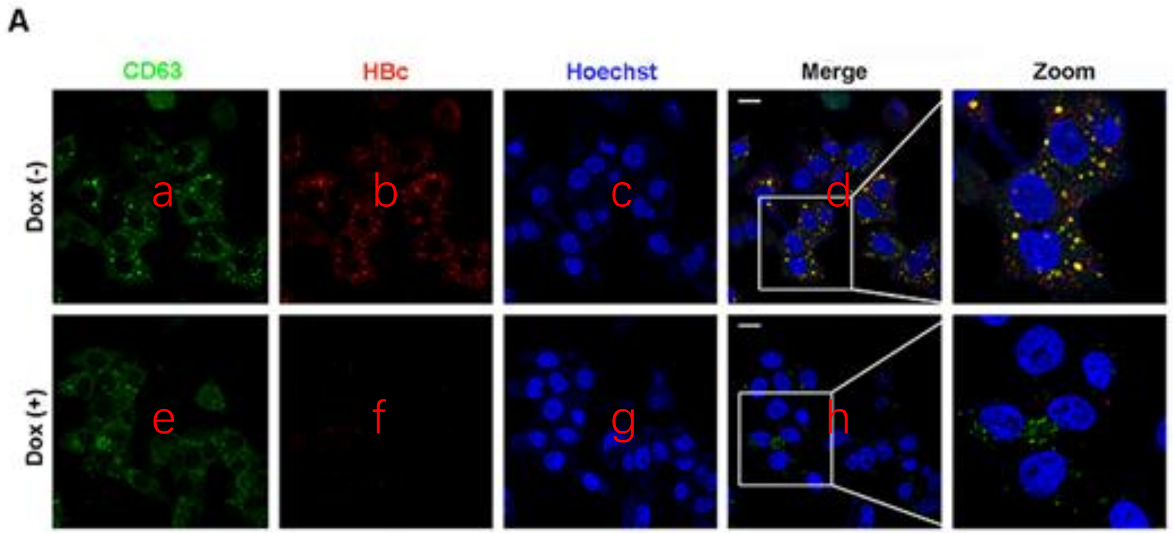

a

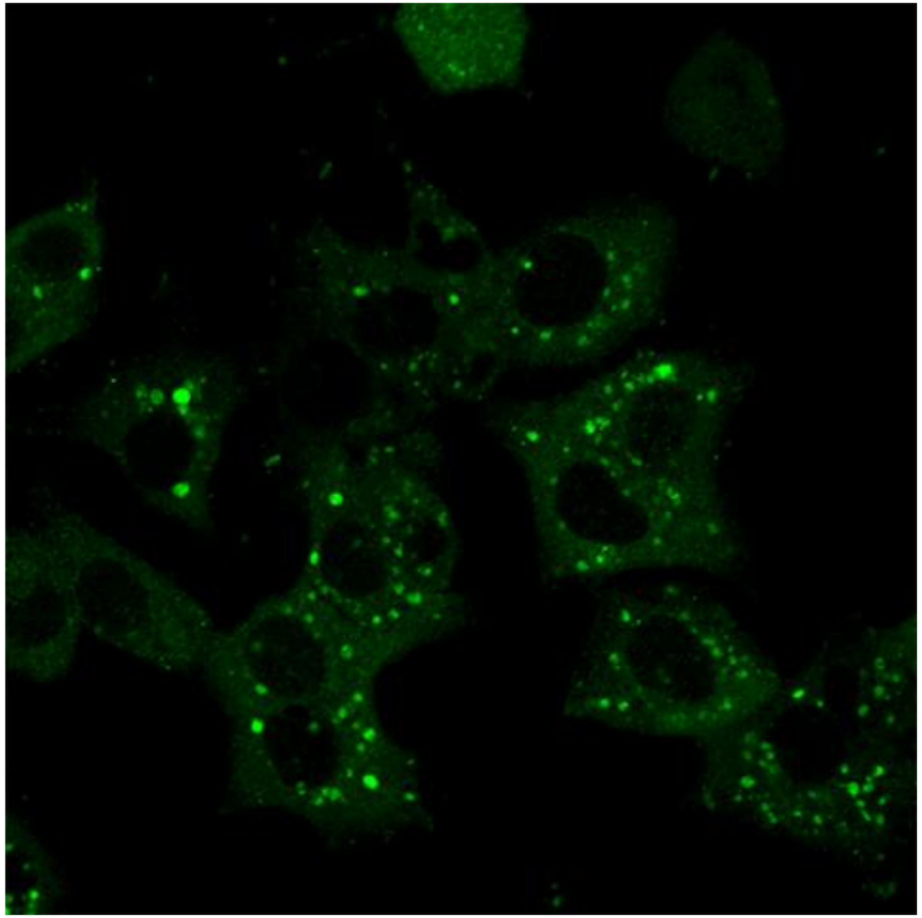

b

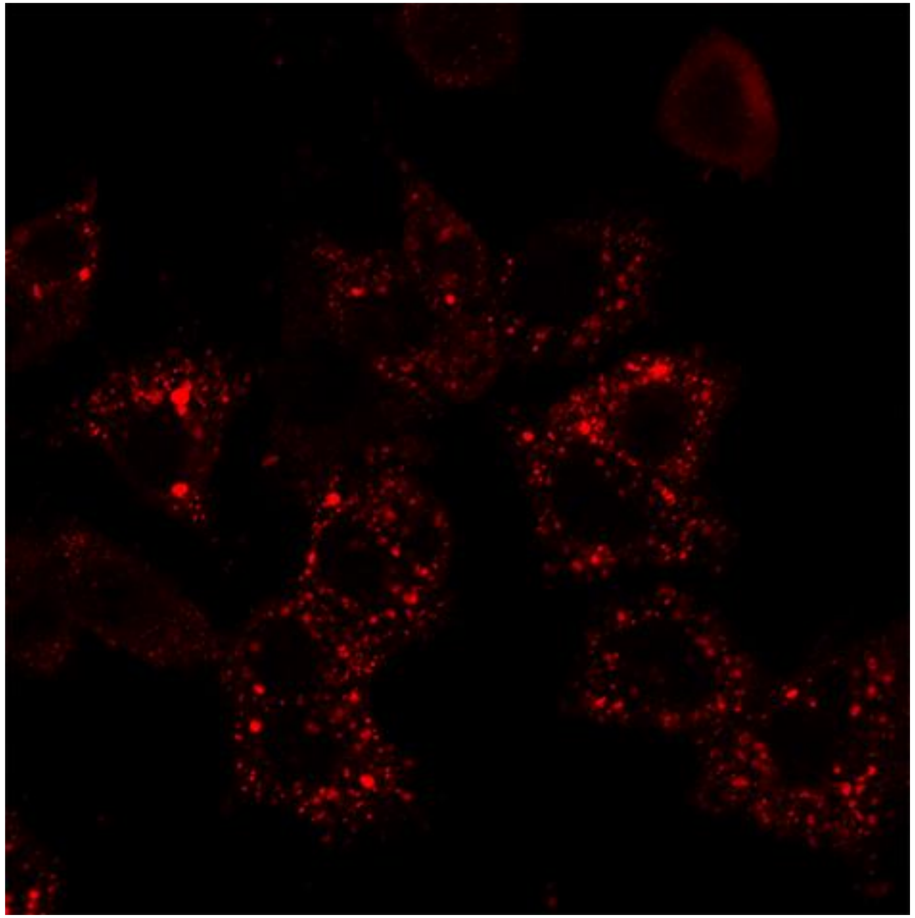

C

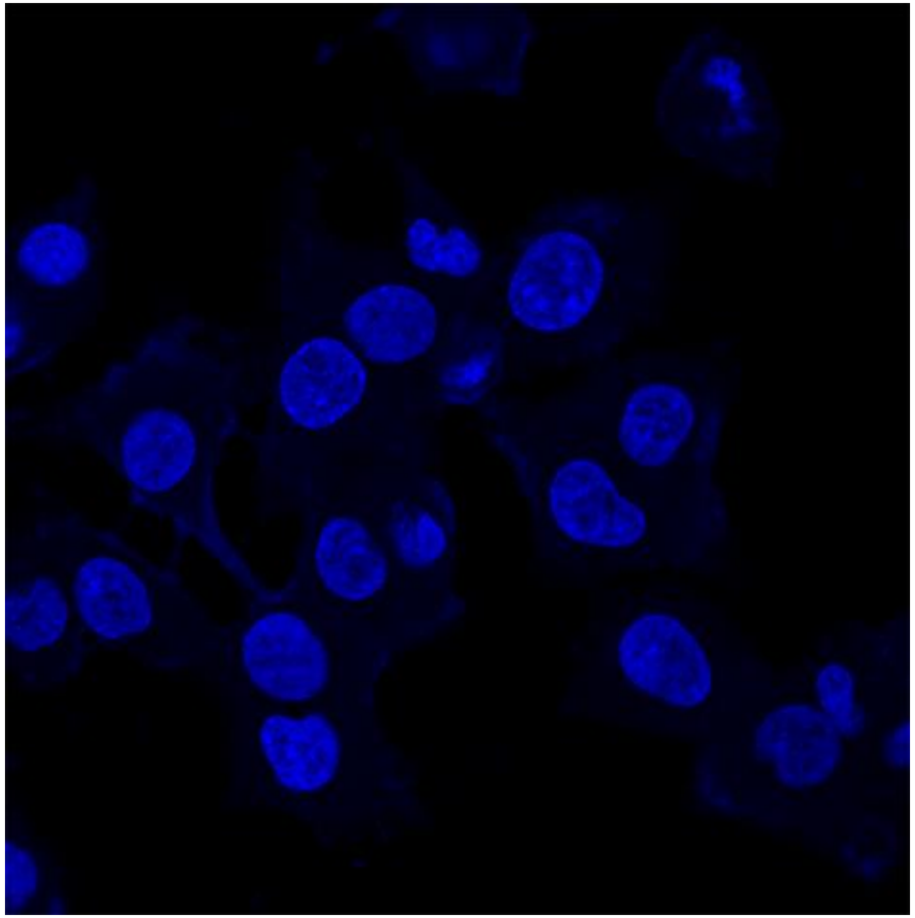

d

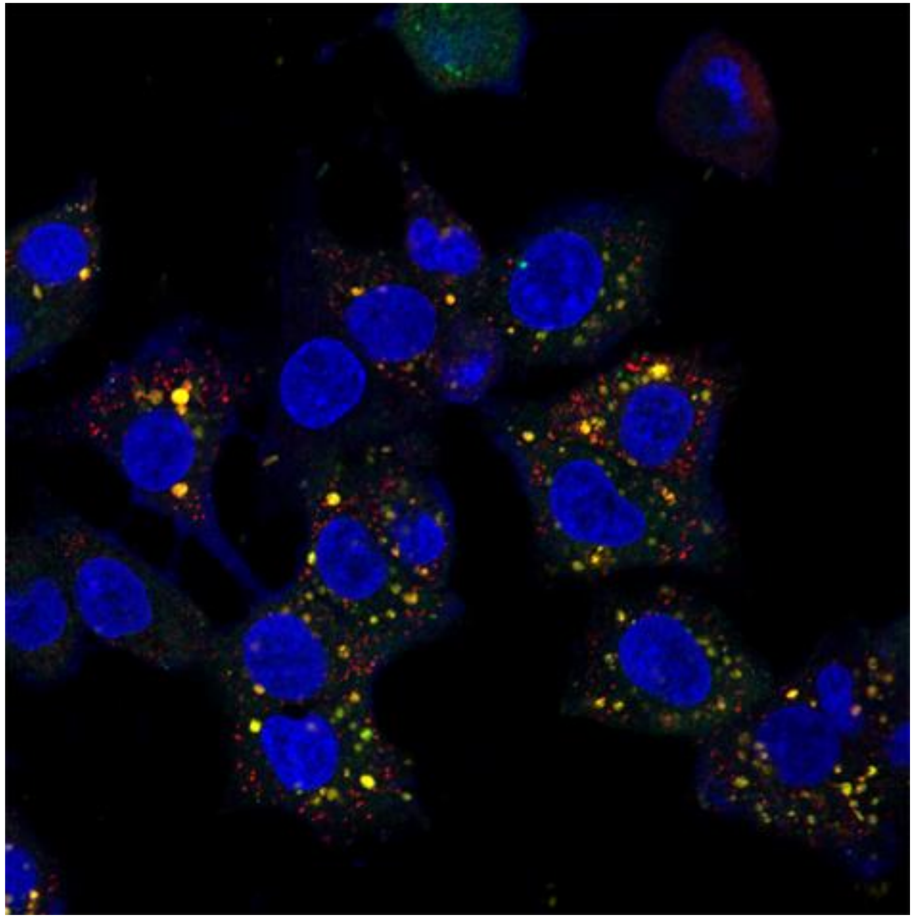

e

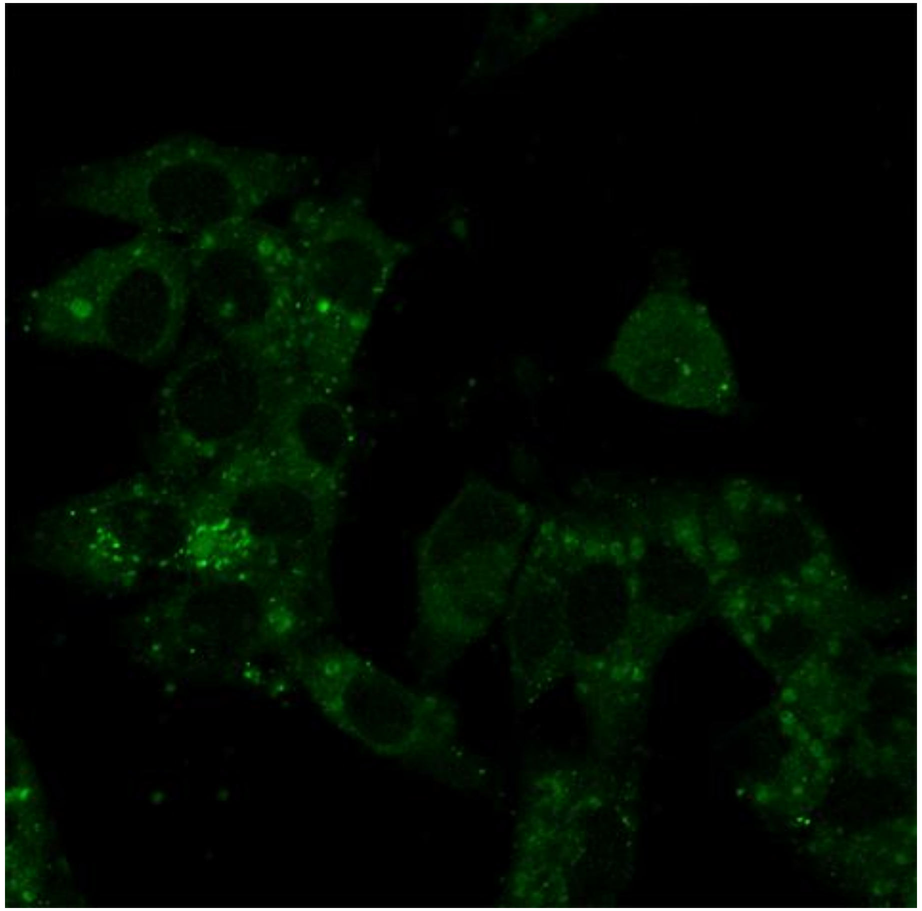

f

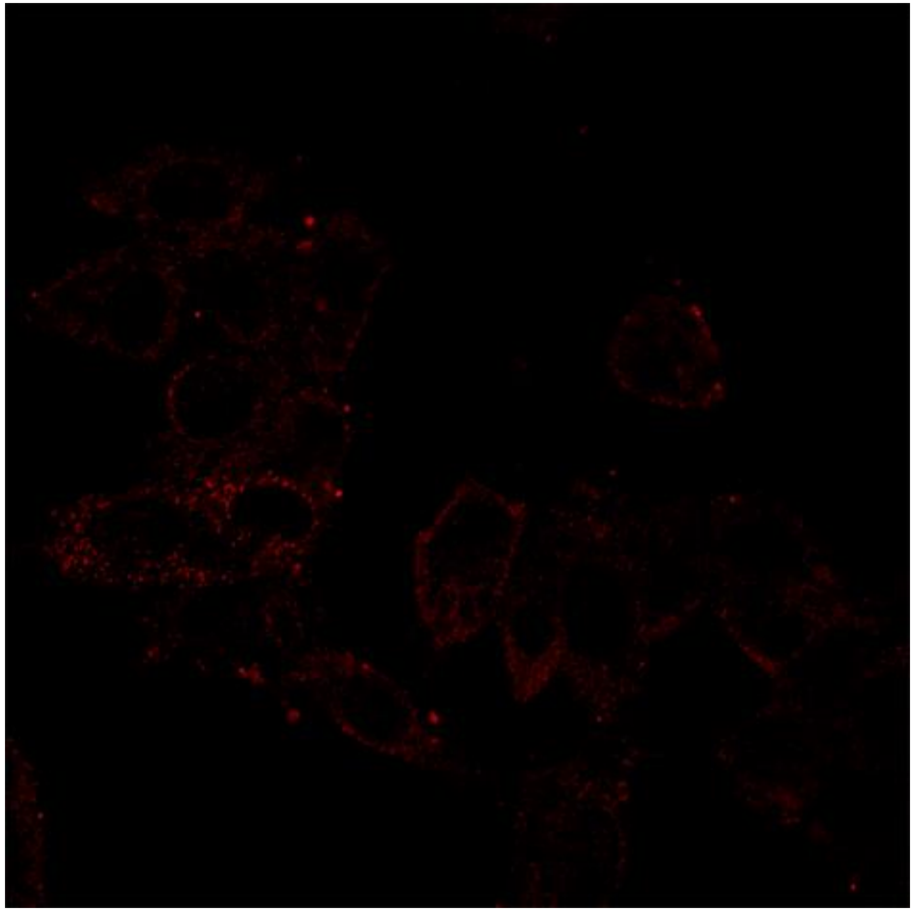

g

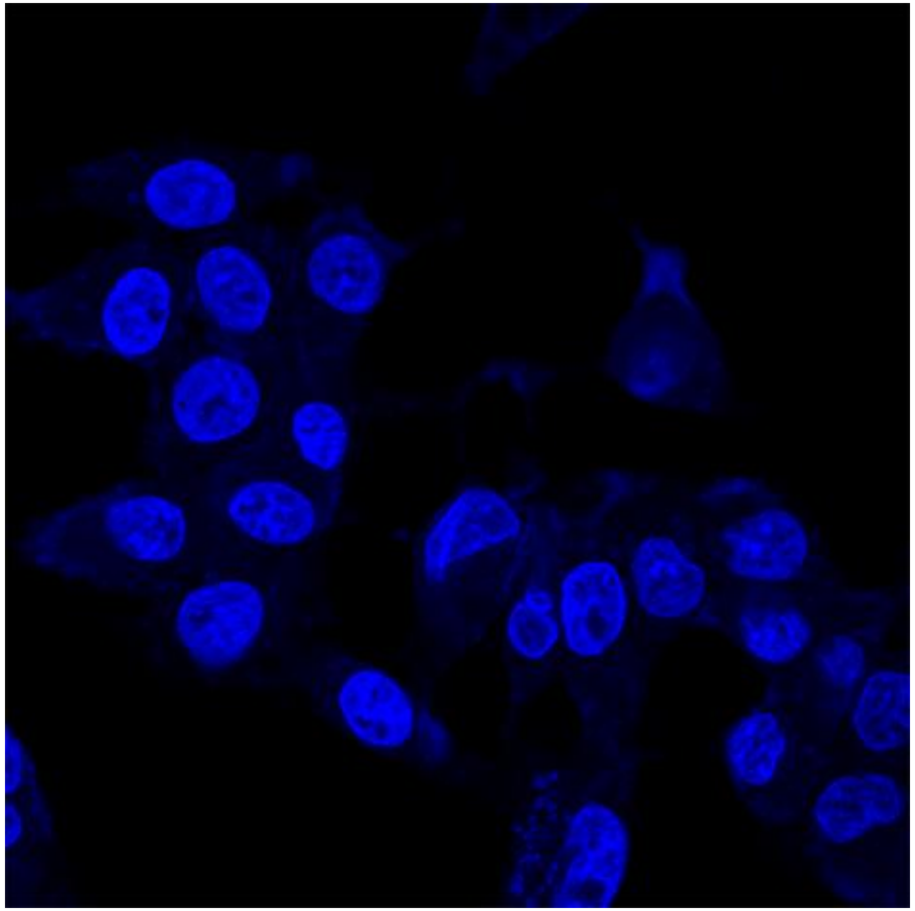

h

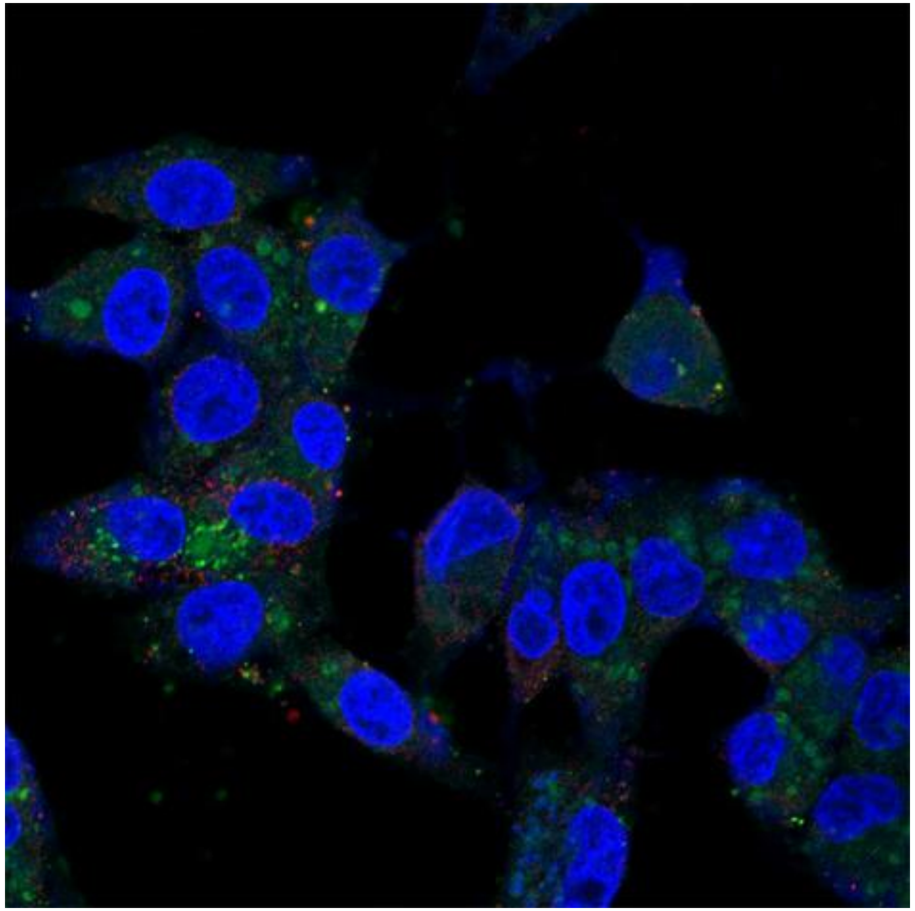

Fig 2B

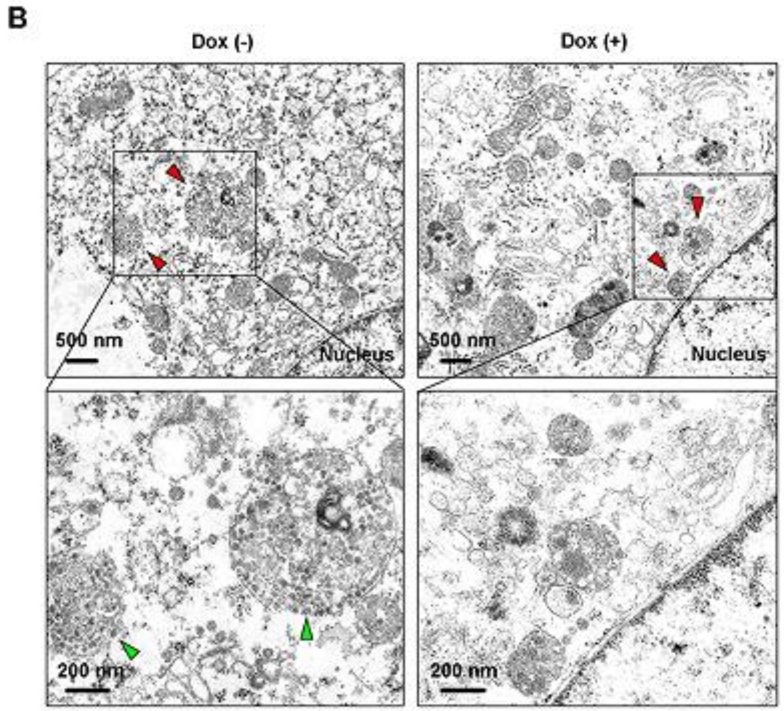

HepAD38
Dox (-)

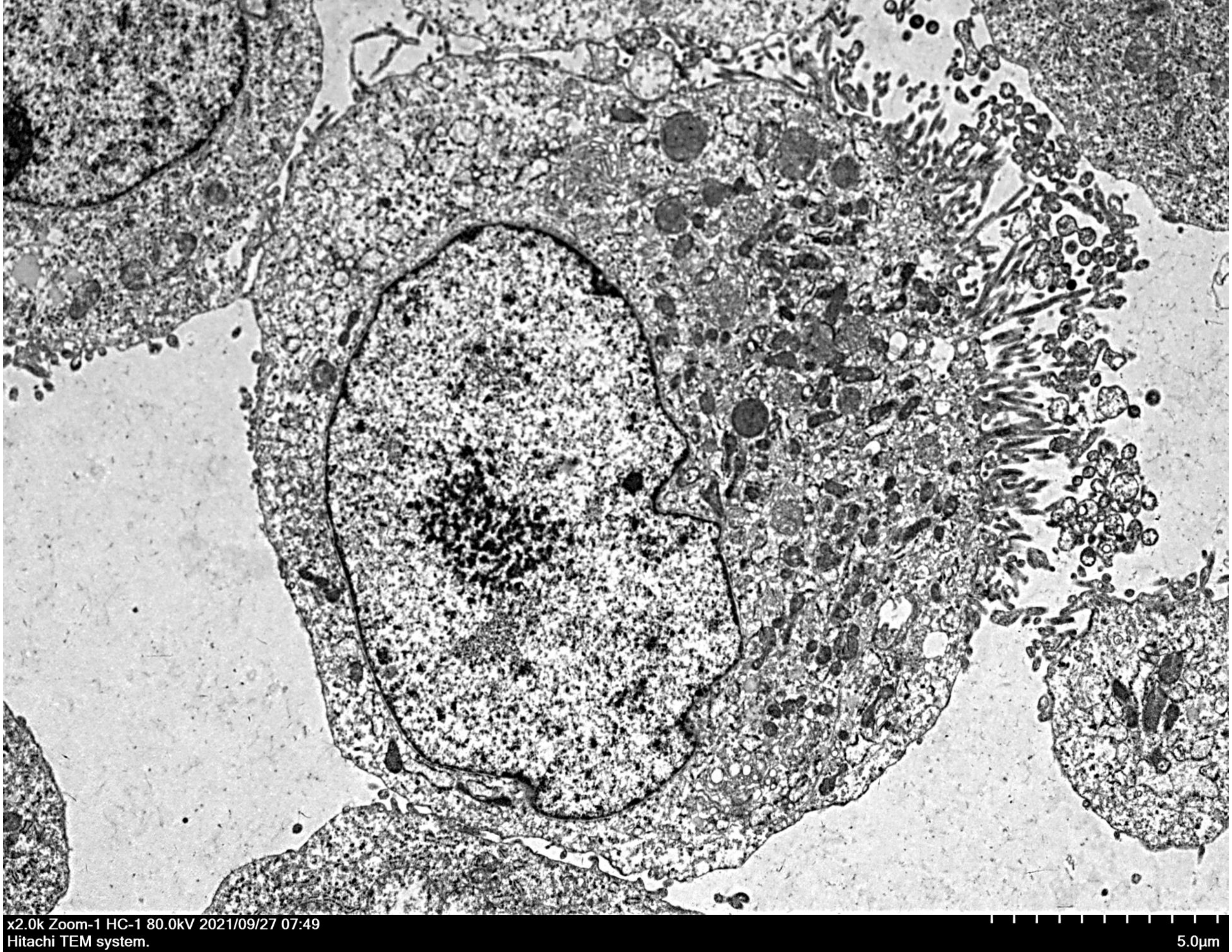

HepAD38
Dox (-)

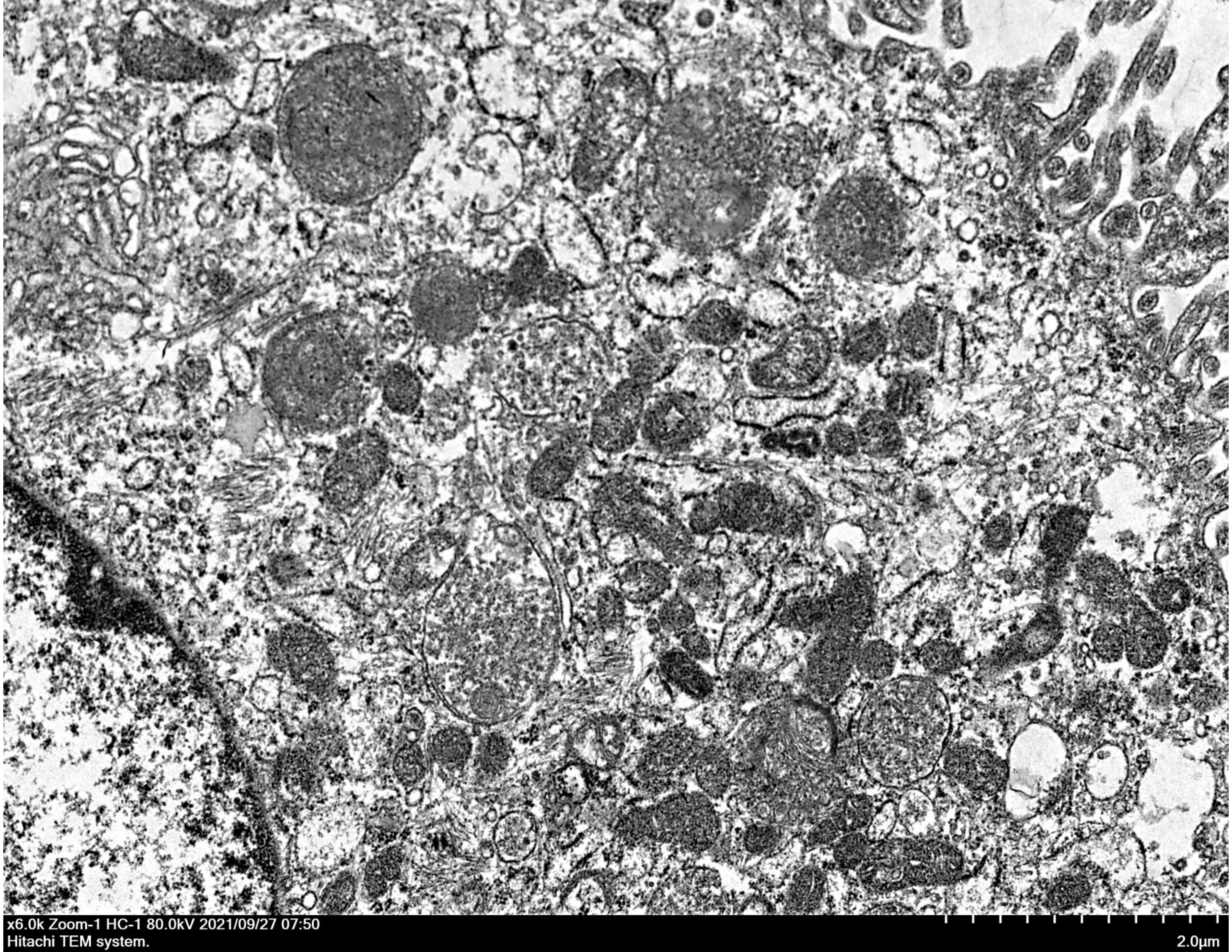

HepAD38
Dox (-)

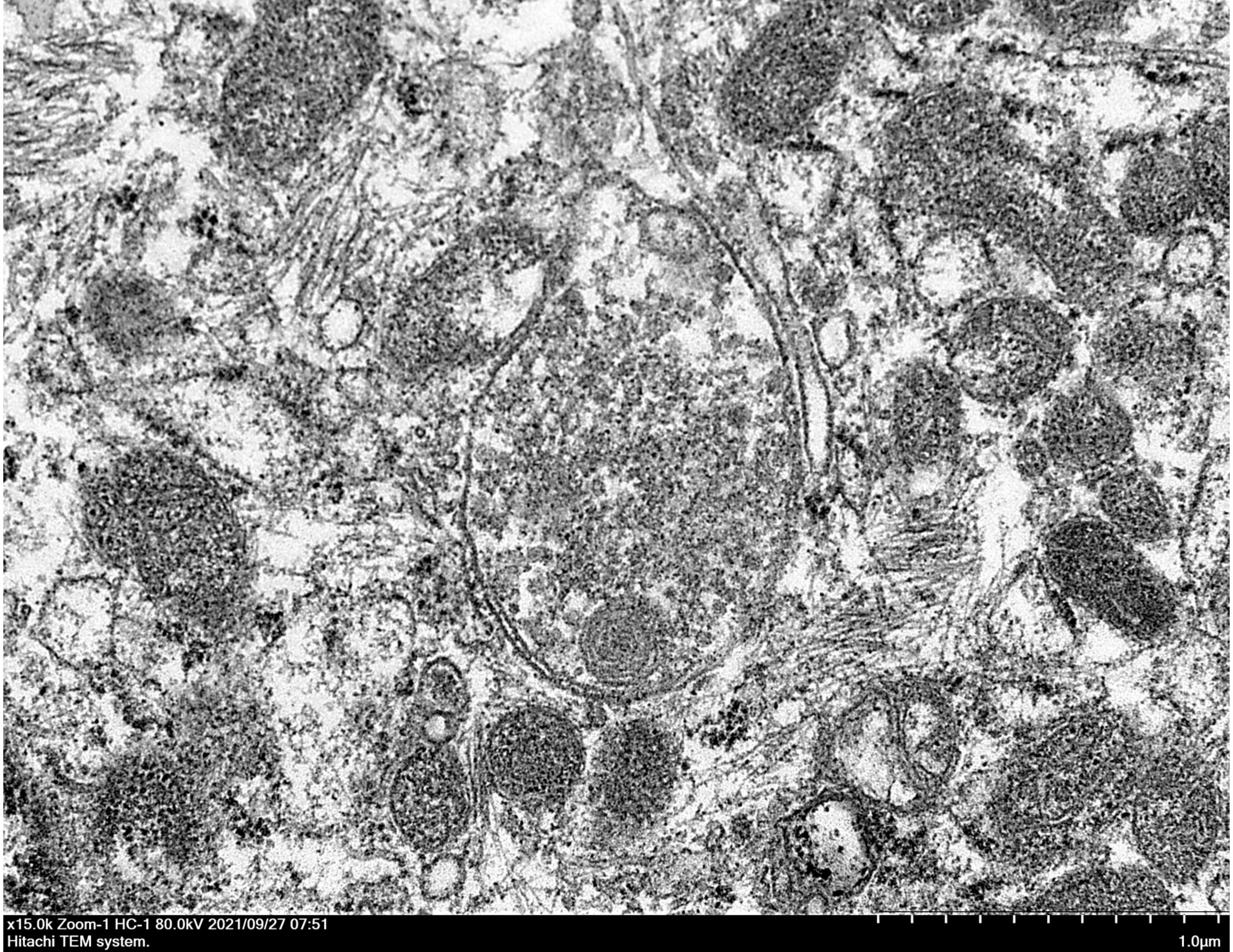
x15.0k Zoom-1 HC-1 80.0kV 2021/09/27 07:51
Hitachi TEM system.
1.0μm

HepAD38
Dox (-)

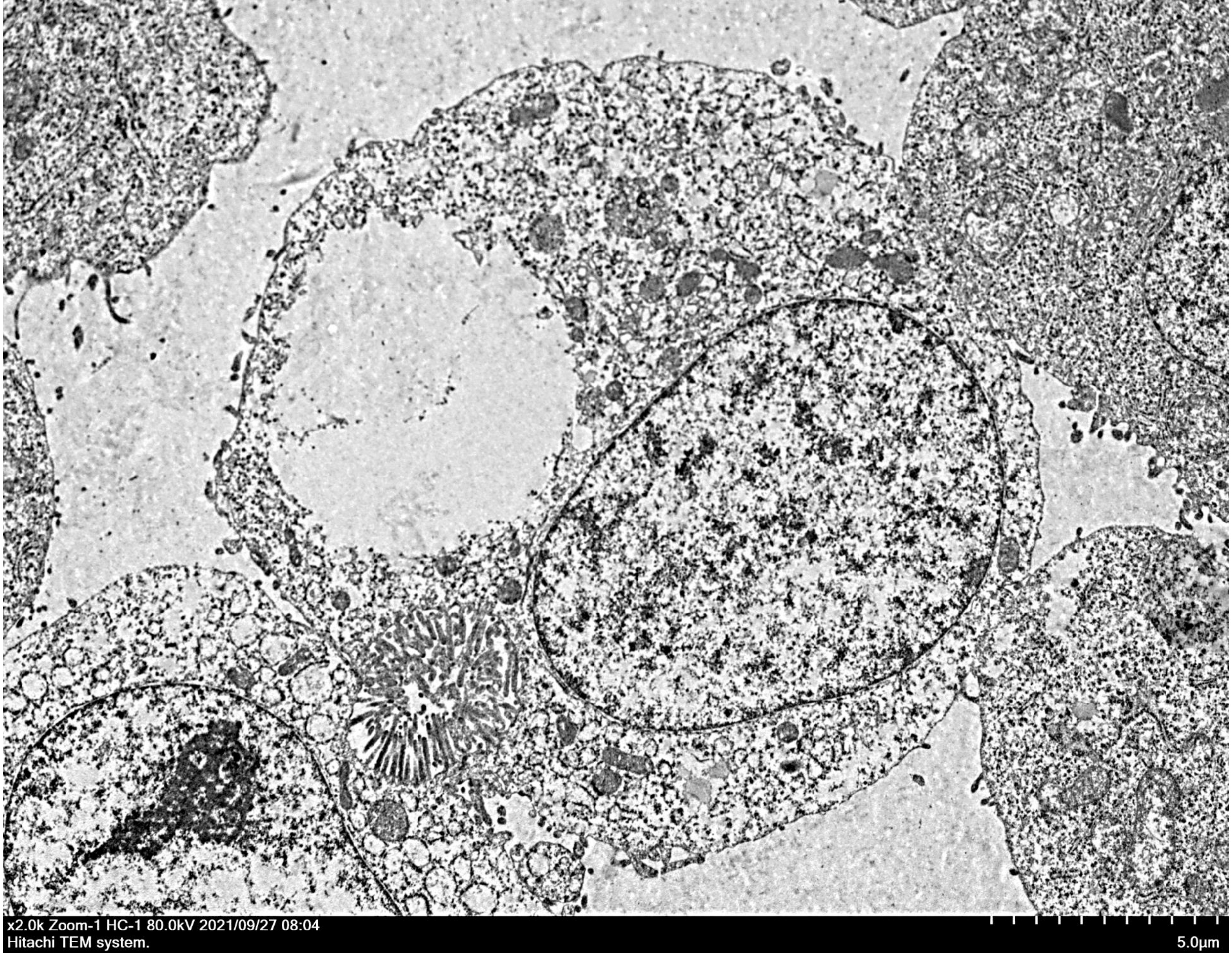

HepAD38
Dox (-)

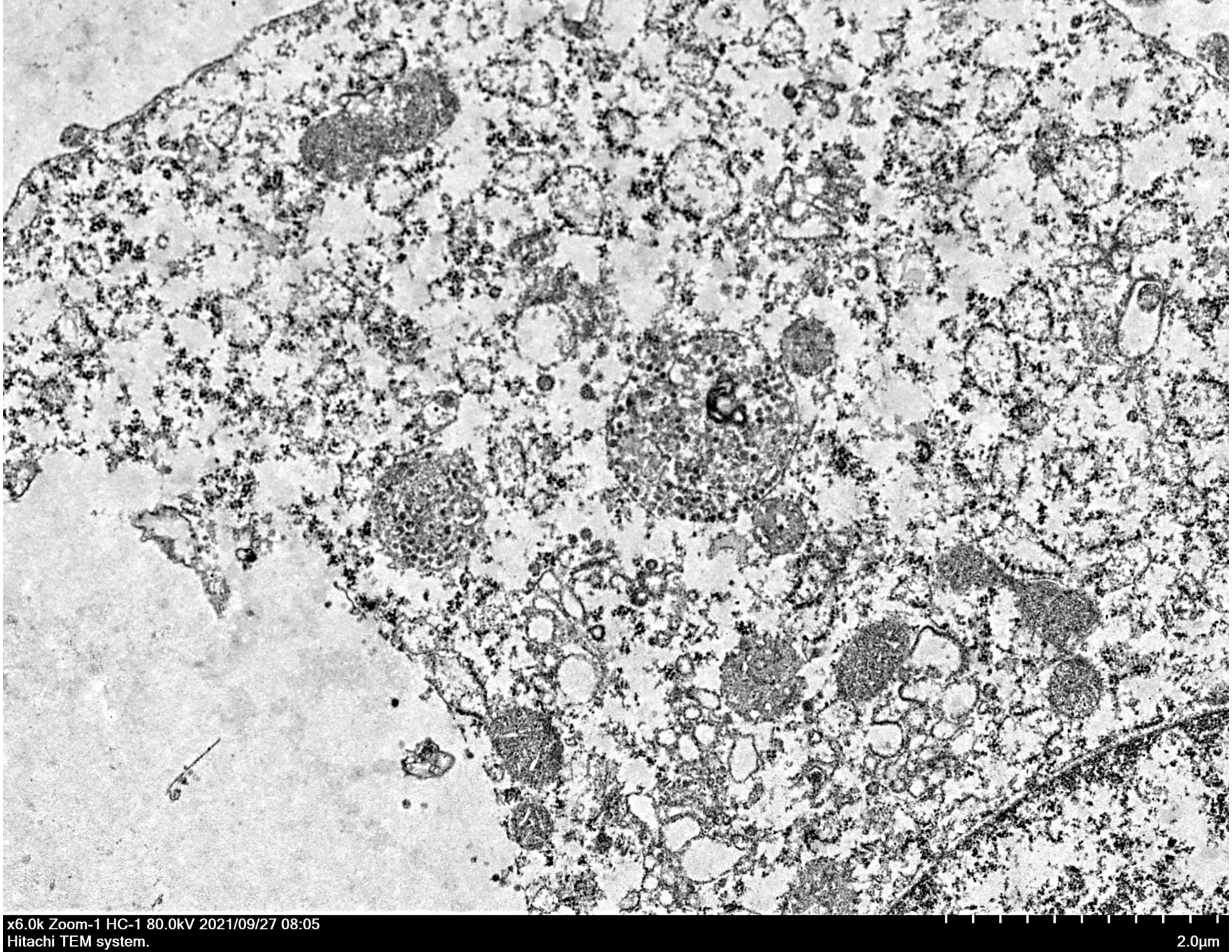

HepAD38
Dox (-)

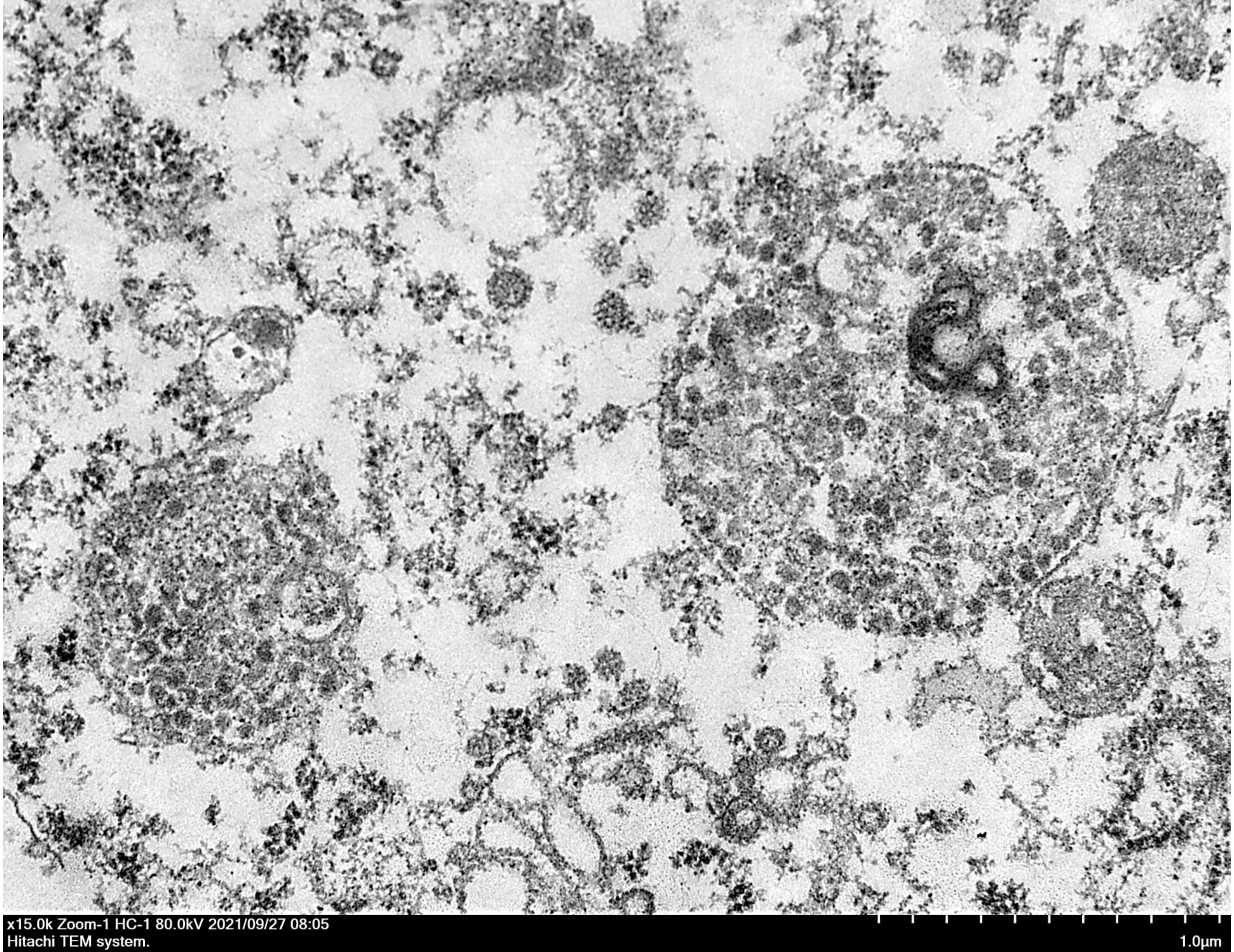
x15.0k Zoom-1 HC-1 80.0kV 2021/09/27 08:05
Hitachi TEM system.
1.0μm

HepAD38
Dox (-)

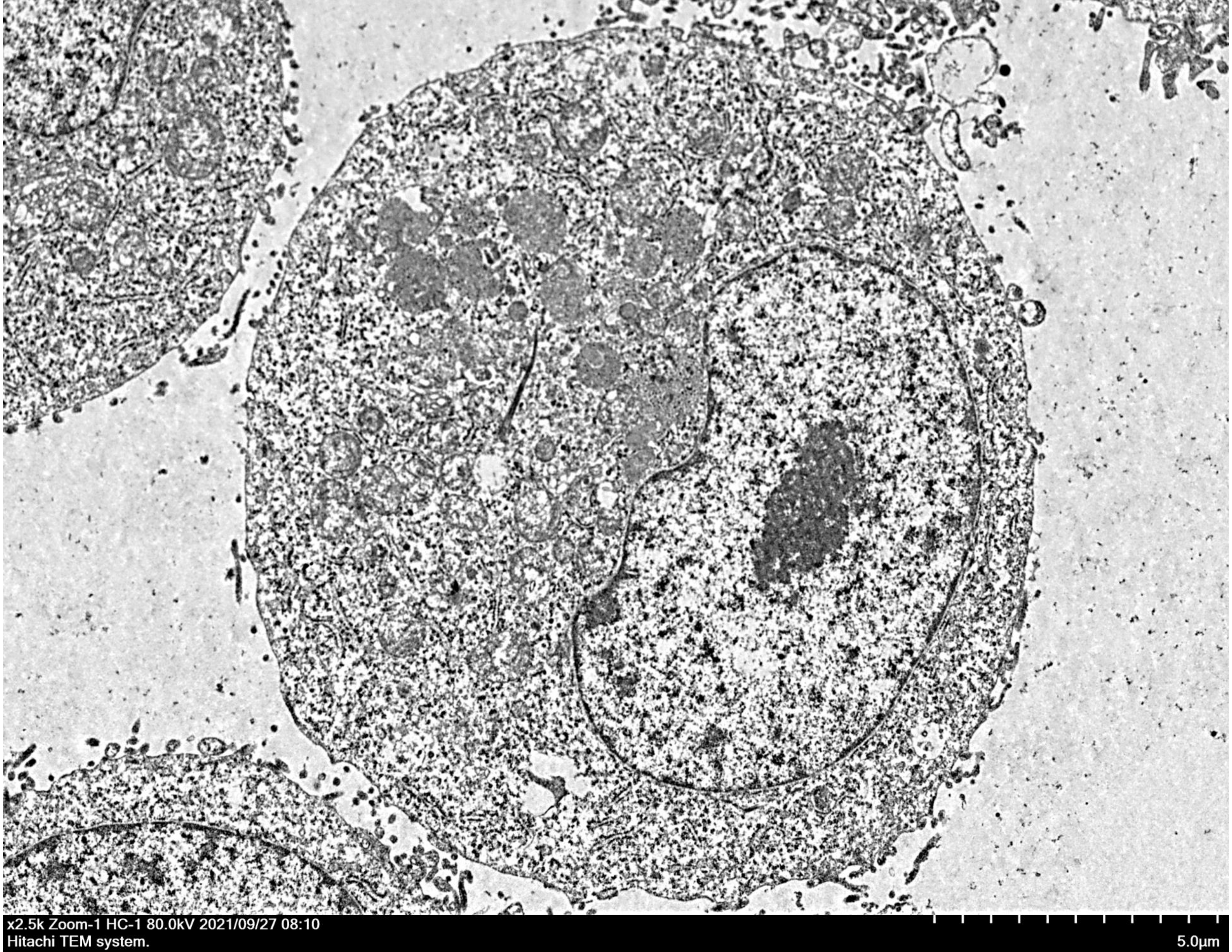

HepAD38
Dox (-)

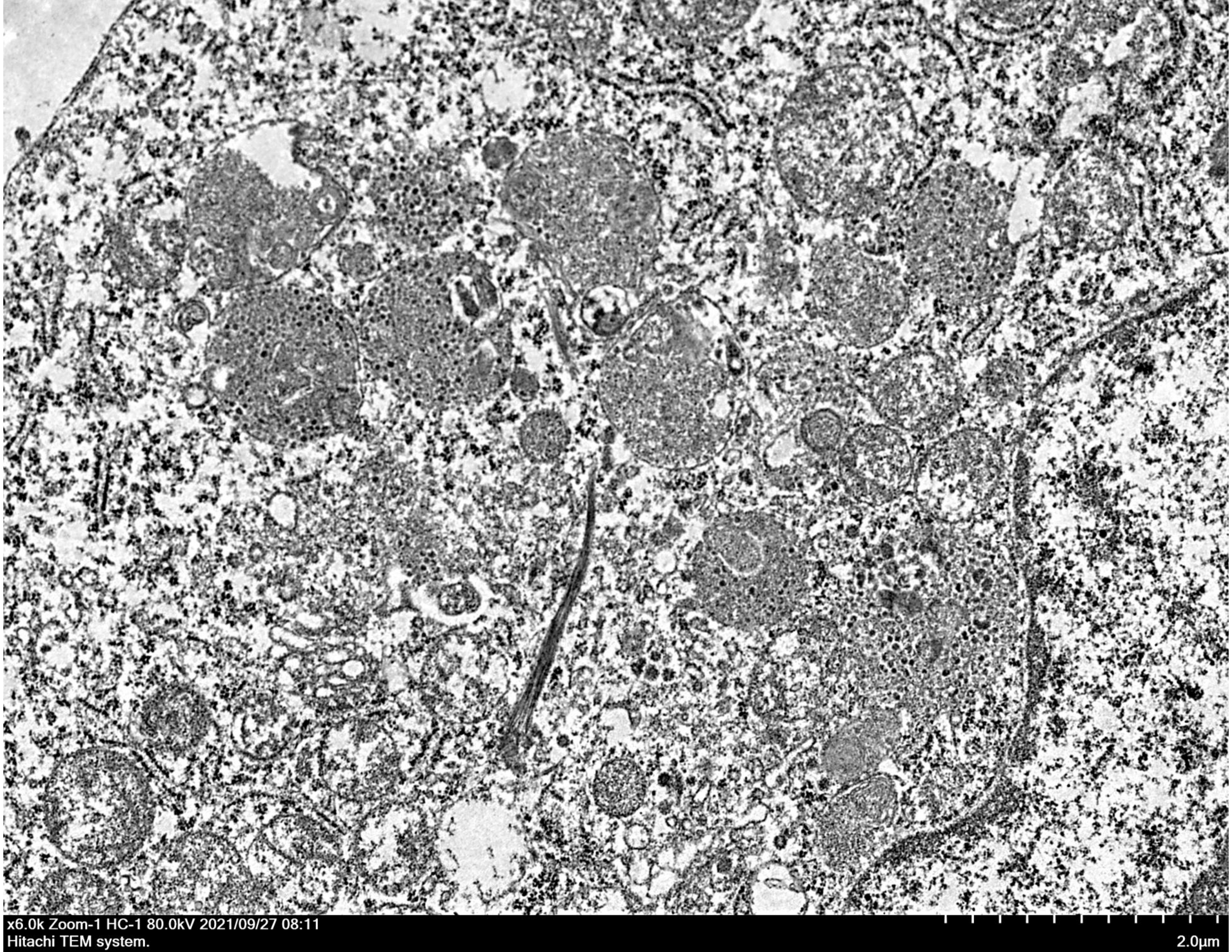

HepAD38
Dox (-)

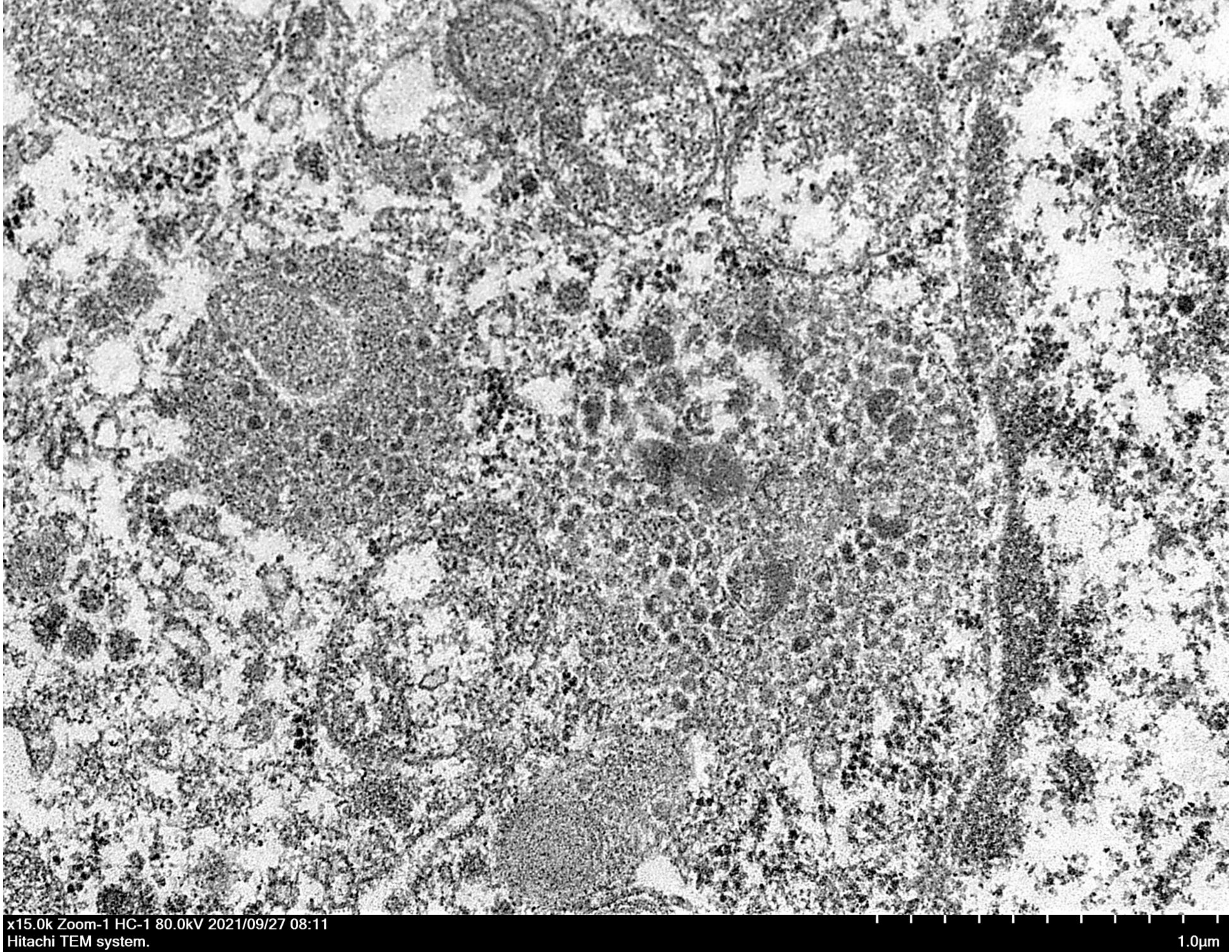
x15.0k Zoom-1 HC-1 80.0kV 2021/09/27 08:11
Hitachi TEM system.
1.0µm

HepAD38
Dox (-)

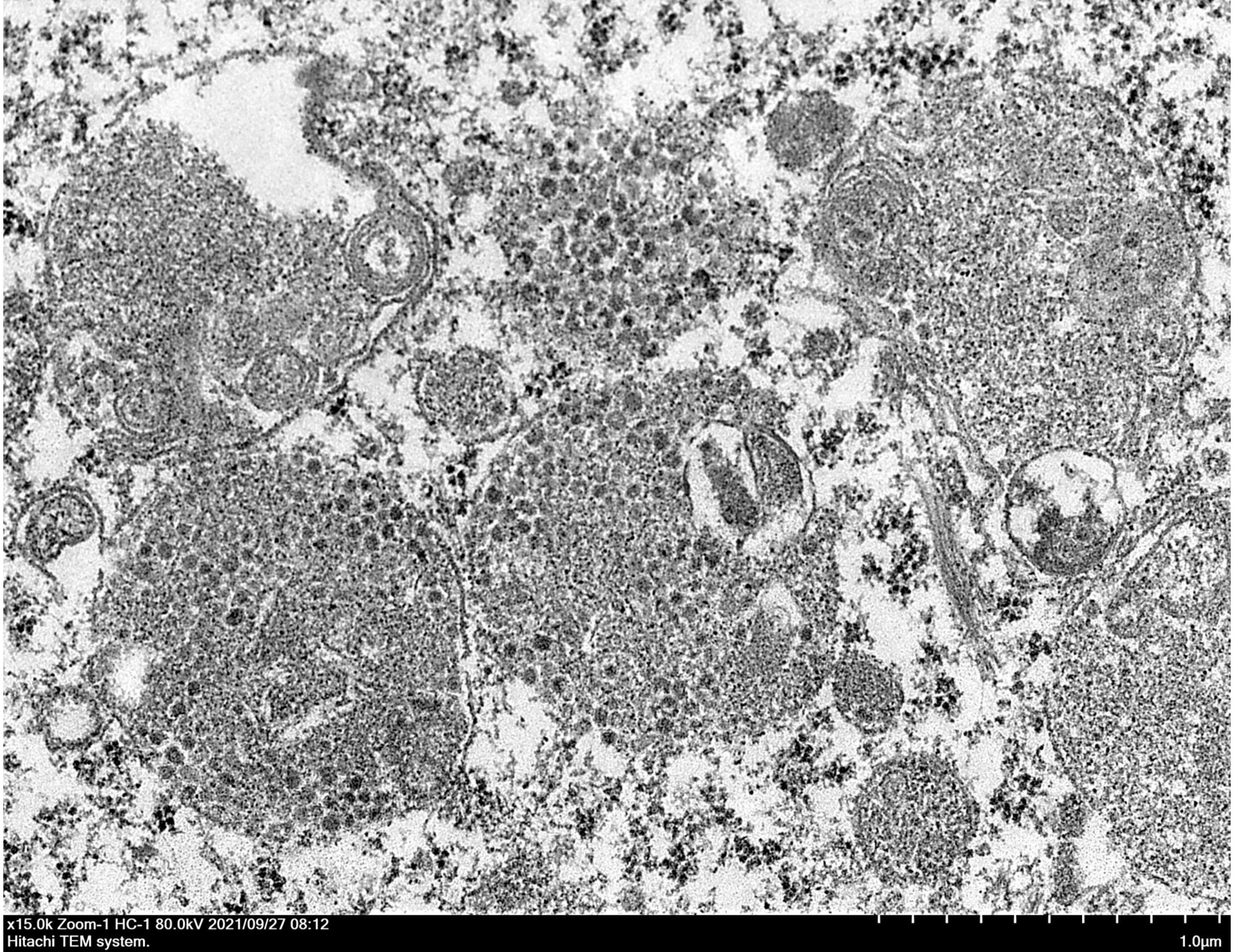
x15.0k Zoom-1 HC-1 80.0kV 2021/09/27 08:12
Hitachi TEM system.
1.0μm

HepAD38
Dox (+)

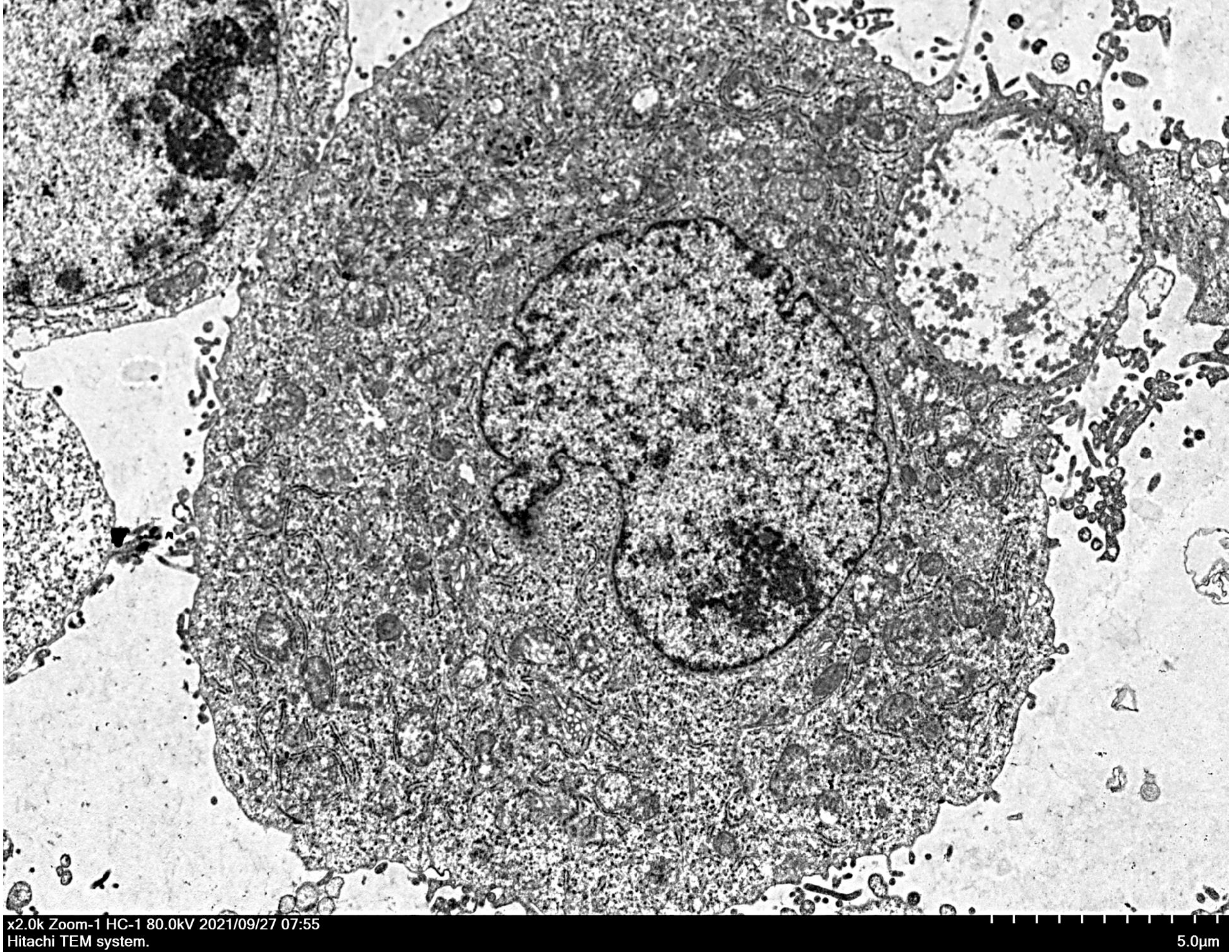

HepAD38
Dox (+)

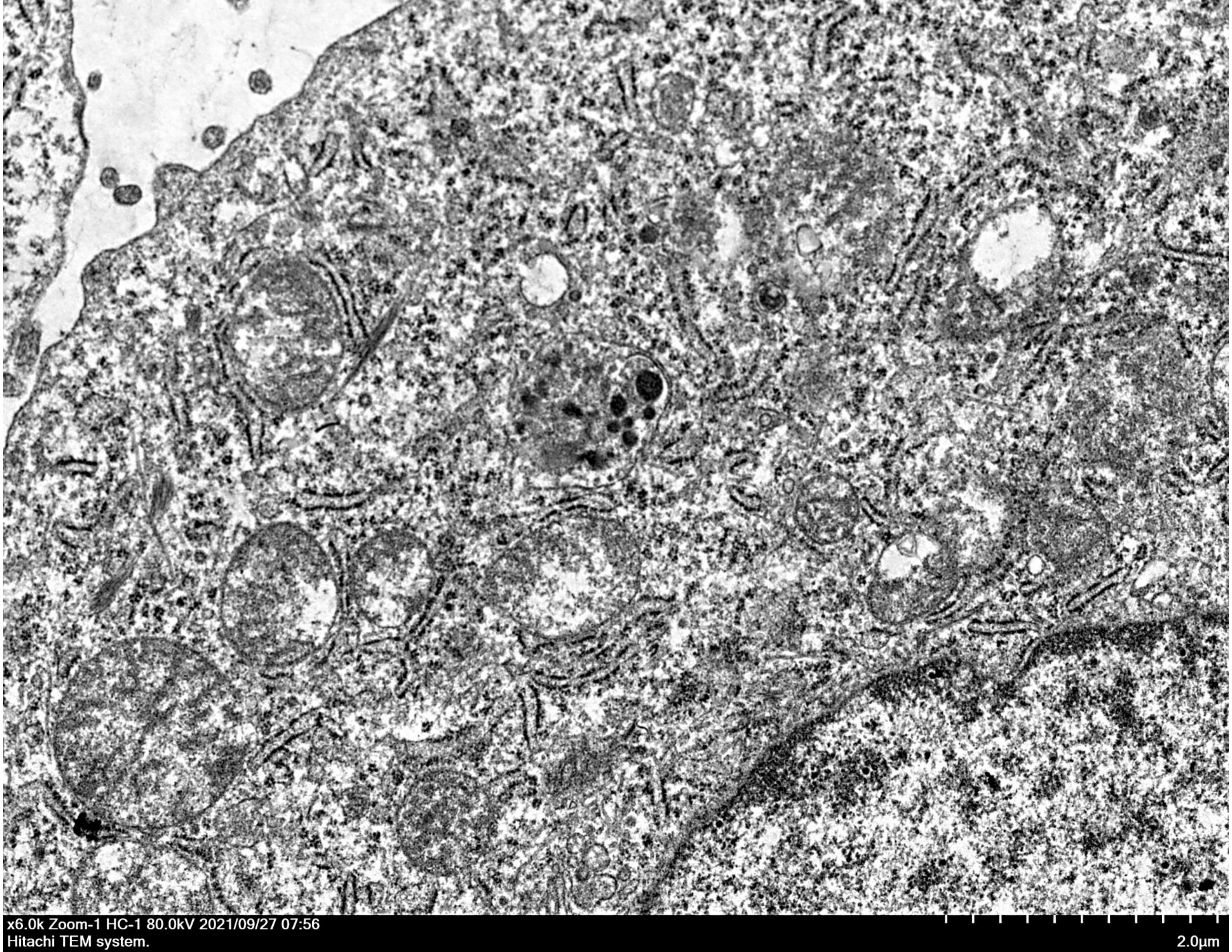

HepAD38
Dox (+)

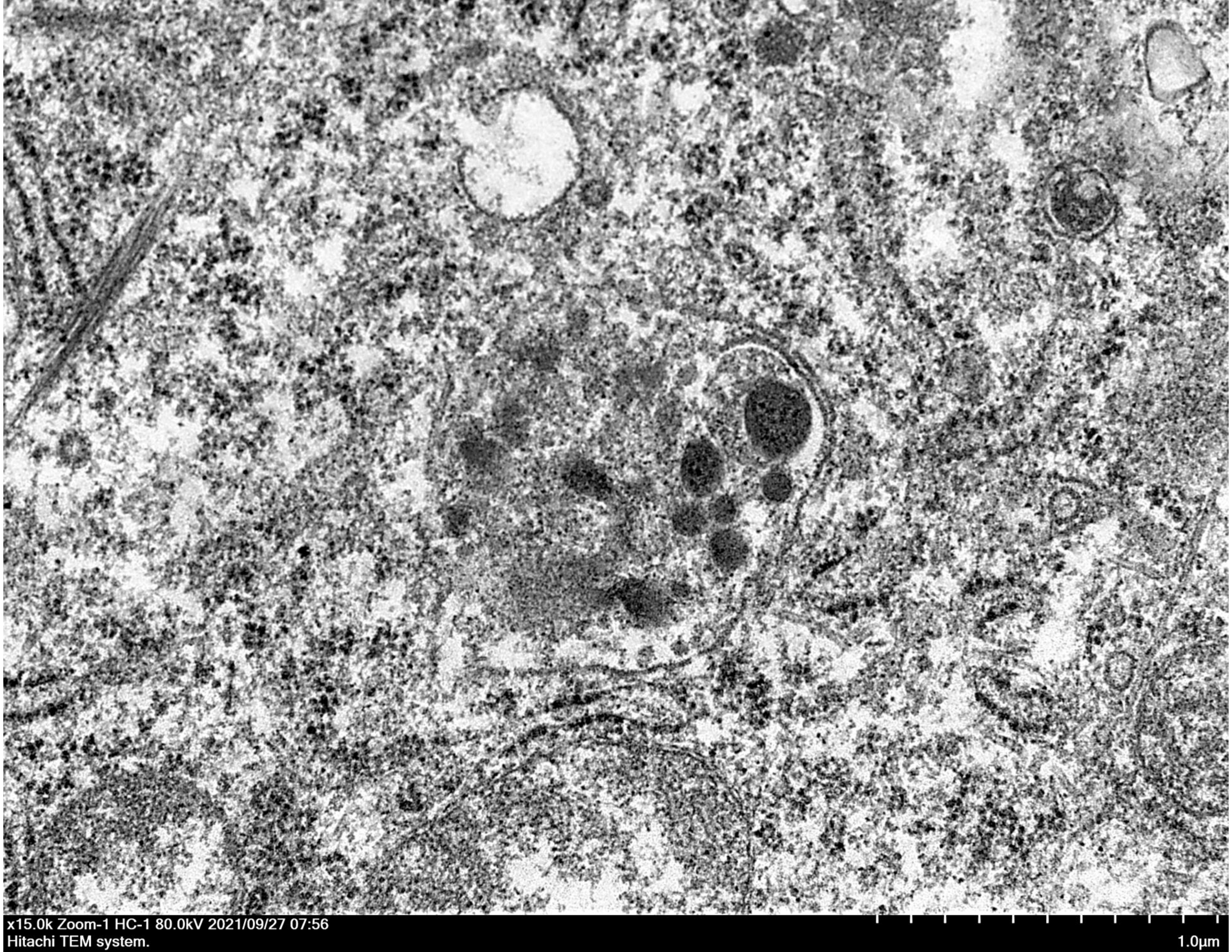
x15.0k Zoom-1 HC-1 80.0kV 2021/09/27 07:56
Hitachi TEM system.
1.0μm

HepAD38
Dox (+)

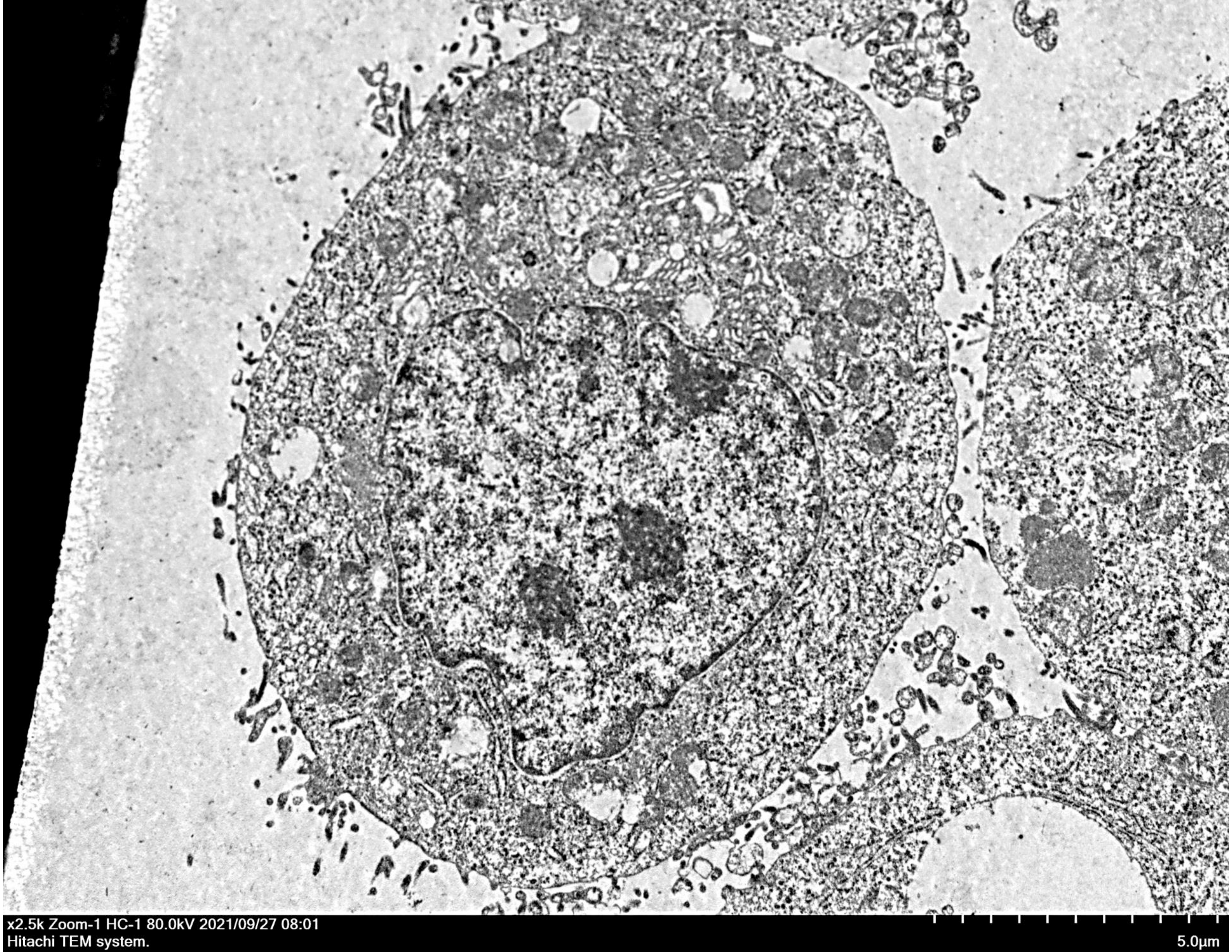

HepAD38
Dox (+)

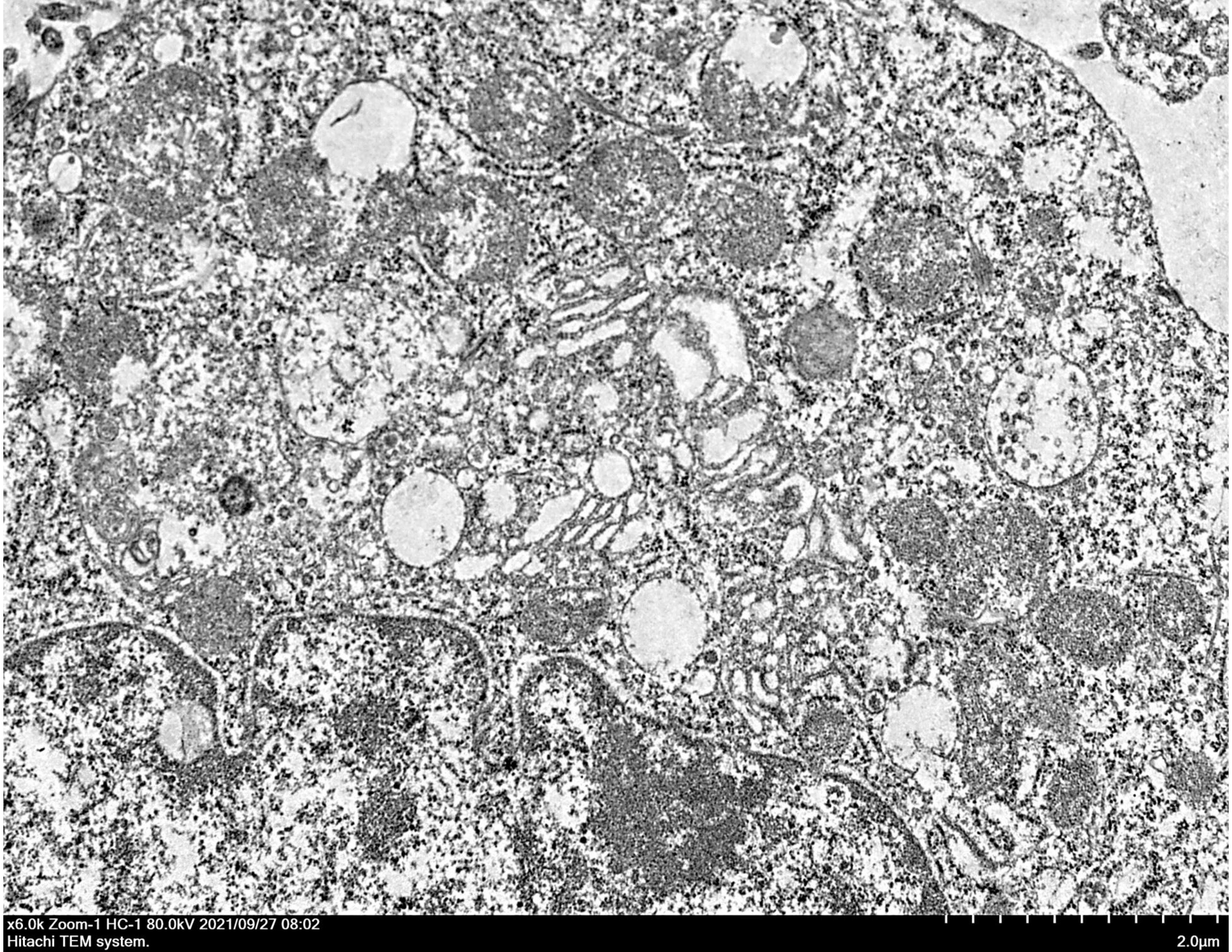

HepAD38
Dox (+)

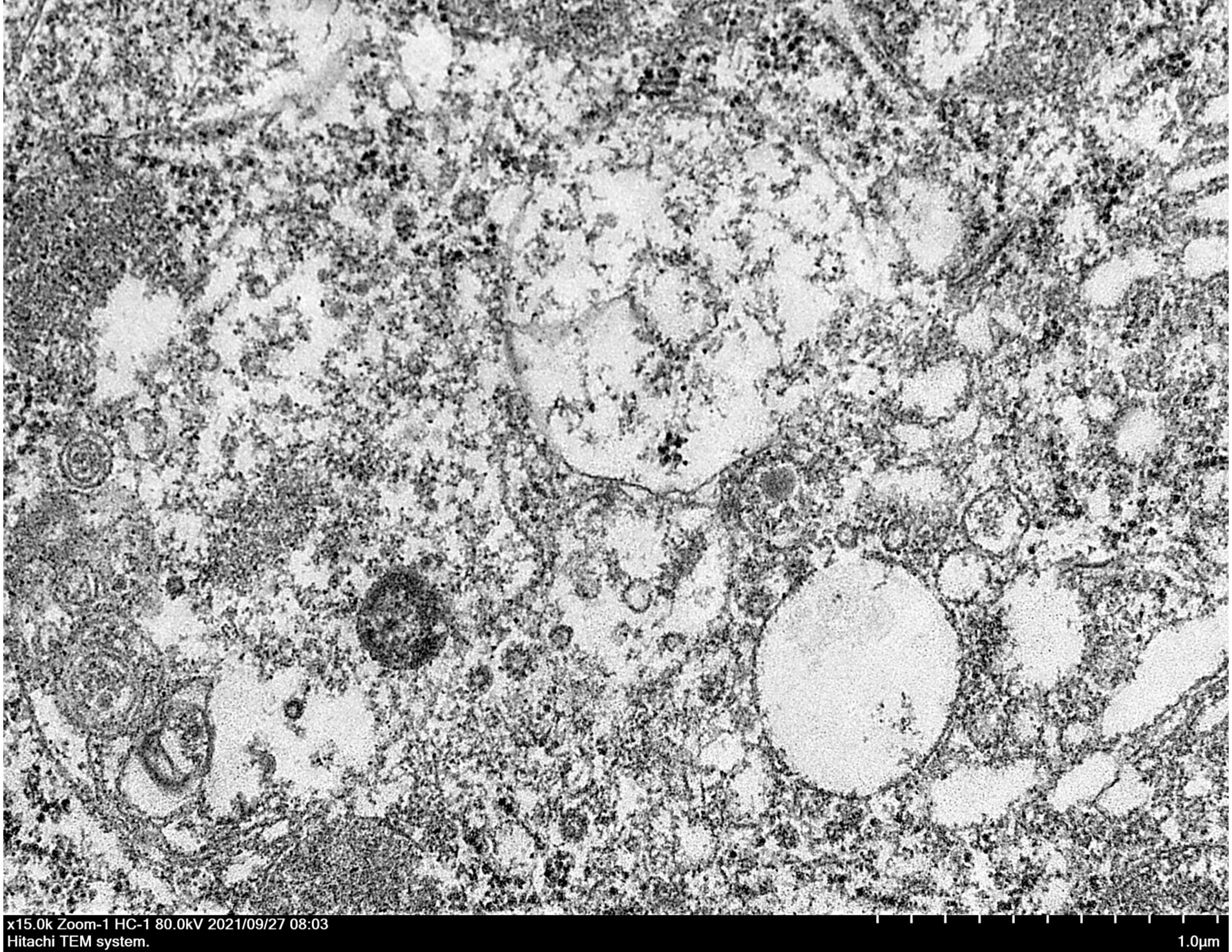
x15.0k Zoom-1 HC-1 80.0kV 2021/09/27 08:03
Hitachi TEM system.
1.0μm

HepAD38
Dox (+)

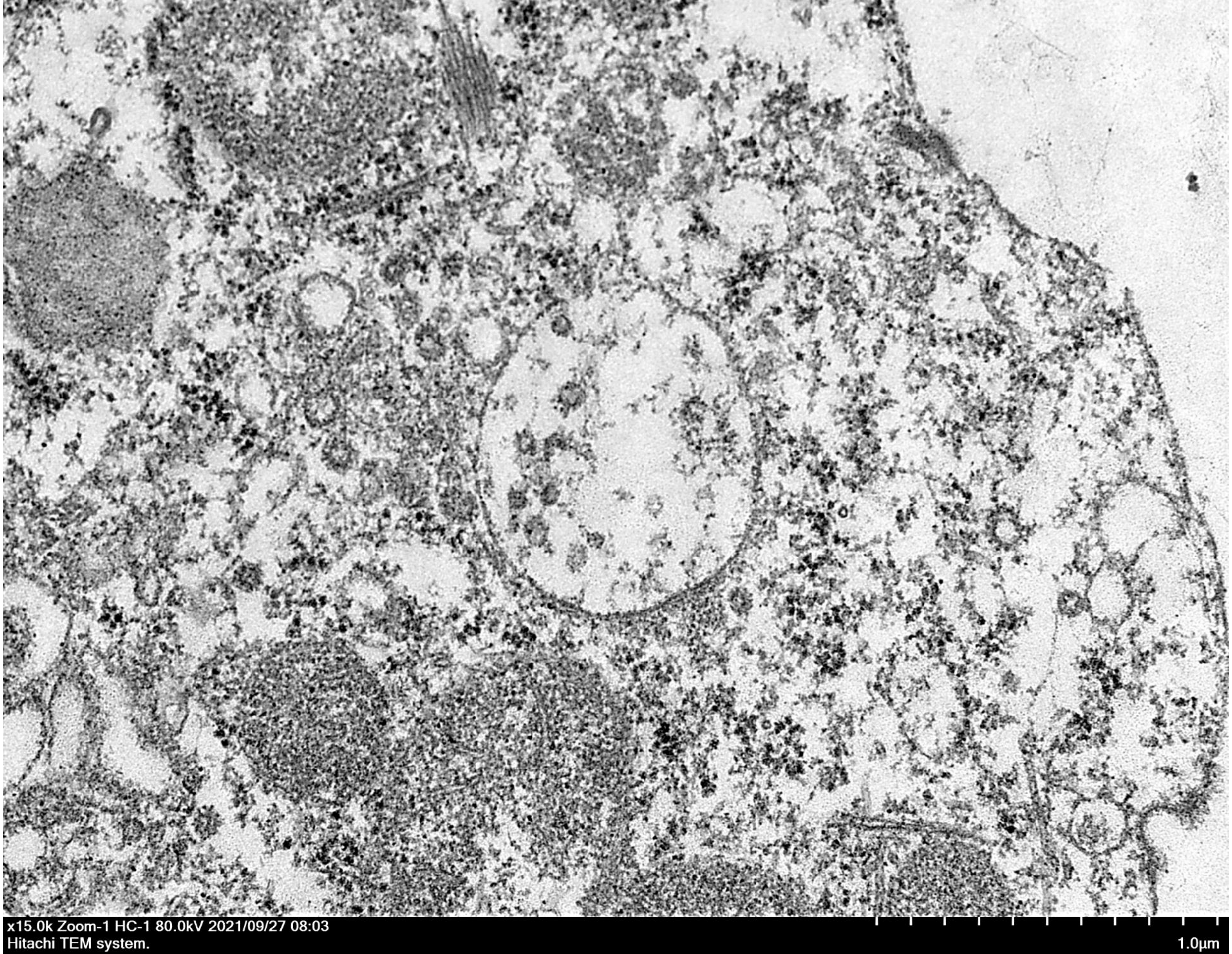
x15.0k Zoom-1 HC-1 80.0kV 2021/09/27 08:03
Hitachi TEM system.
1.0μm

HepAD38
Dox (+)

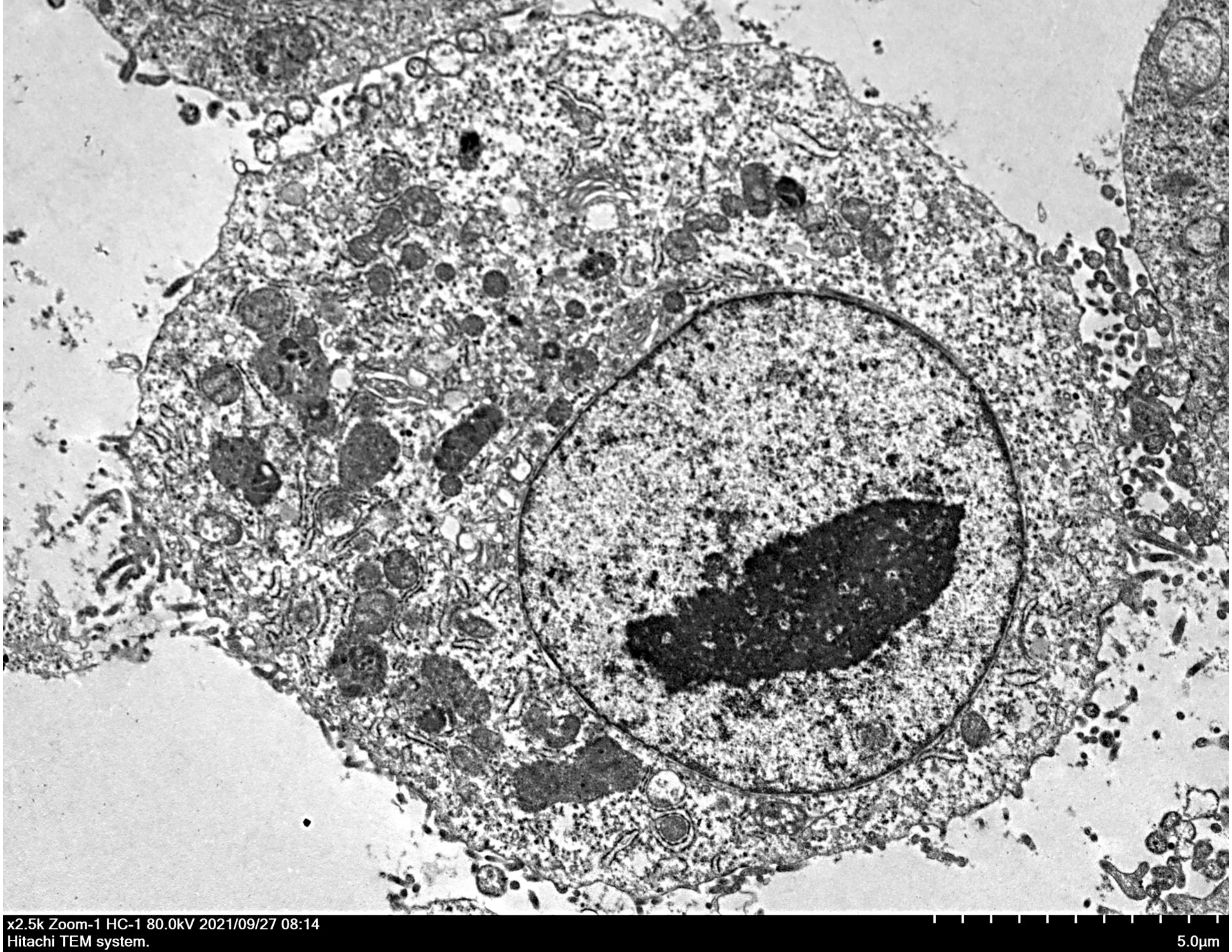

HepAD38
Dox (+)

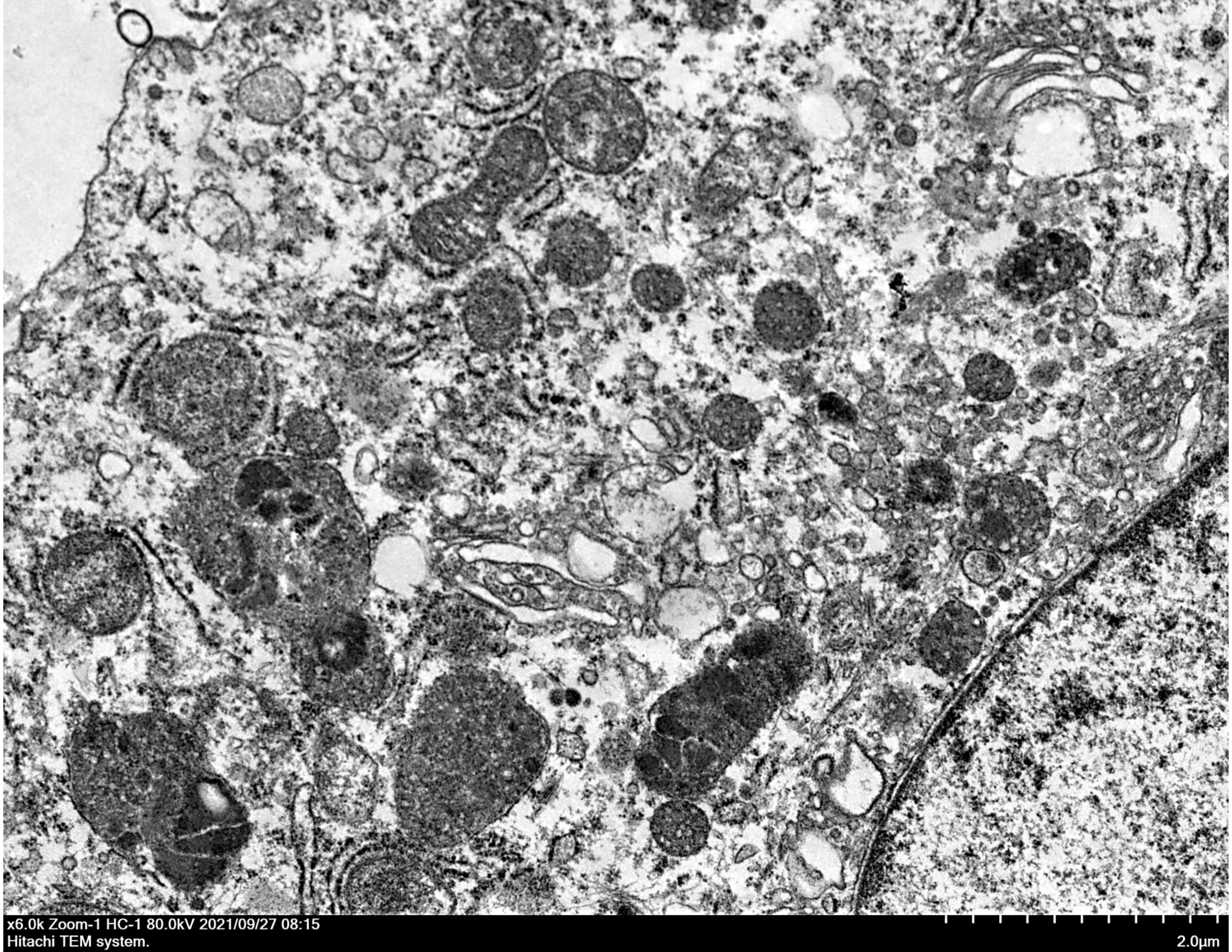

HepAD38
Dox (+)

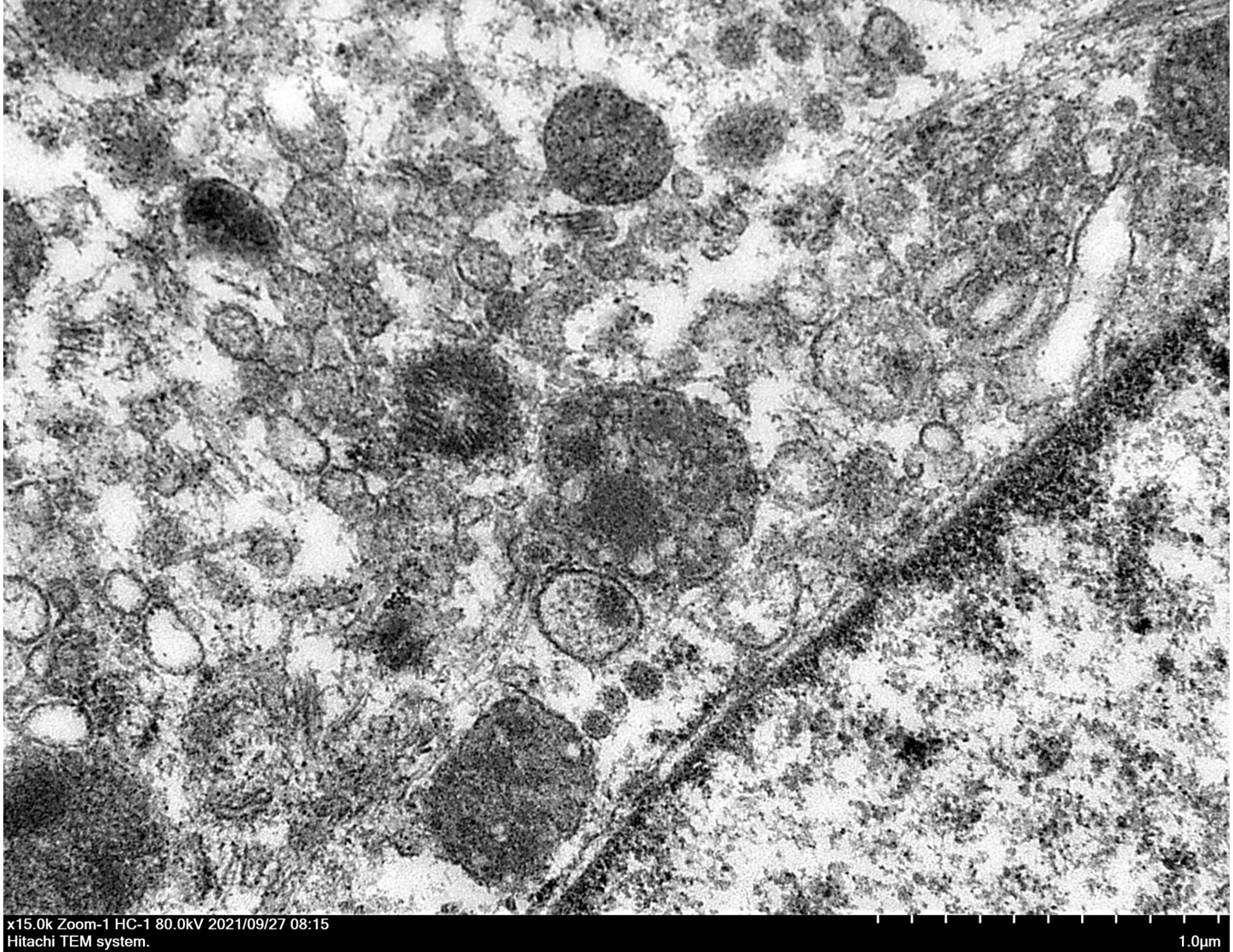
x15.0k Zoom-1 HC-1 80.0kV 2021/09/27 08:15
Hitachi TEM system.
1.0μm

HepAD38
Dox (+)

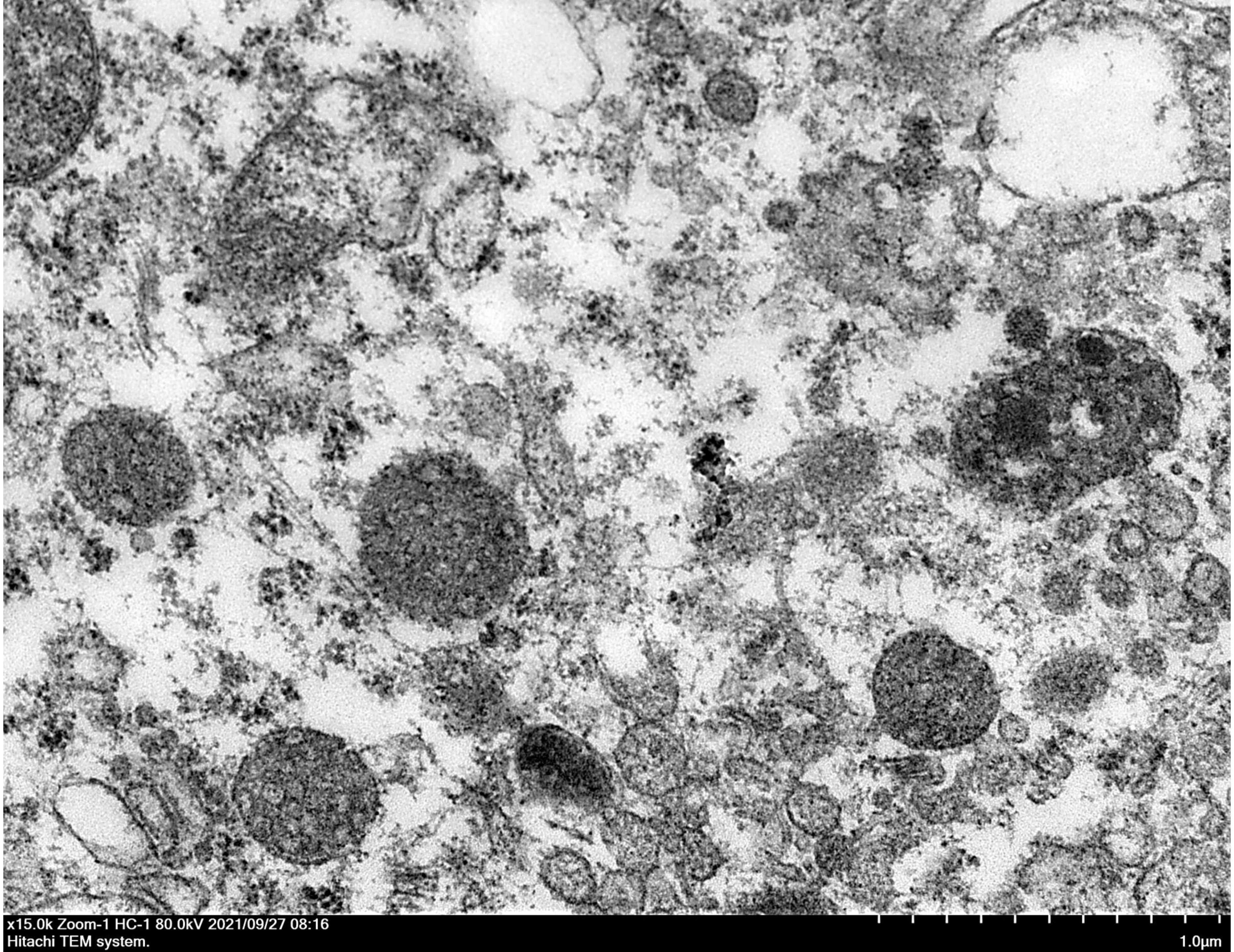
x15.0k Zoom-1 HC-1 80.0kV 2021/09/27 08:16
Hitachi TEM system.
1.0μm

Fig 2C

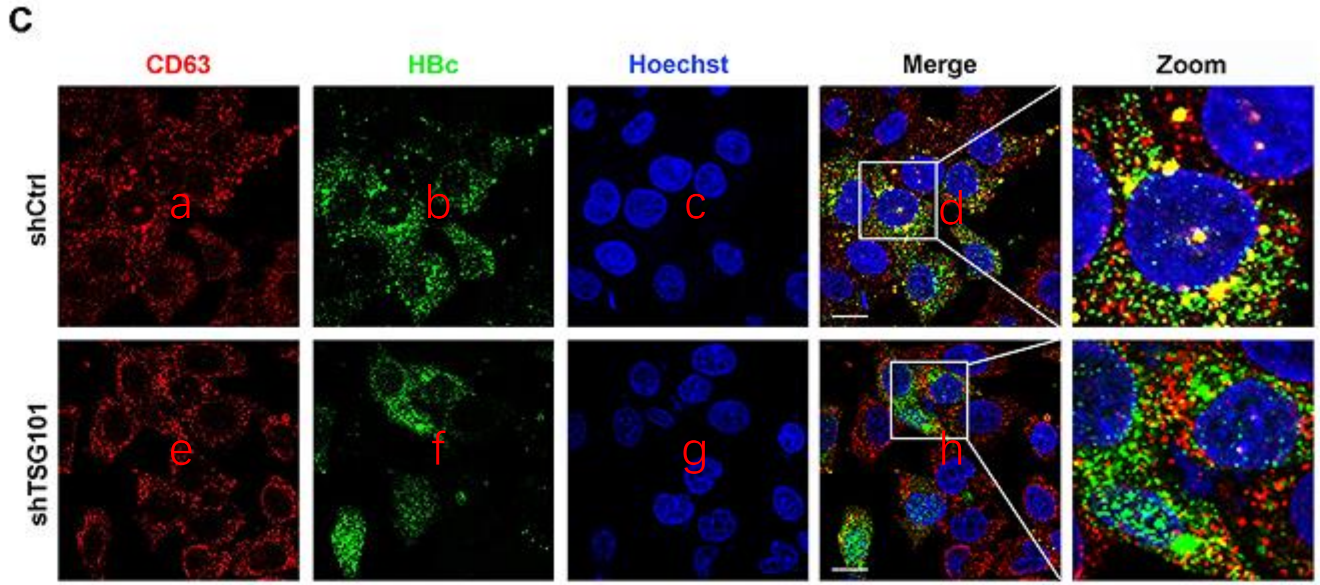

a

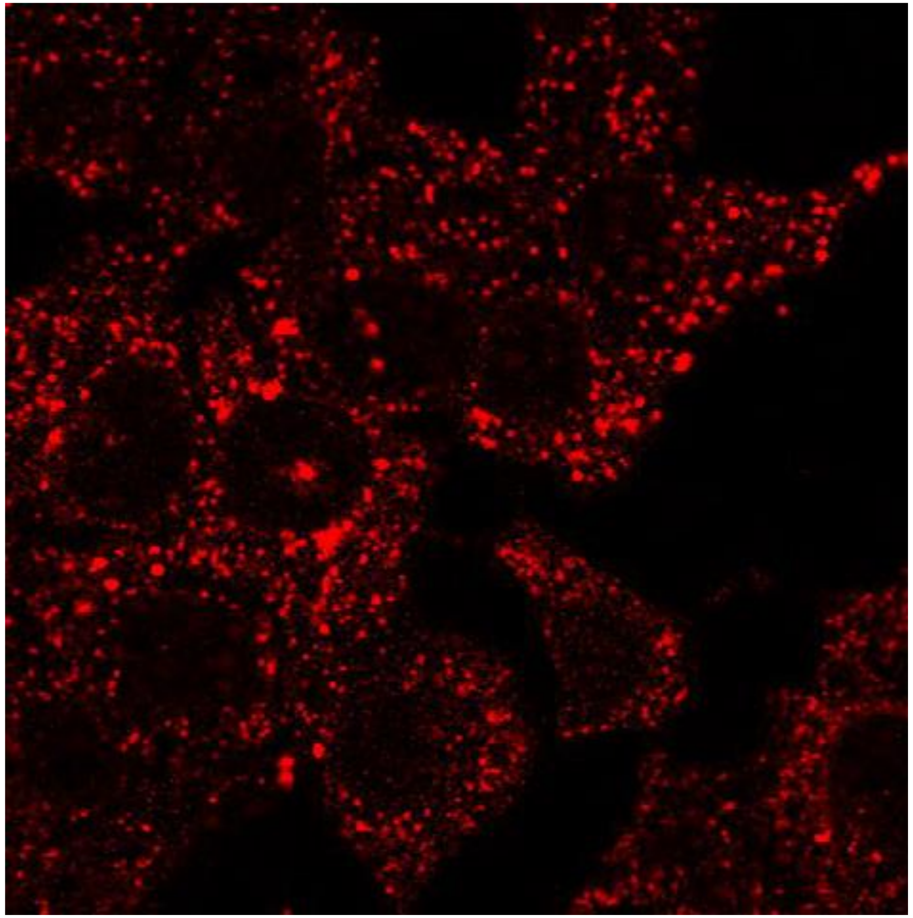

b

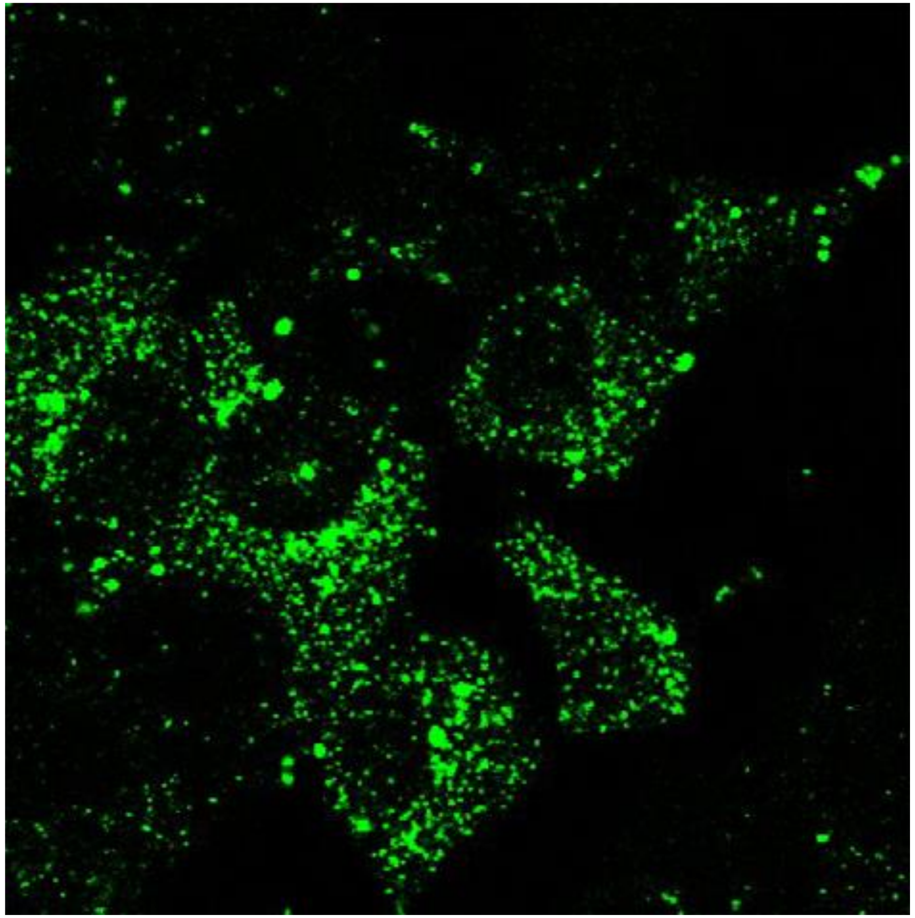

C

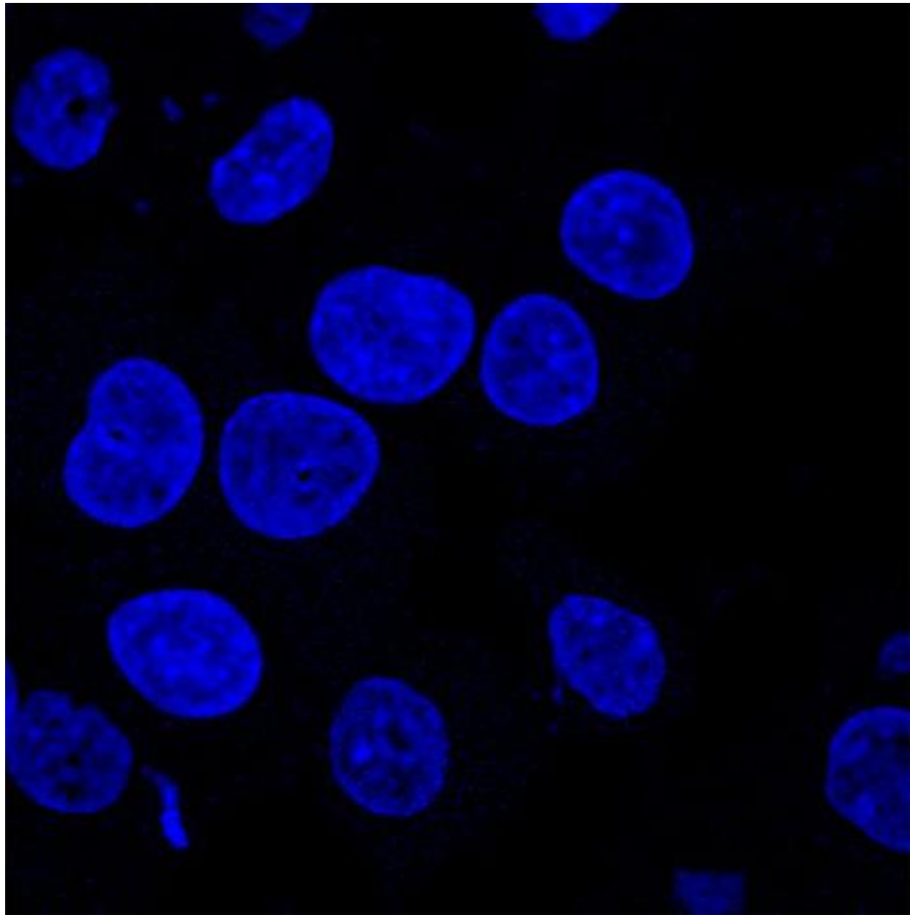

d

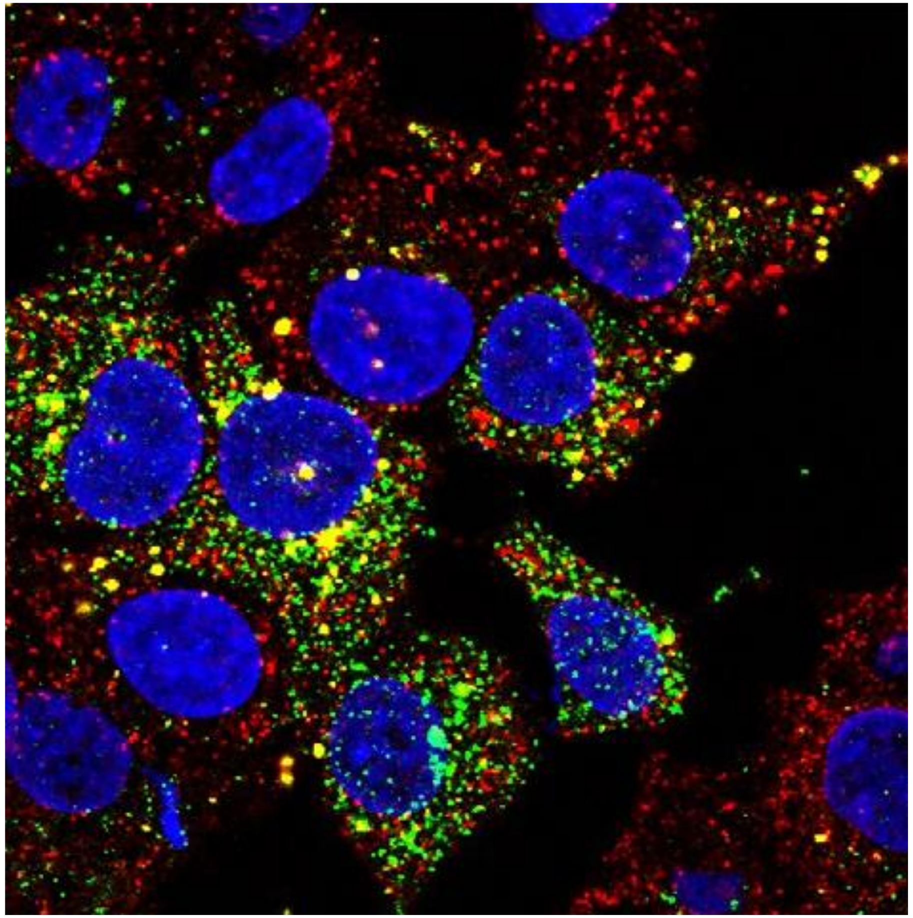

e

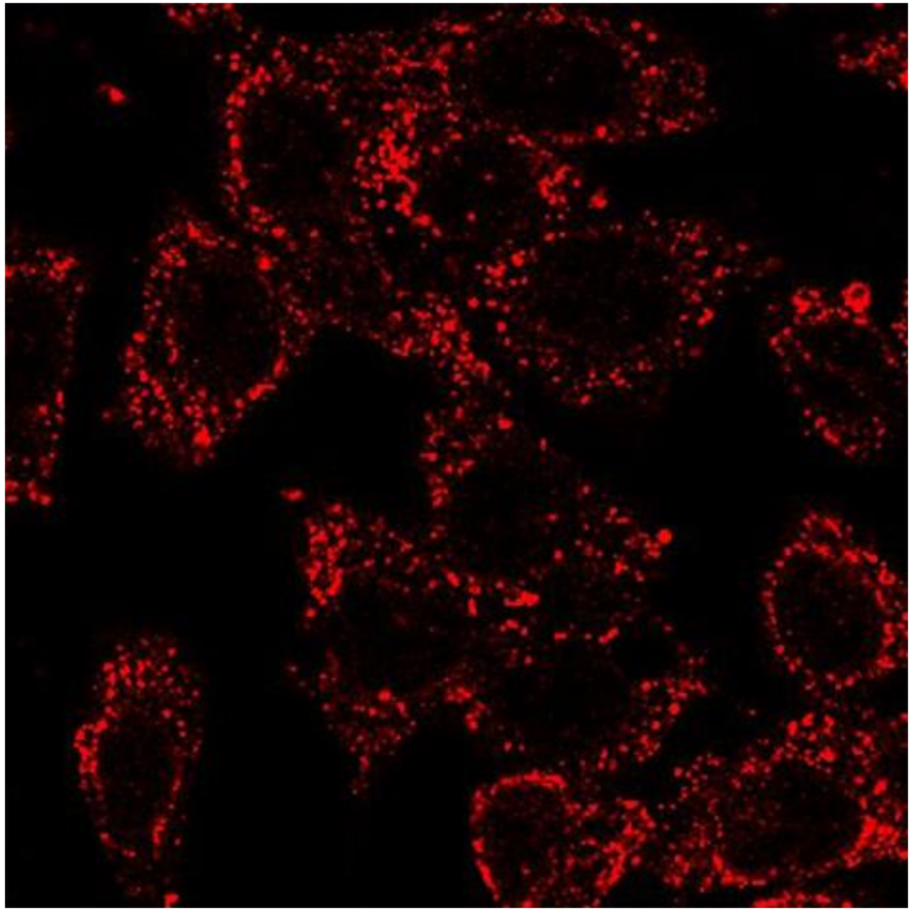

f

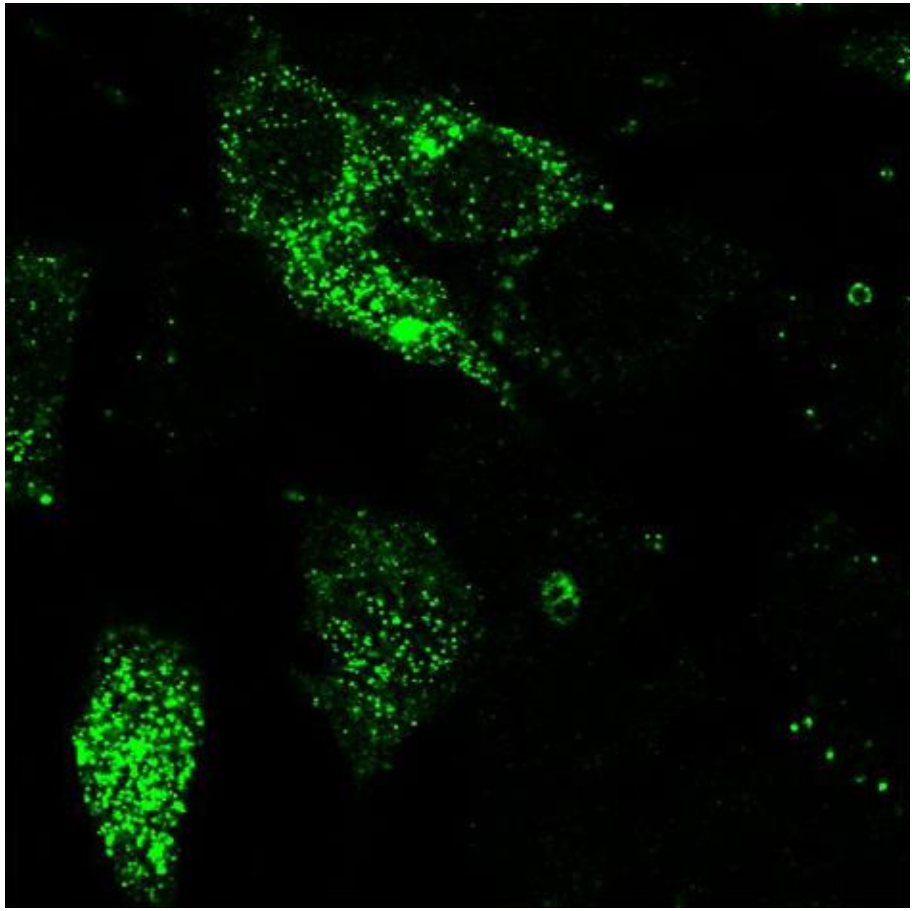

g

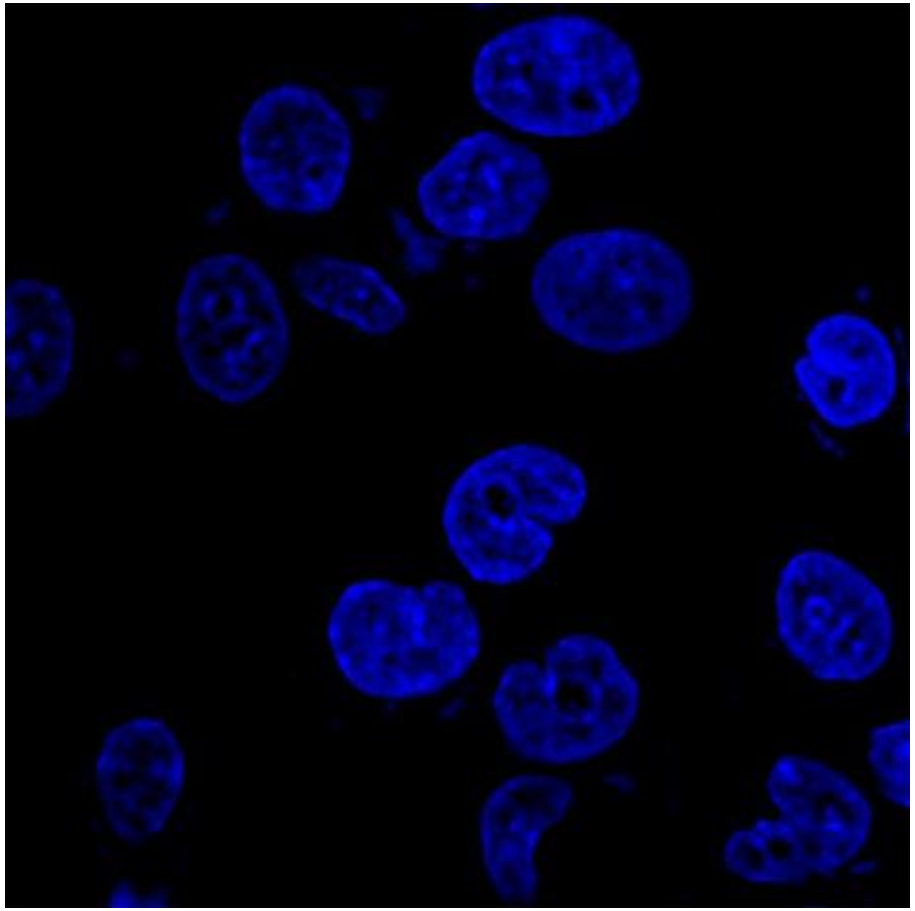

h

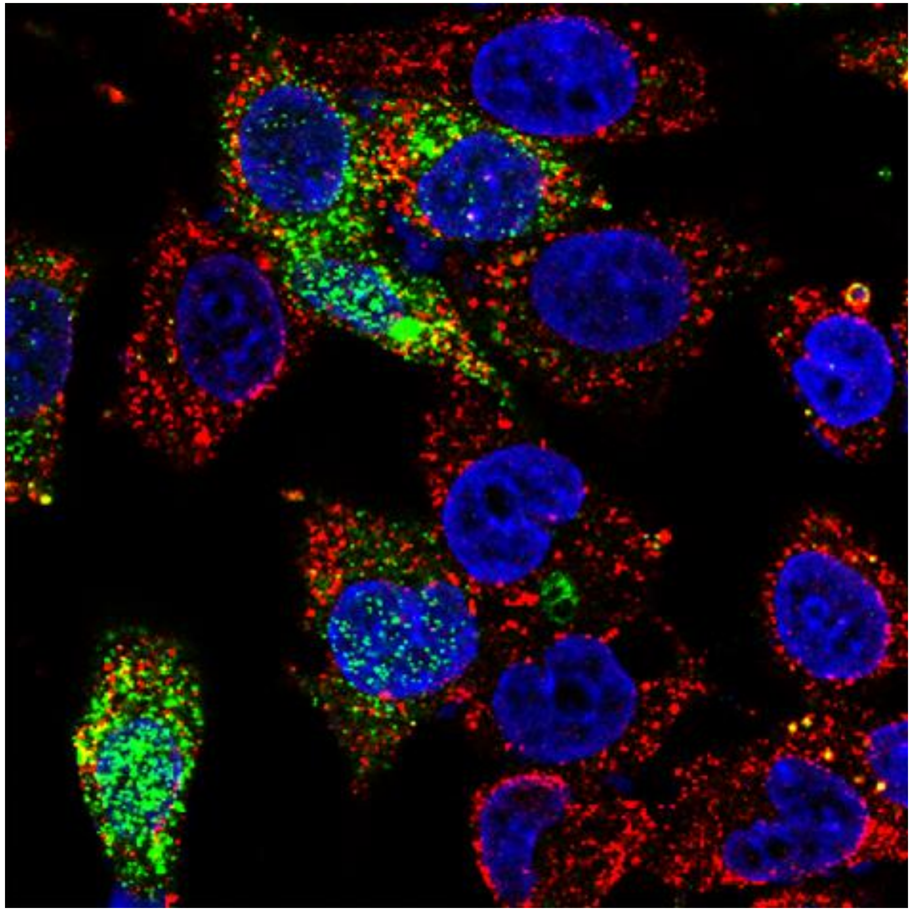

Fig 2D

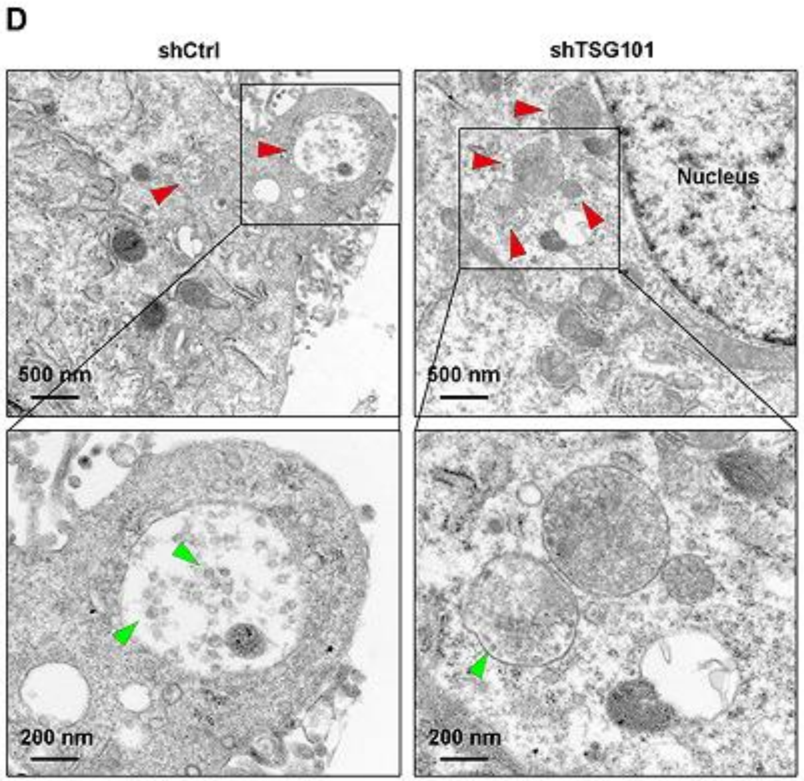

shCtrl

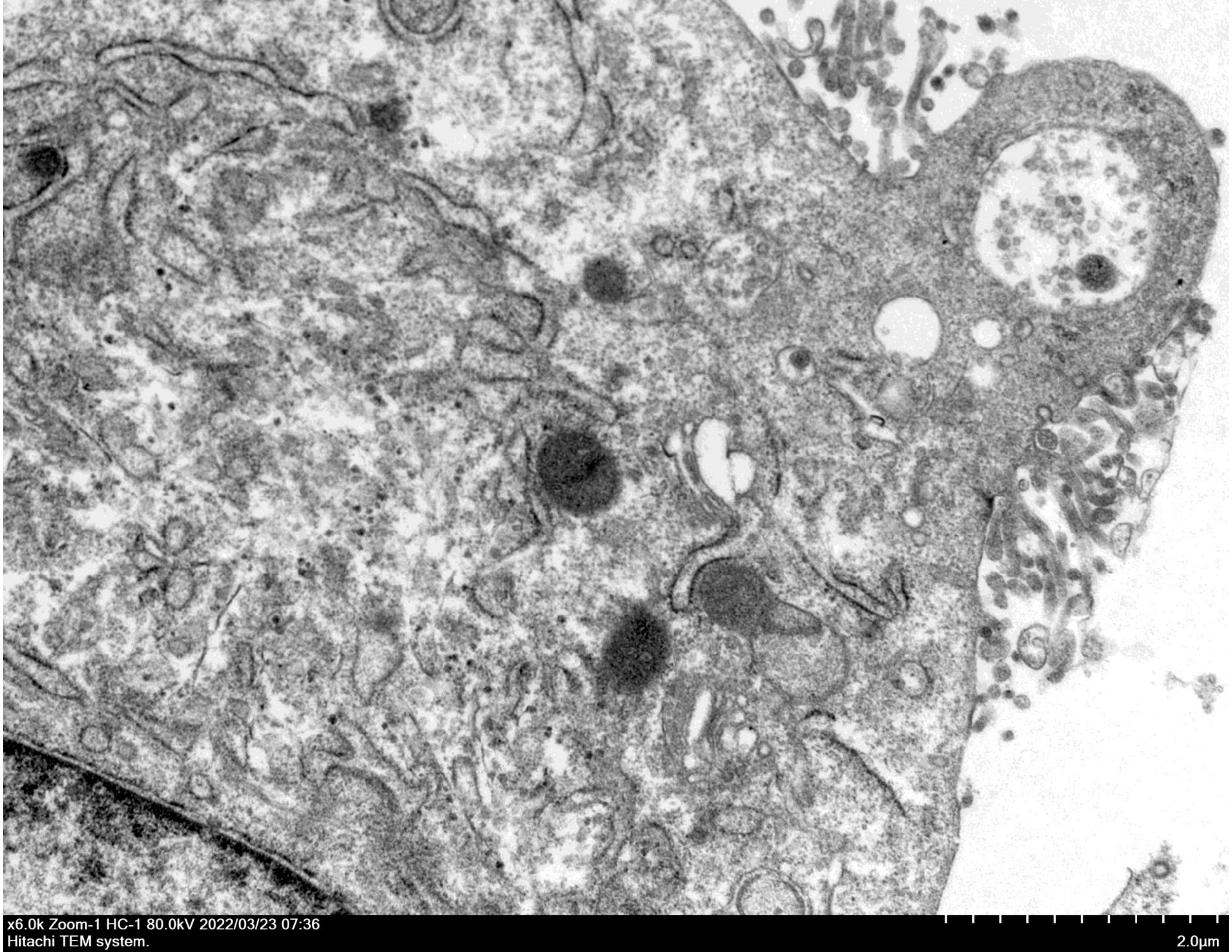

shCtrl

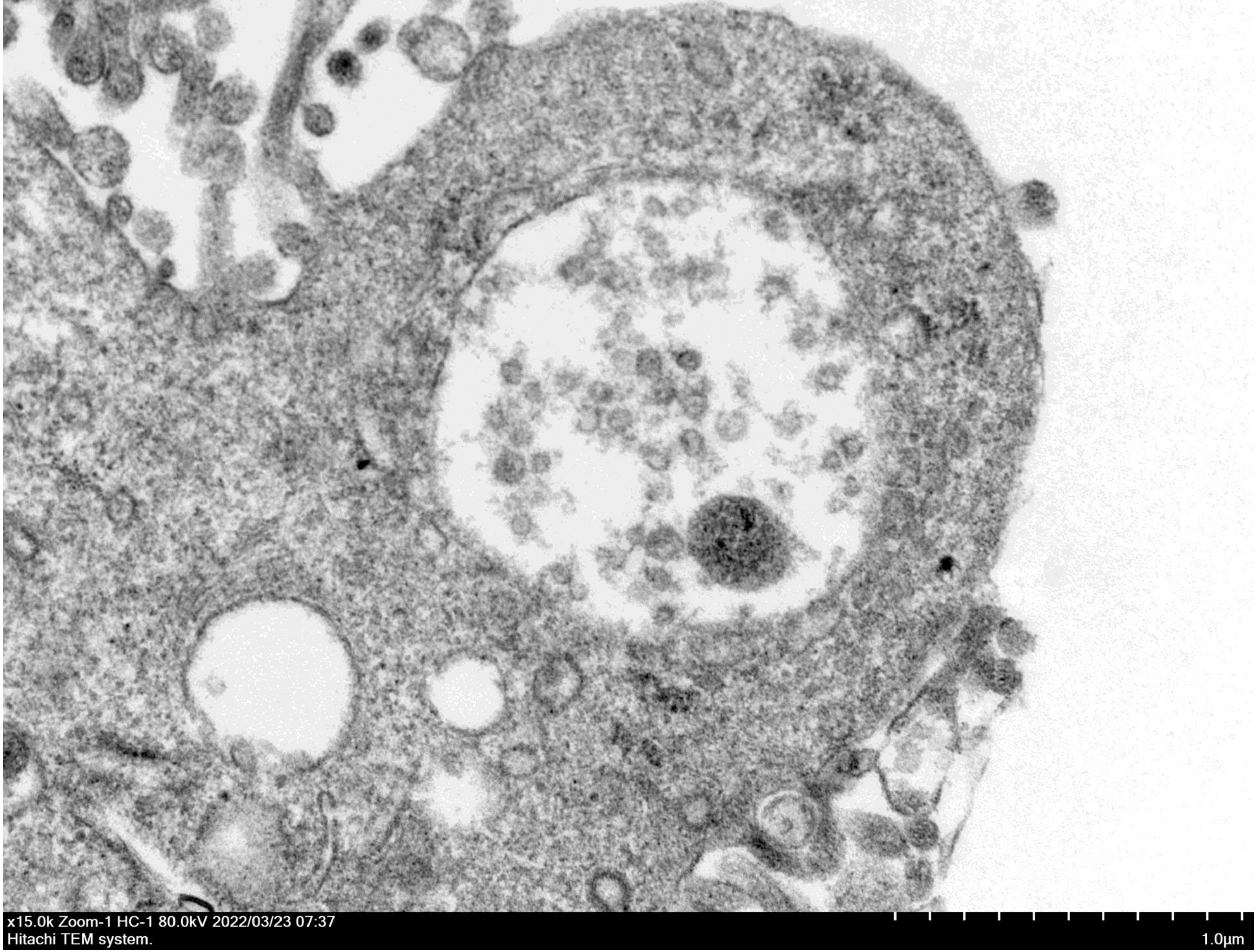
x15.0k Zoom-1 HC-1 80.0kV 2022/03/23 07:37
Hitachi TEM system.
1.0μm

shCtrl

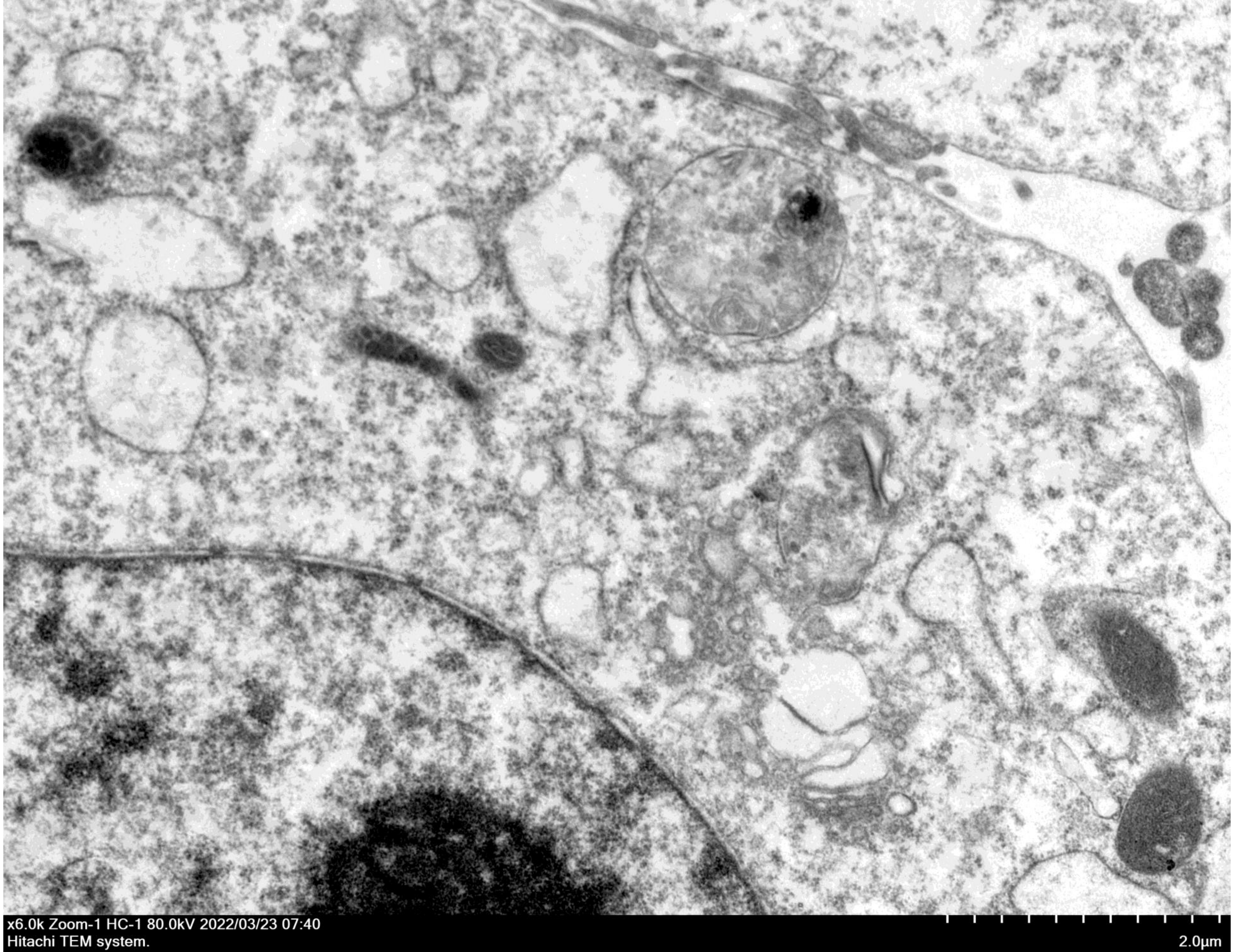

shCtrl

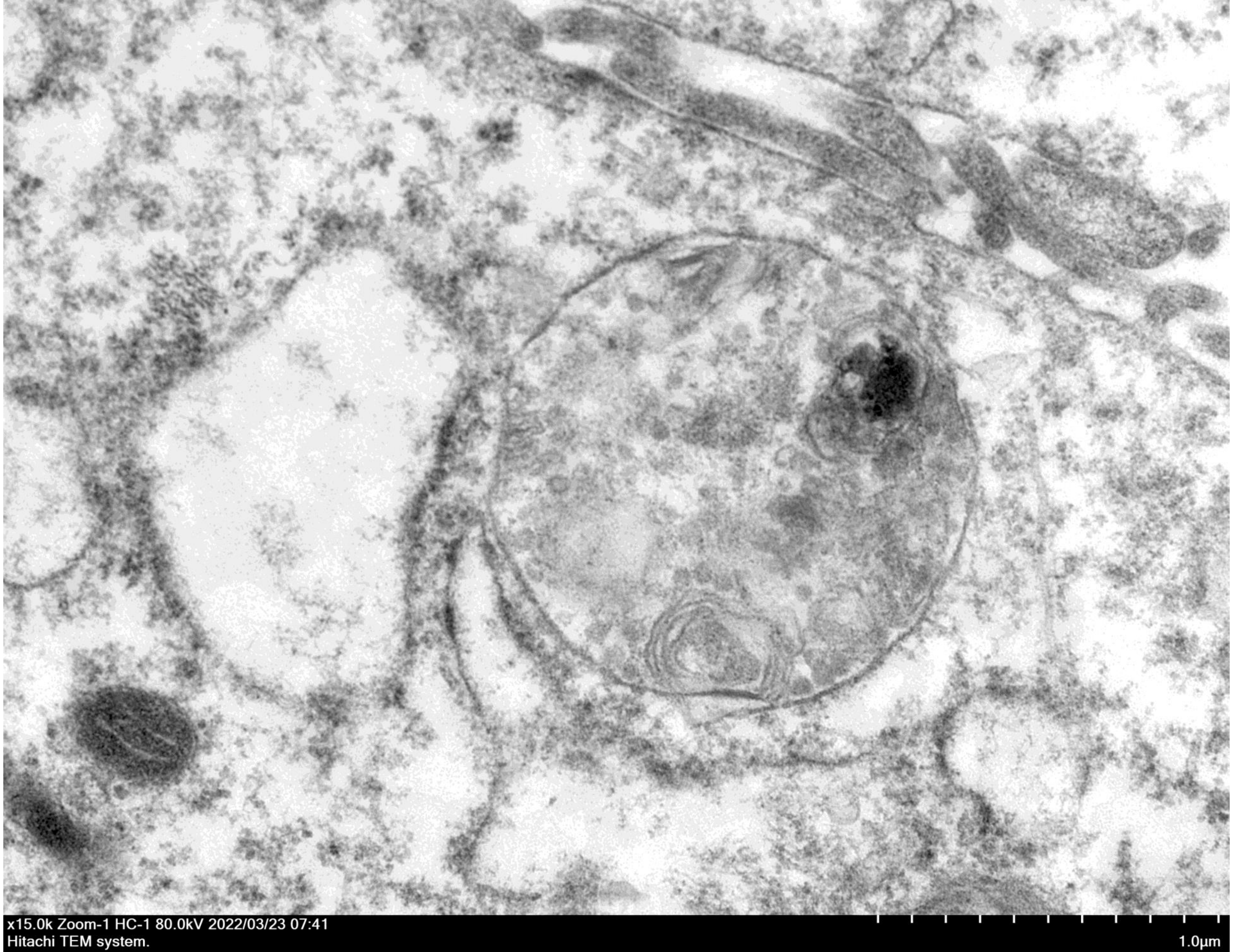
x15.0k Zoom-1 HC-1 80.0kV 2022/03/23 07:41
Hitachi TEM system.
1.0μm

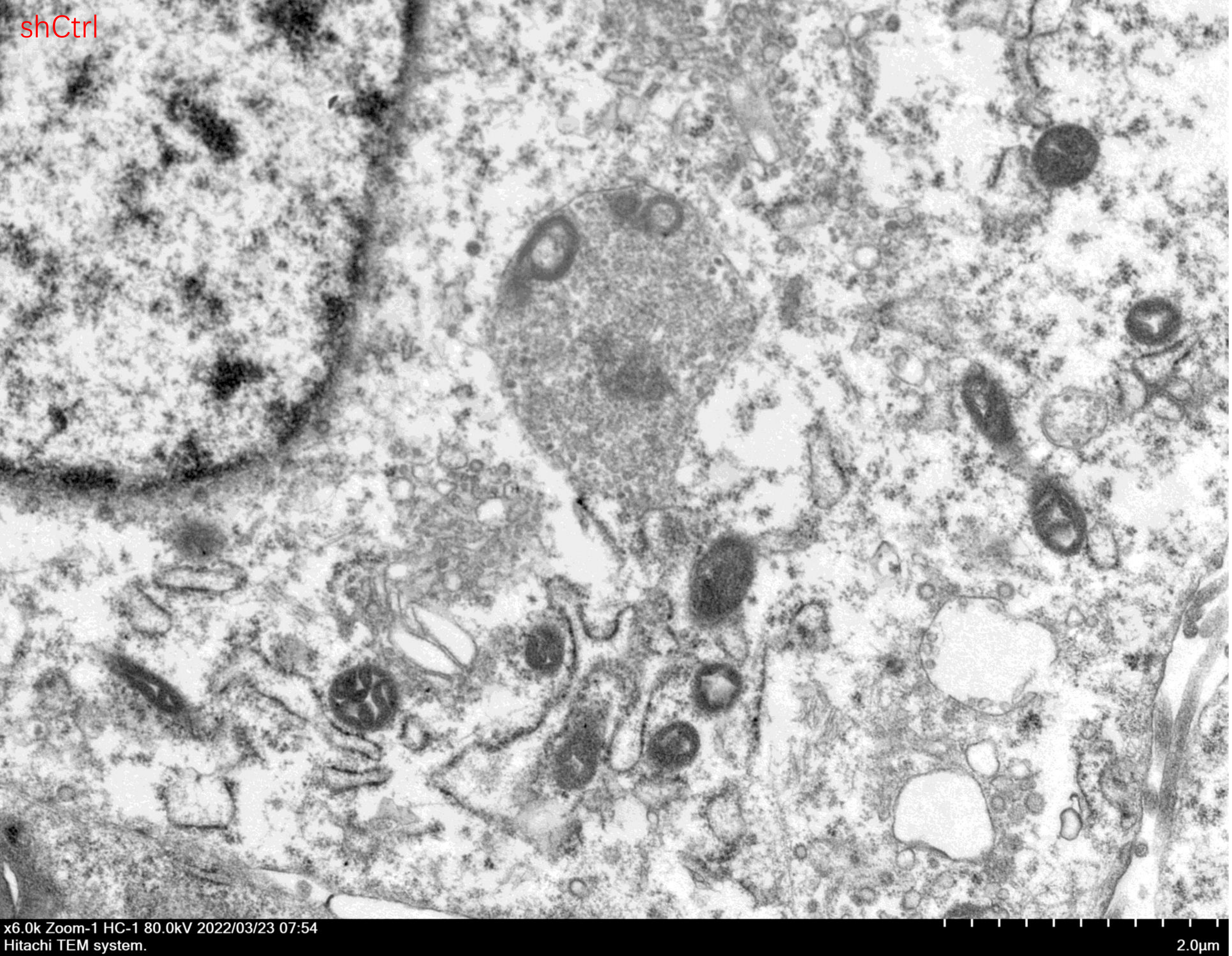
shCtrl
x6.0k Zoom-1 HC-1 80.0kV 2022/03/23 07:54
Hitachi TEM system.
2.0μm

shCtrl

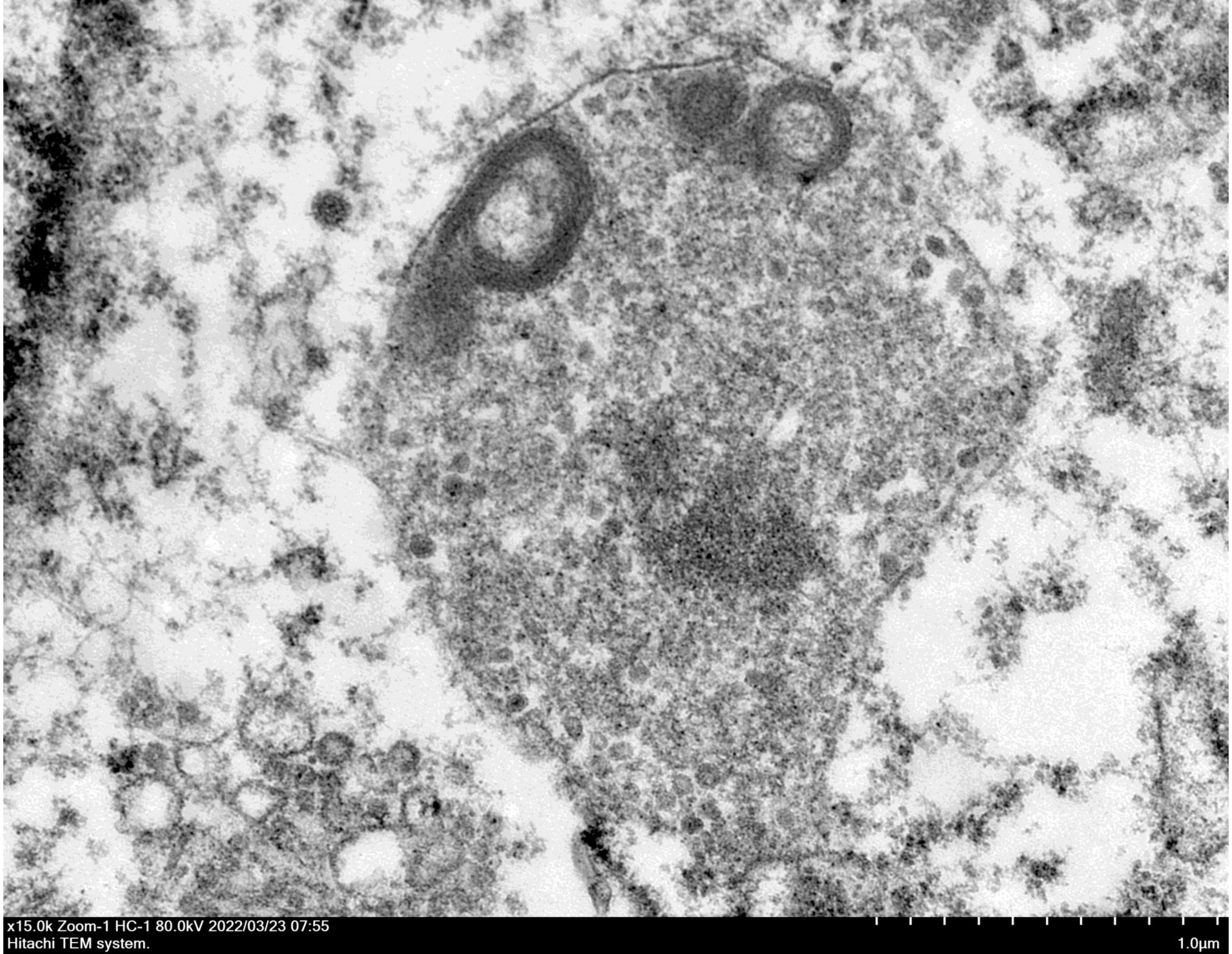
x15.0k Zoom-1 HC-1 80.0kV 2022/03/23 07:55
Hitachi TEM system.
1.0μm

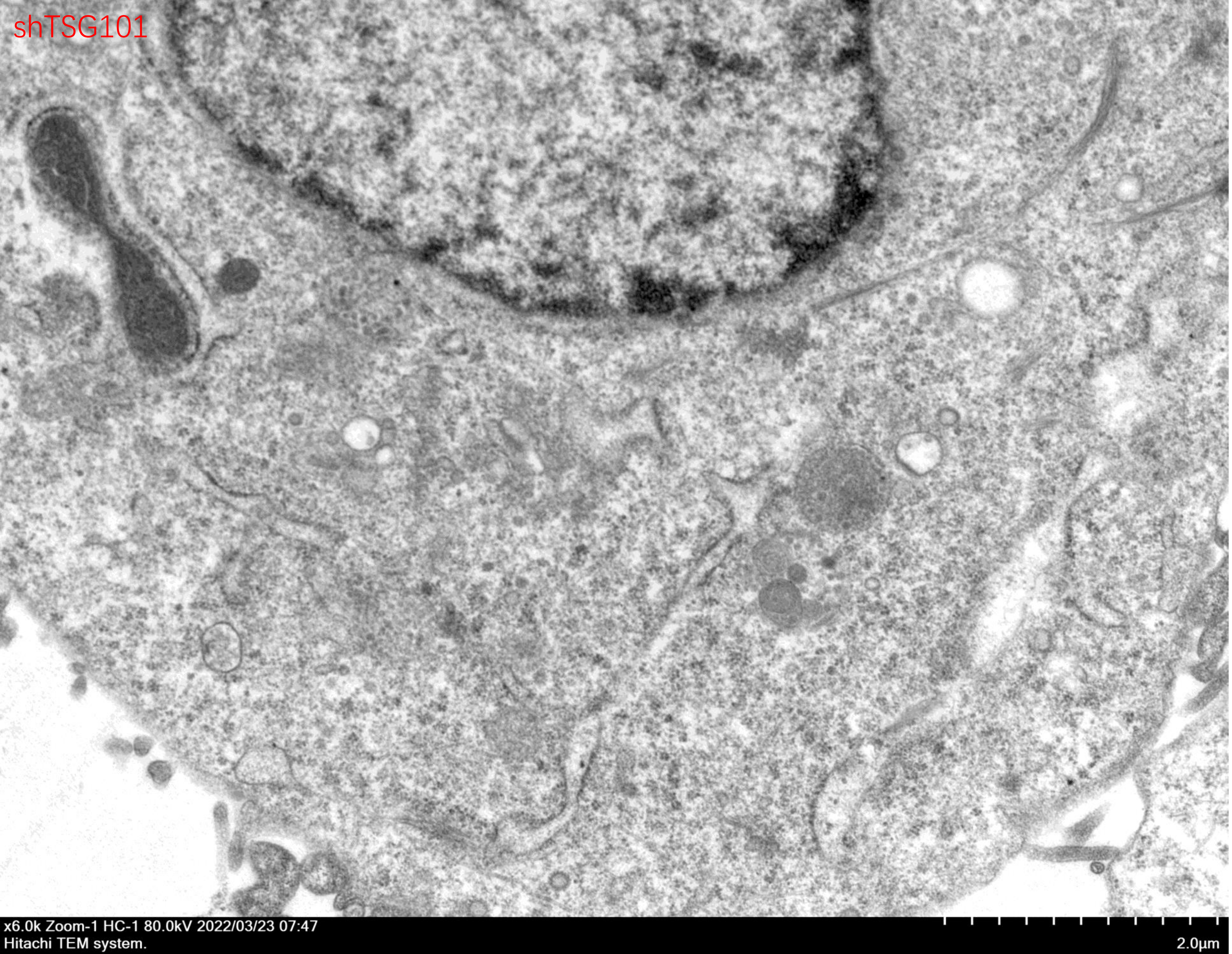
shTSG101
x6.0k Zoom-1 HC-1 80.0kV 2022/03/23 07:47
Hitachi TEM system.
2.0μm

shTSG101

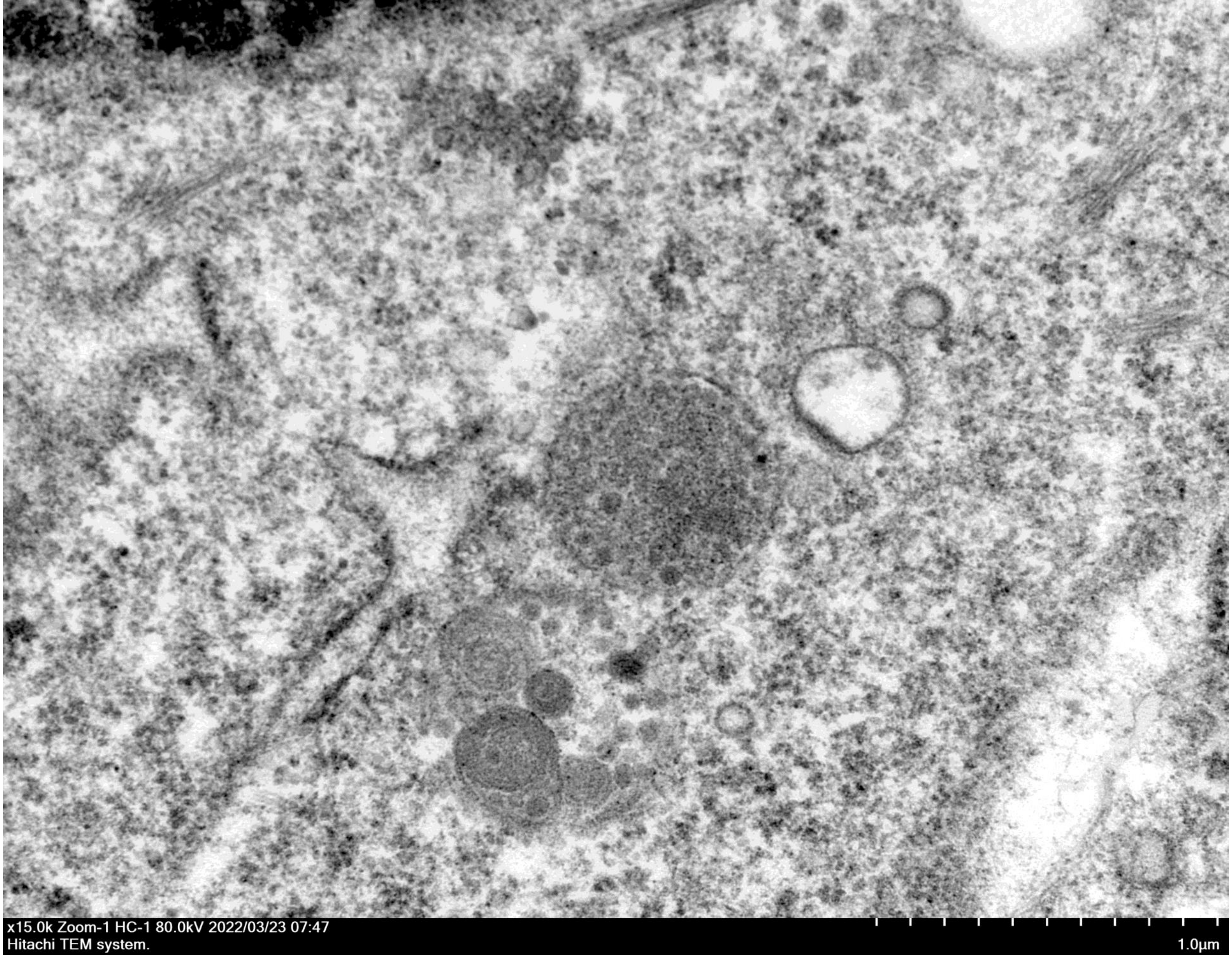
x15.0k Zoom-1 HC-1 80.0kV 2022/03/23 07:47
Hitachi TEM system.
1.0μm

shTSG101

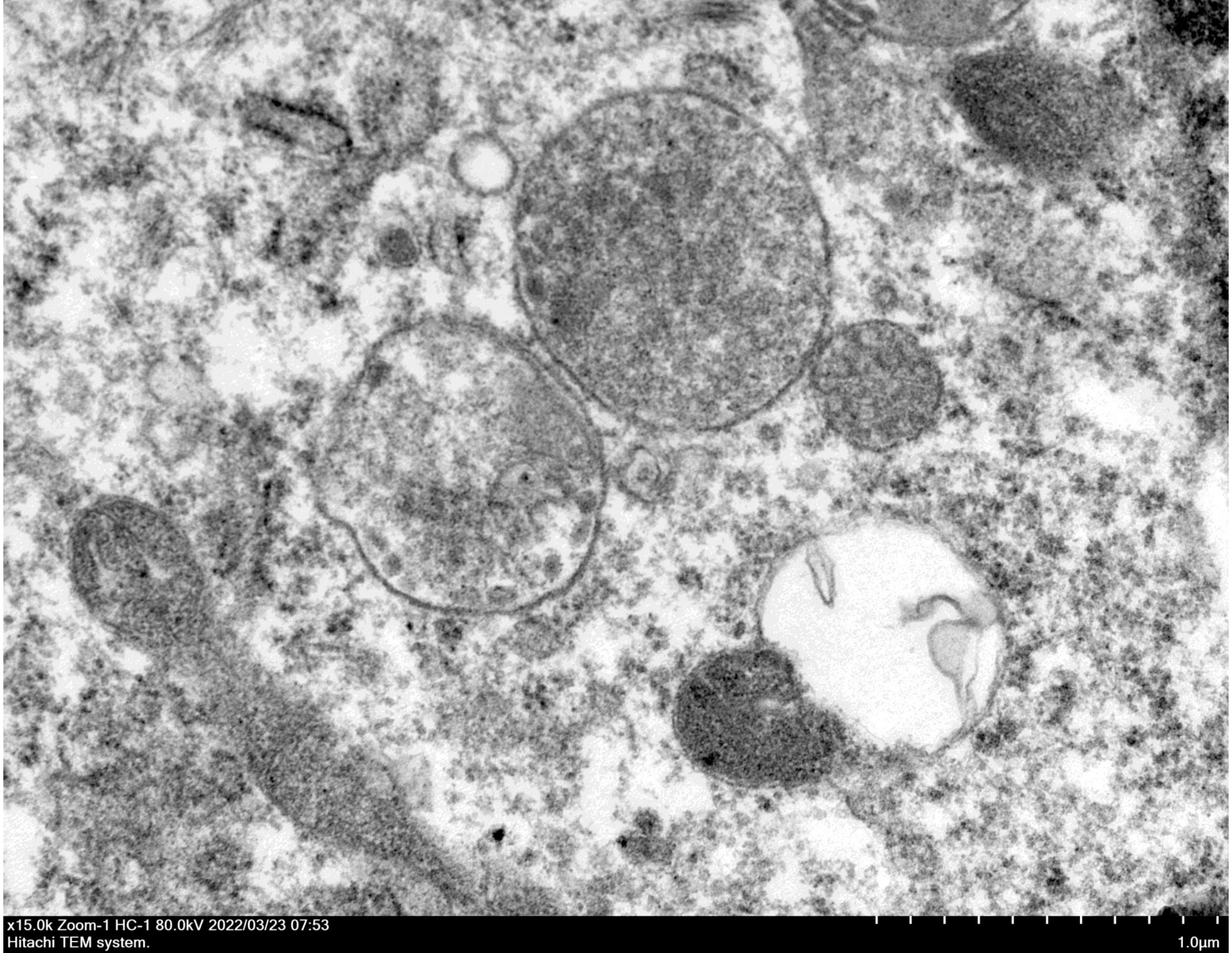
x15.0k Zoom-1 HC-1 80.0kV 2022/03/23 07:53
Hitachi TEM system.
1.0μm

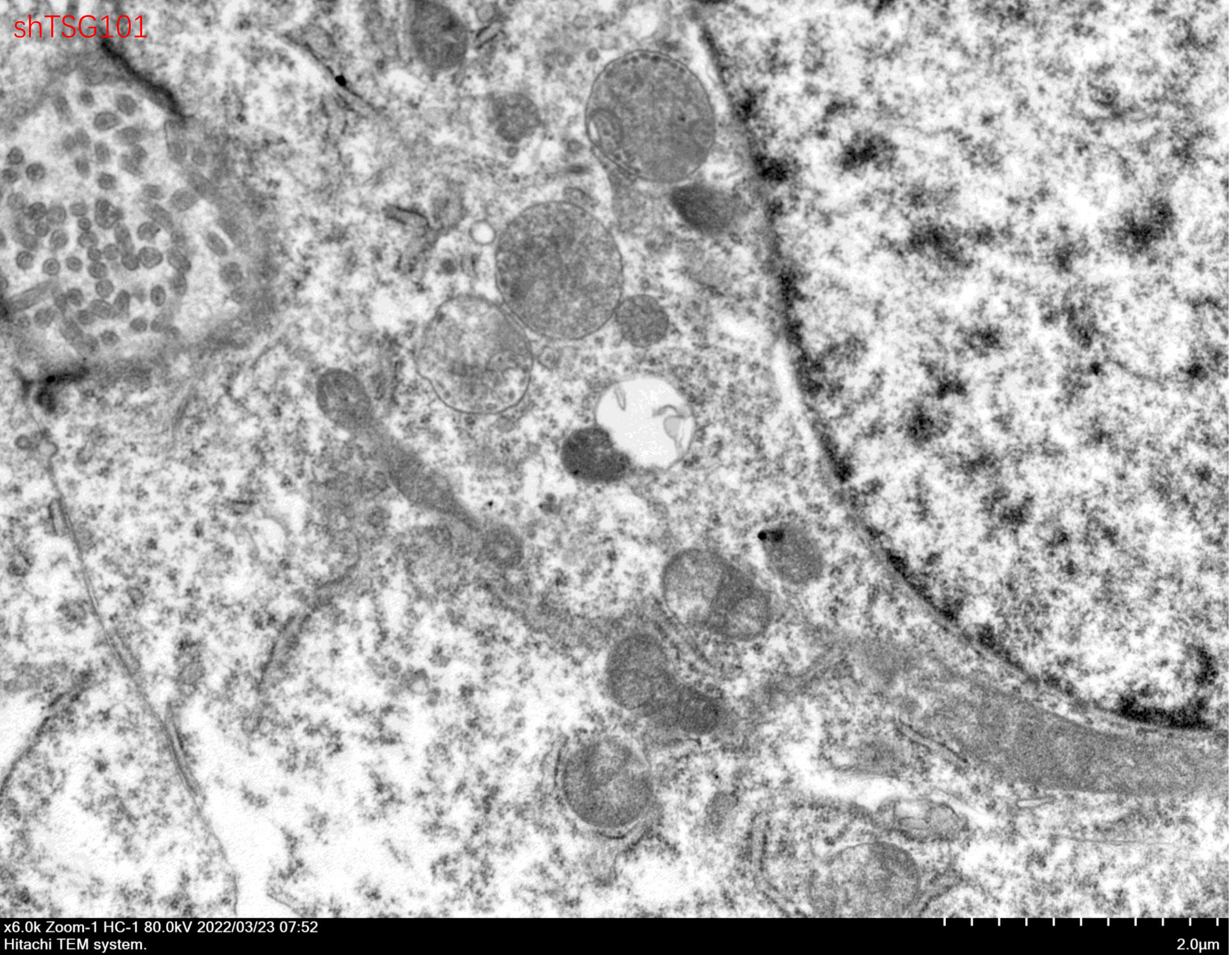
shTSG101
x6.0k Zoom-1 HC-1 80.0kV 2022/03/23 07:52
Hitachi TEM system.
2.0μm

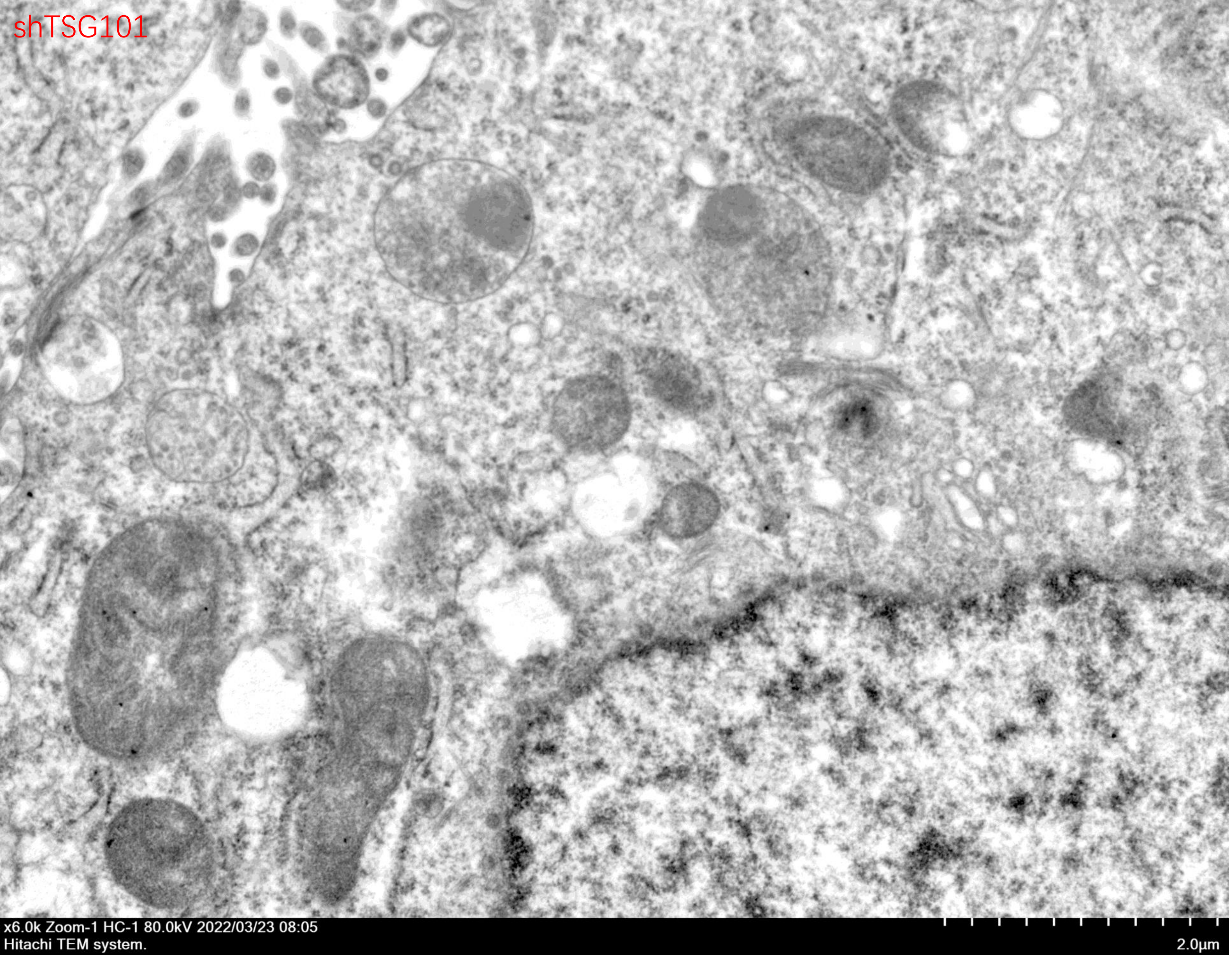
shTSG101
x6.0k Zoom-1 HC-1 80.0kV 2022/03/23 08:05
Hitachi TEM system.
2.0μm

shTSG101

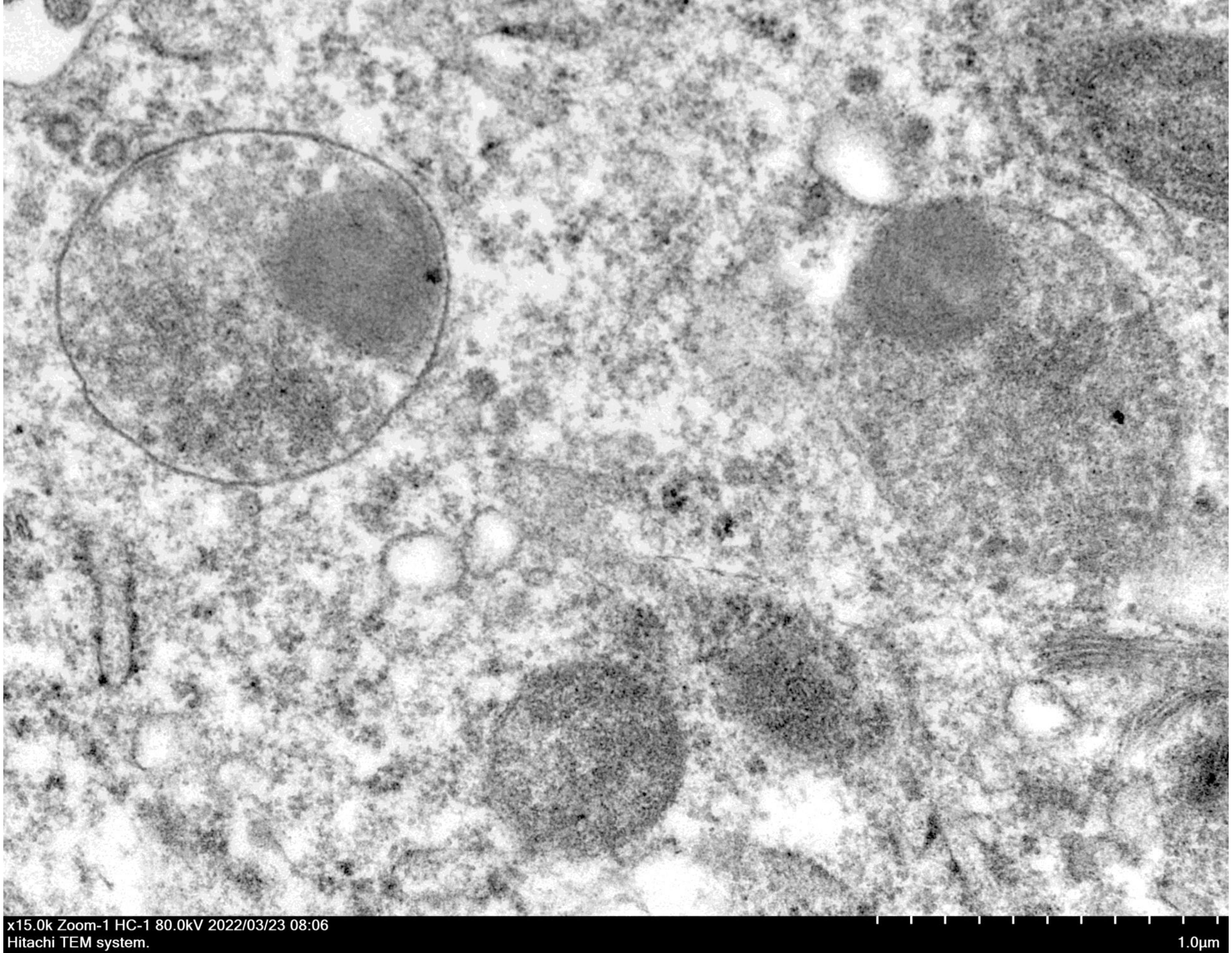
x15.0k Zoom-1 HC-1 80.0kV 2022/03/23 08:06
Hitachi TEM system.
1.0μm

Fig 2F

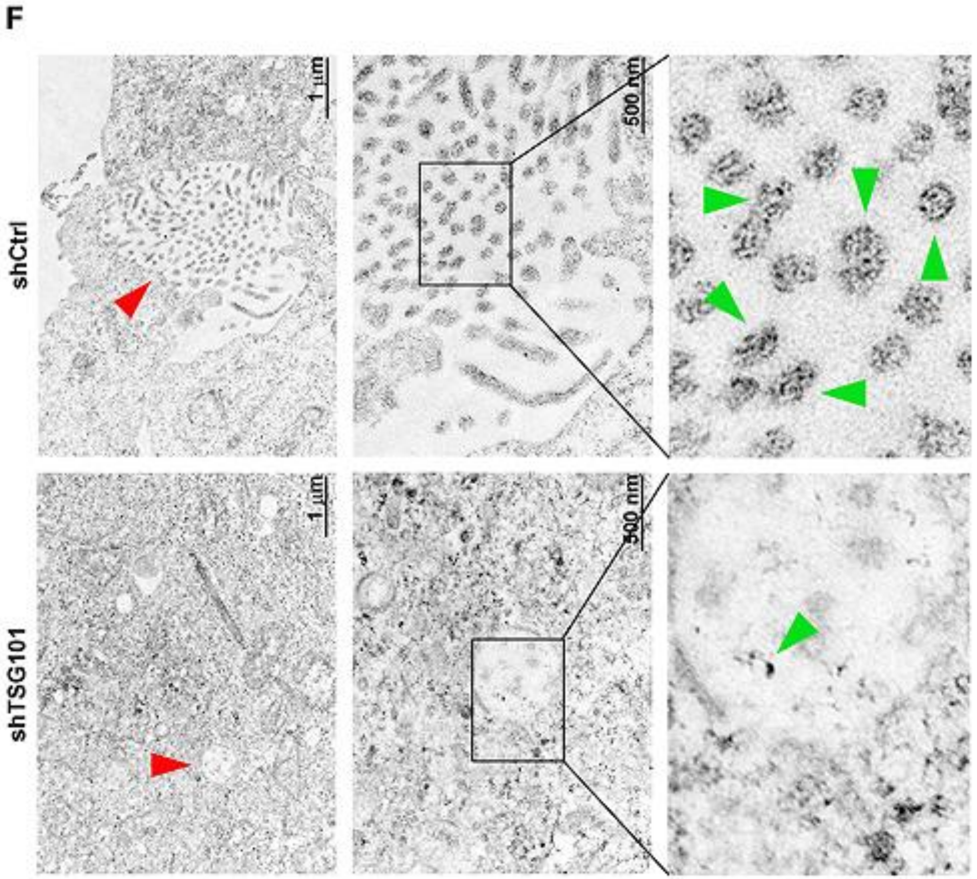

shCtrl

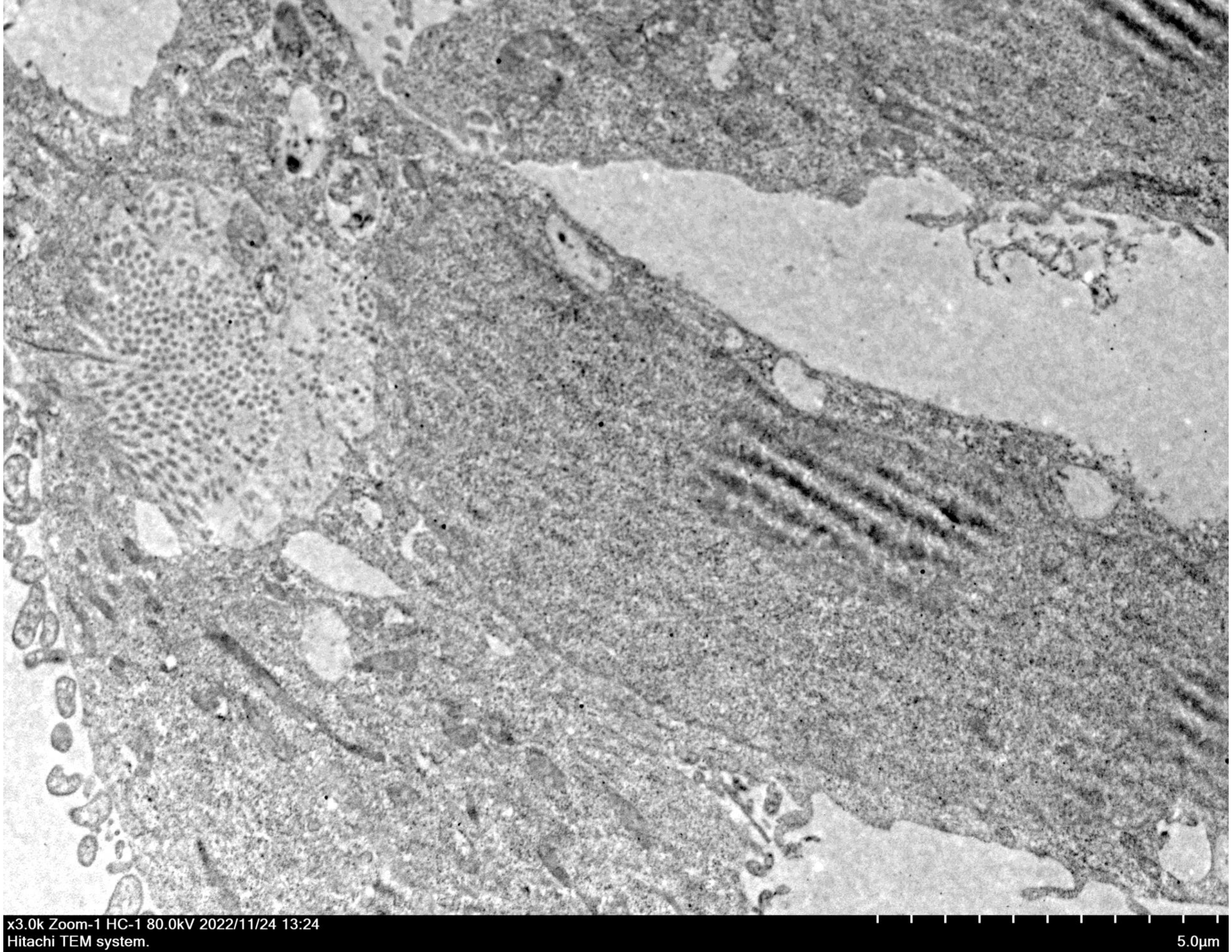

shCtrl

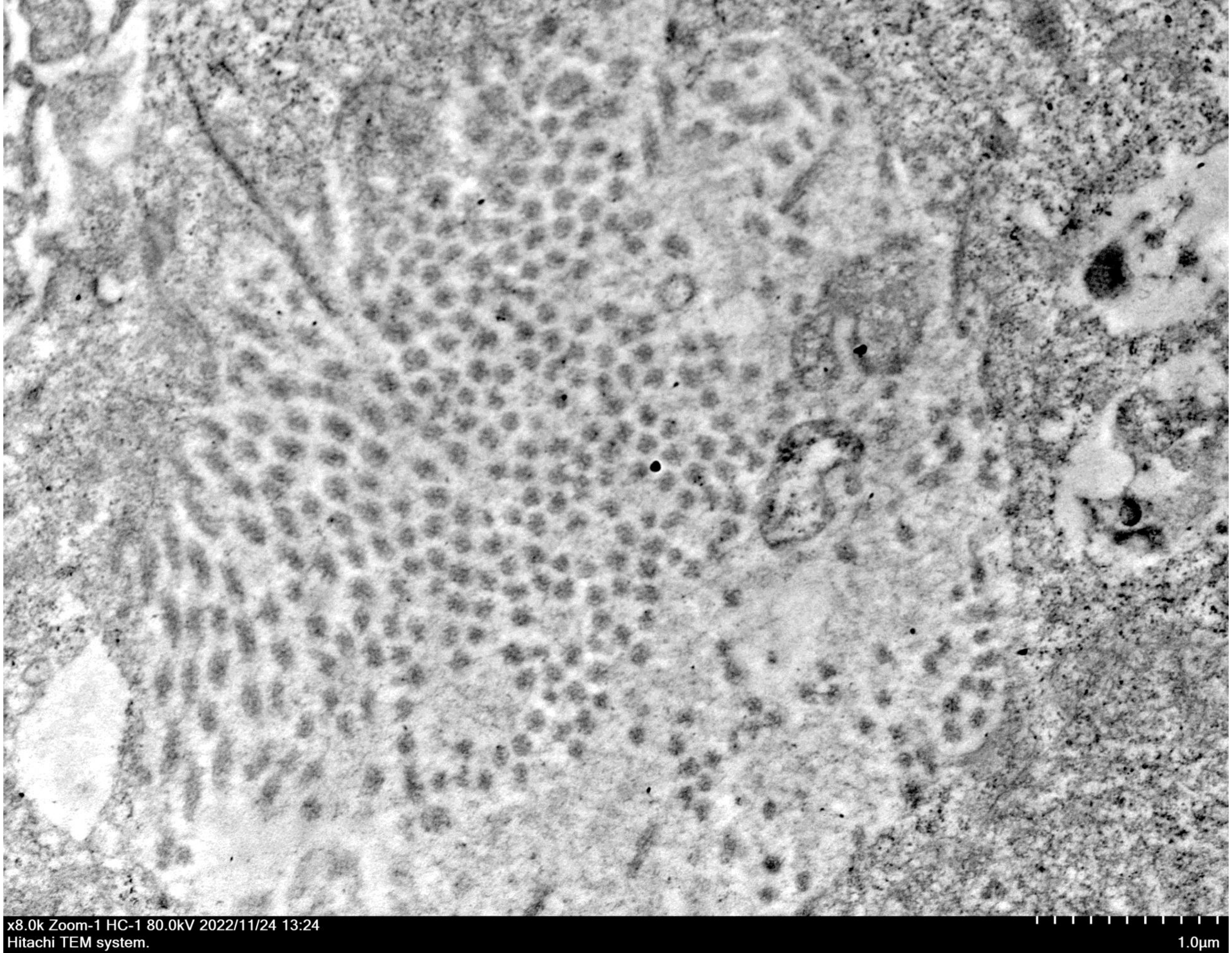

shCtrl

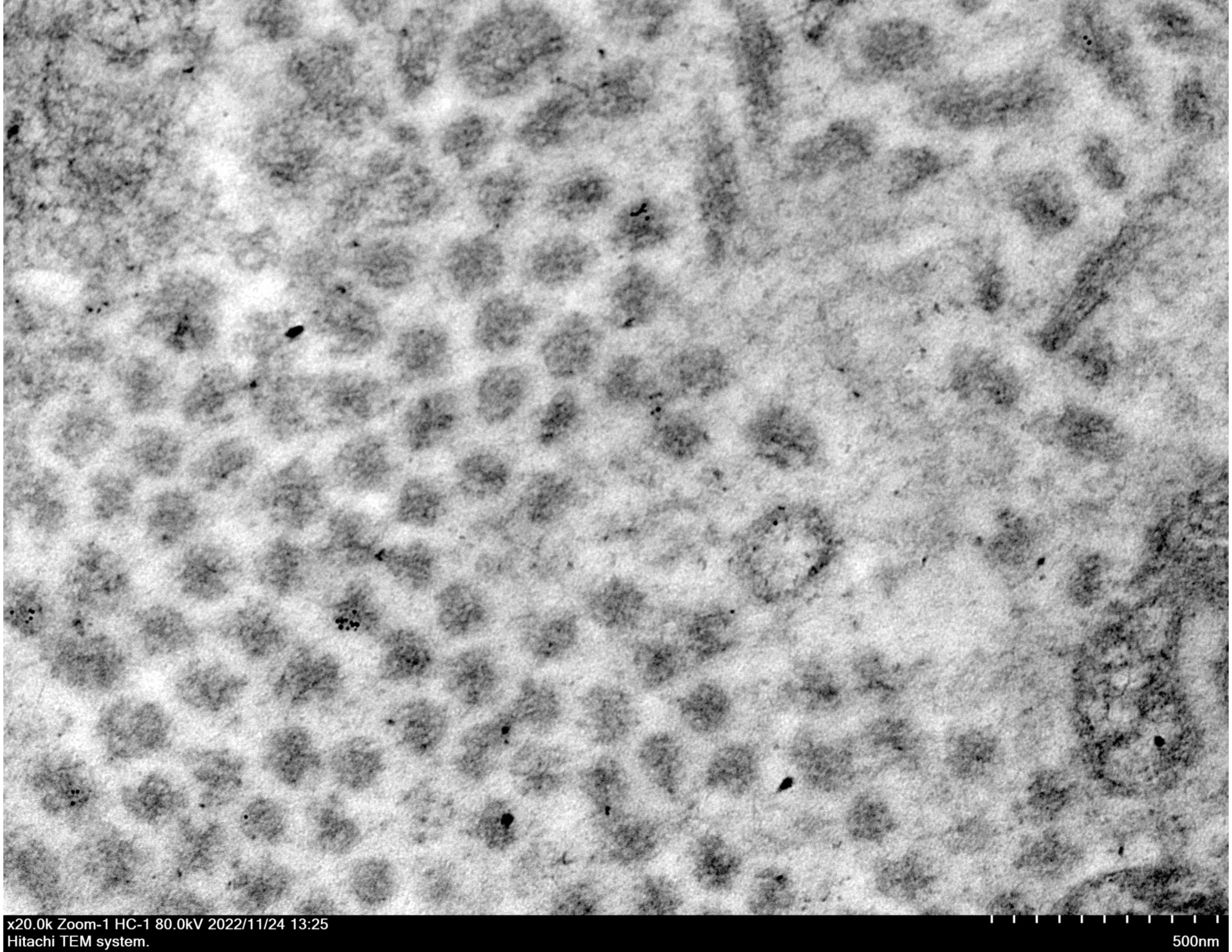
x20.0k Zoom-1 HC-1 80.0kV 2022/11/24 13:25
Hitachi TEM system.
500nm

shhTSG101

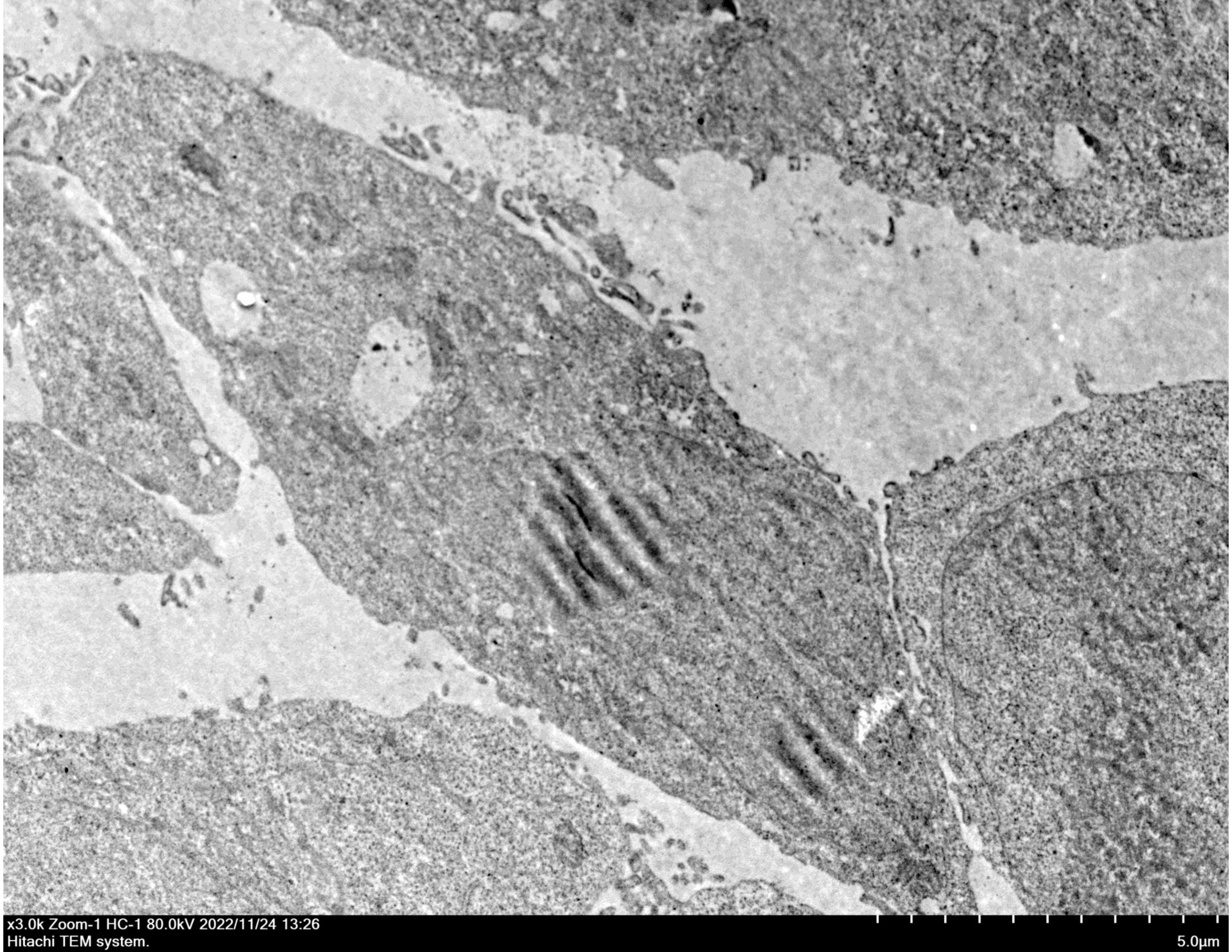

shTSG101

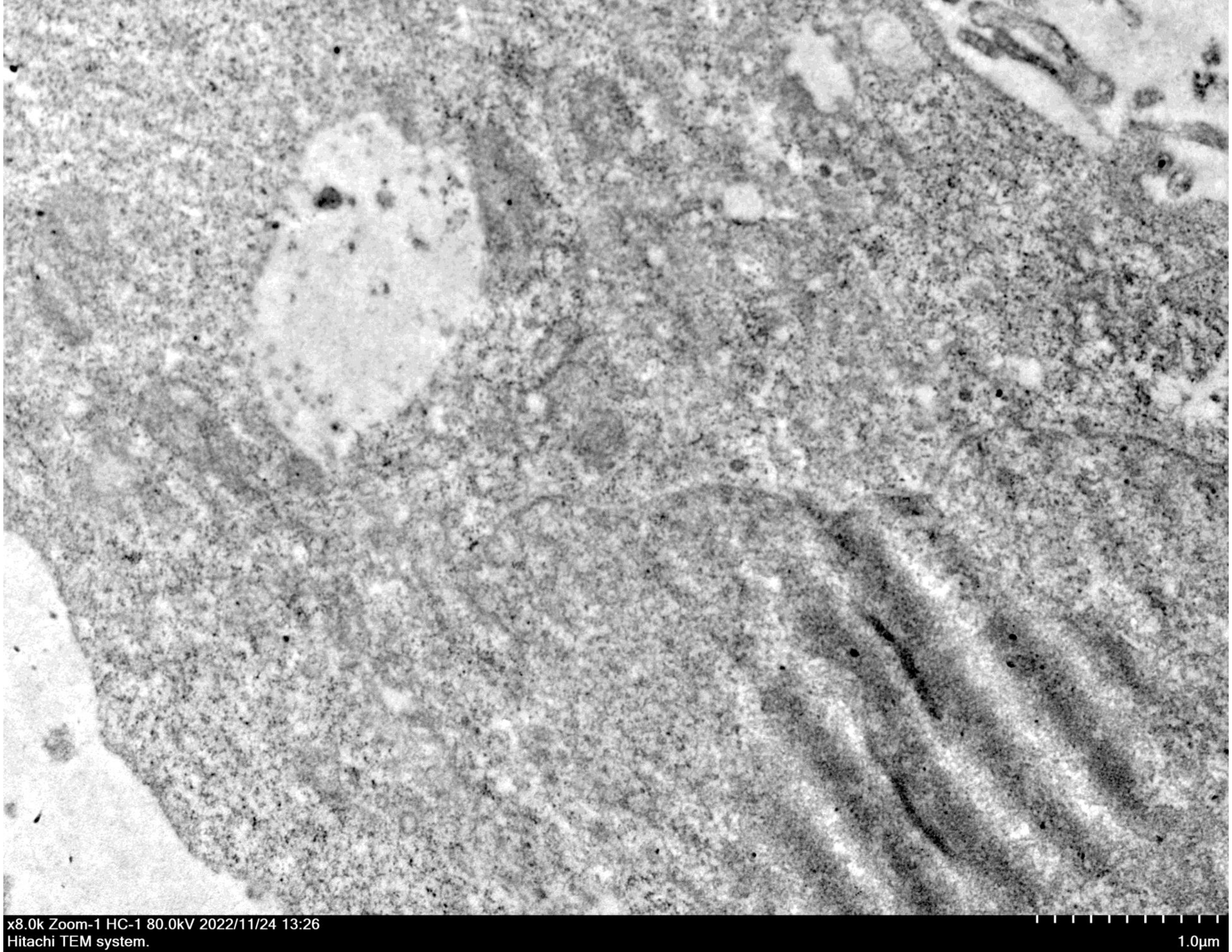

shTSG101

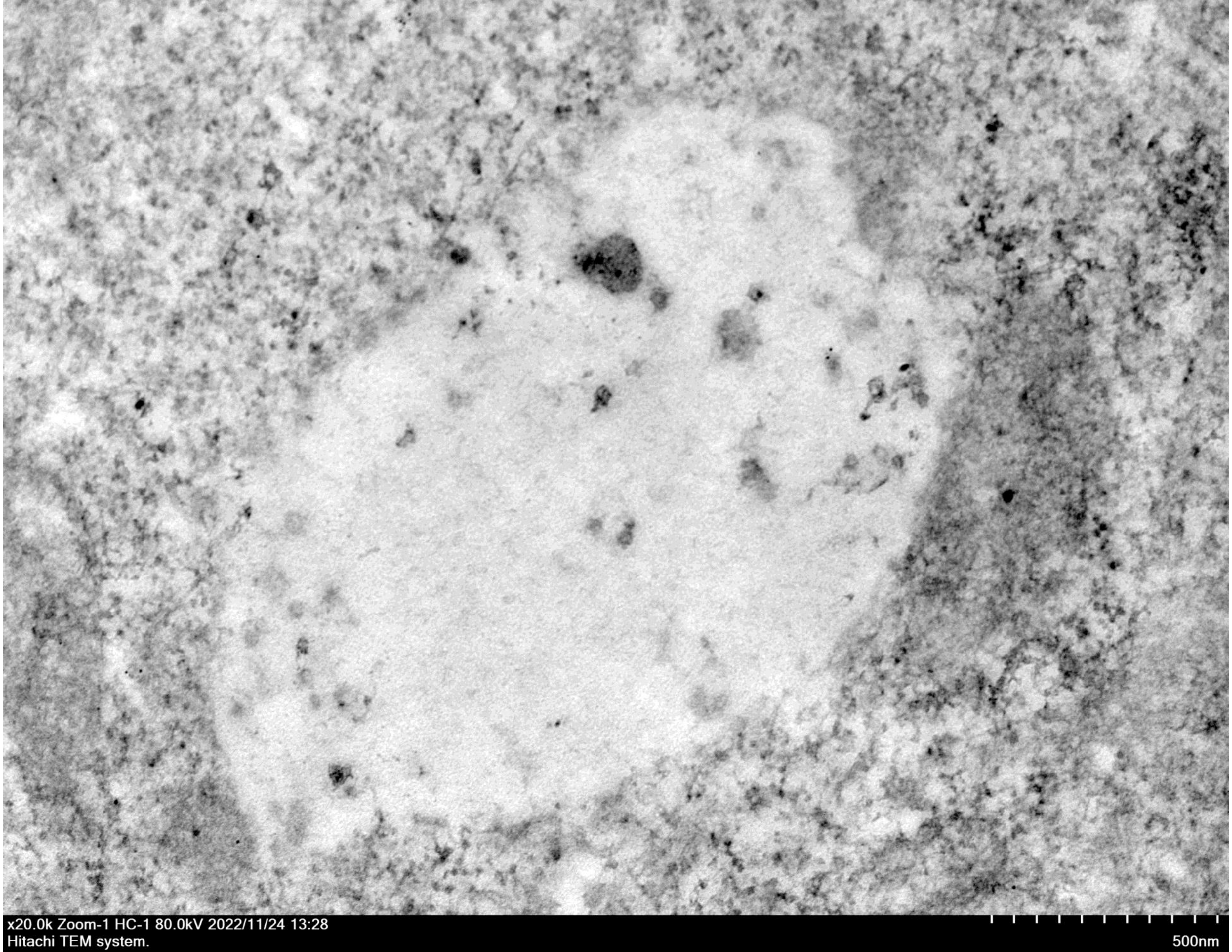
x20.0k Zoom-1 HC-1 80.0kV 2022/11/24 13:28
Hitachi TEM system.
500nm

Fig 5H

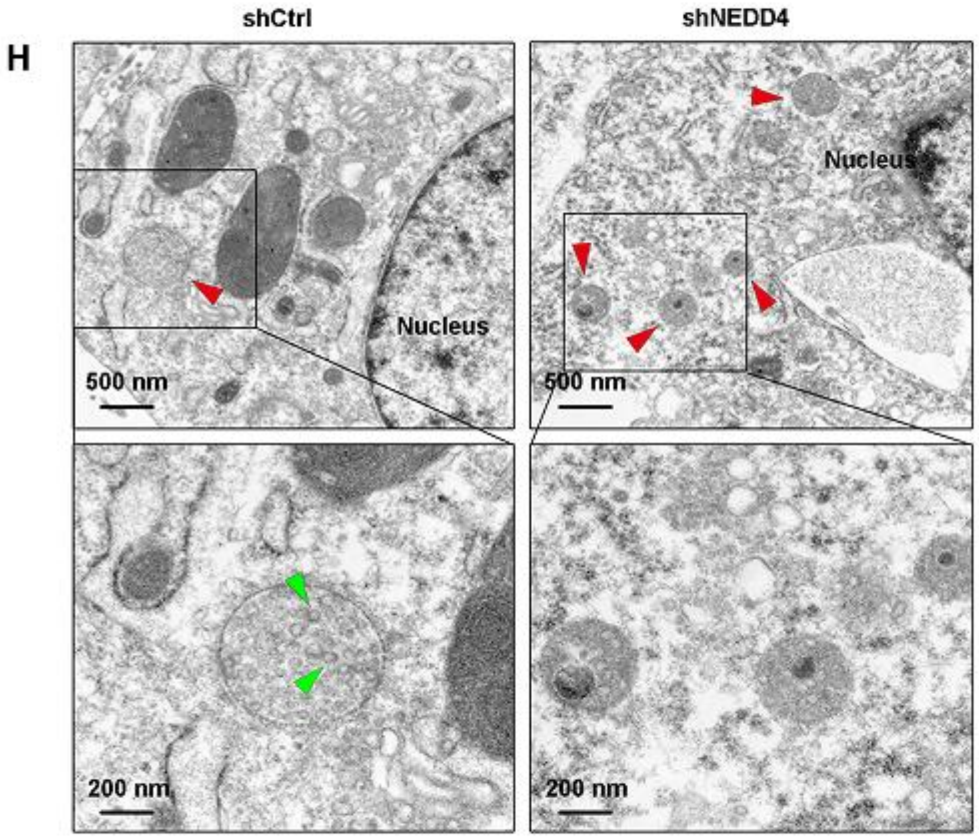

shCtrl

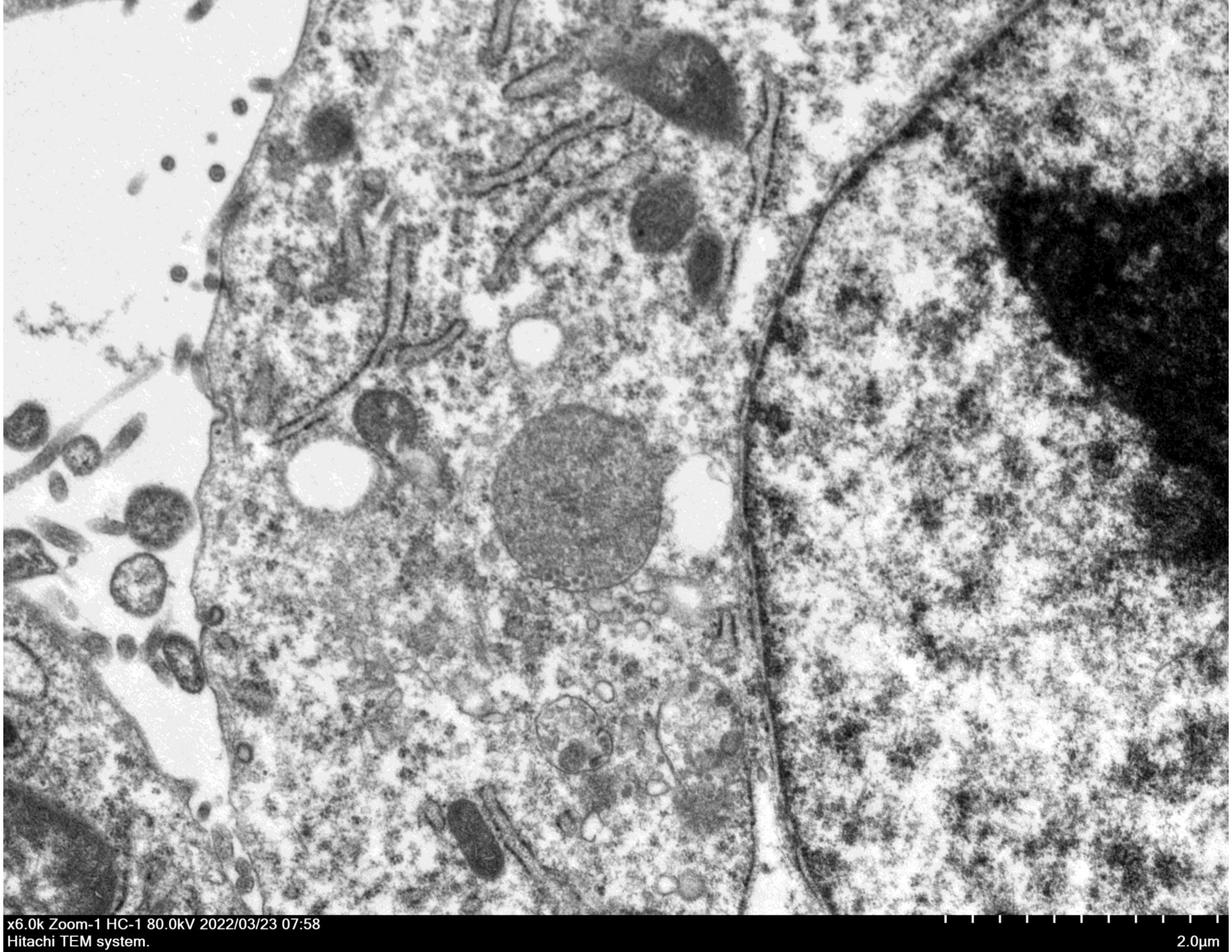

shCtrl

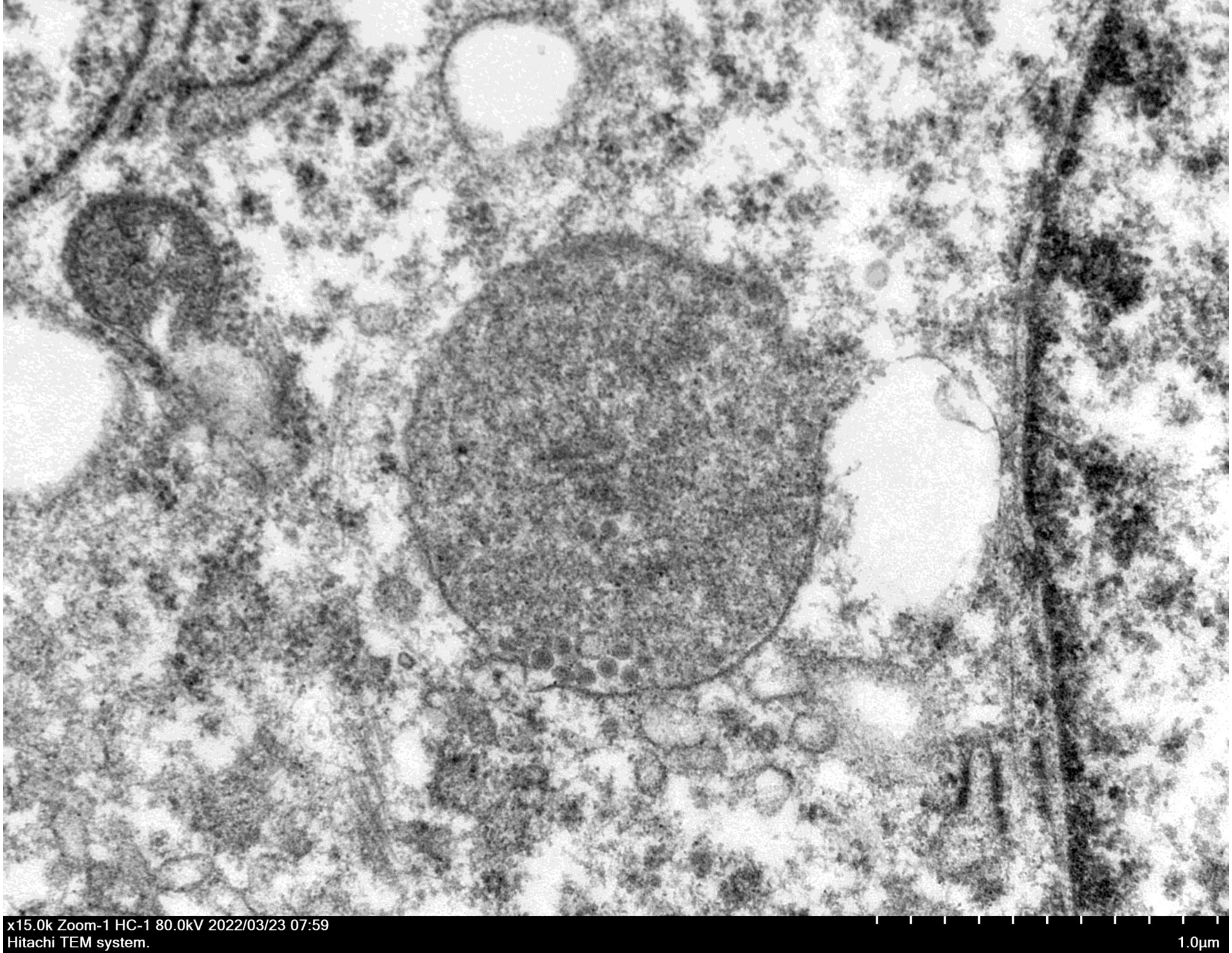
x15.0k Zoom-1 HC-1 80.0kV 2022/03/23 07:59
Hitachi TEM system.
1.0μm

shCtrl

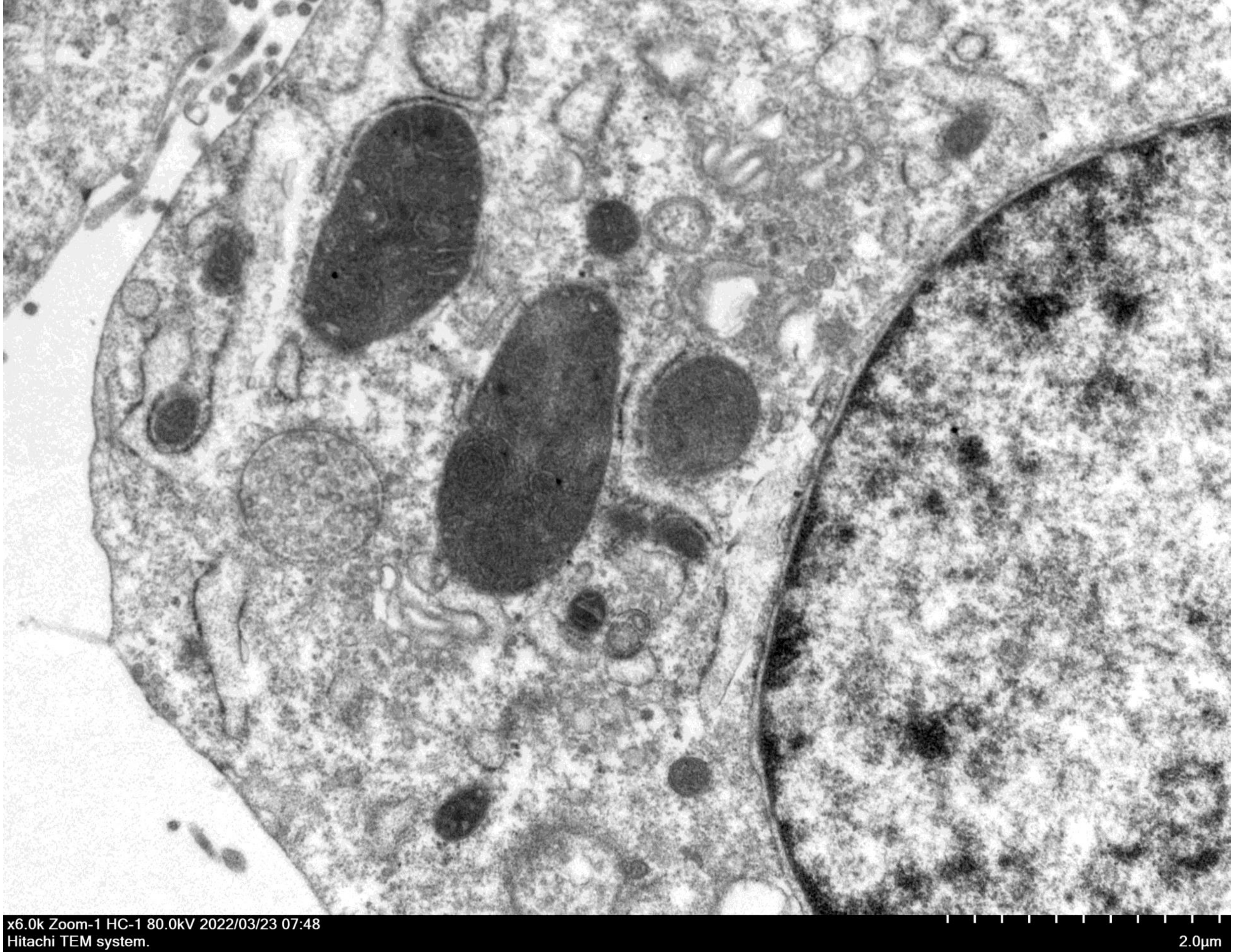

shCtrl

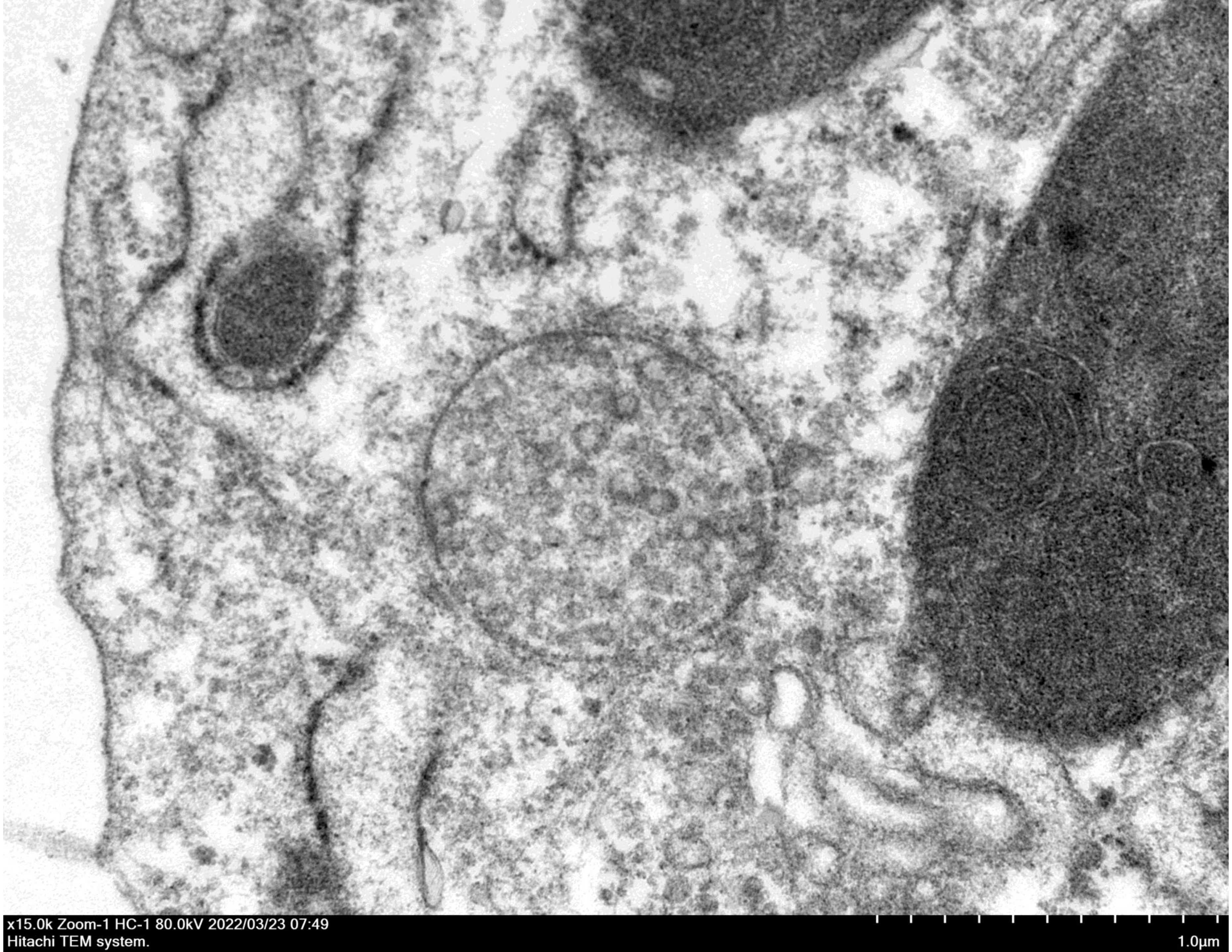
x15.0k Zoom-1 HC-1 80.0kV 2022/03/23 07:49
Hitachi TEM system.
1.0µm

shCtrl

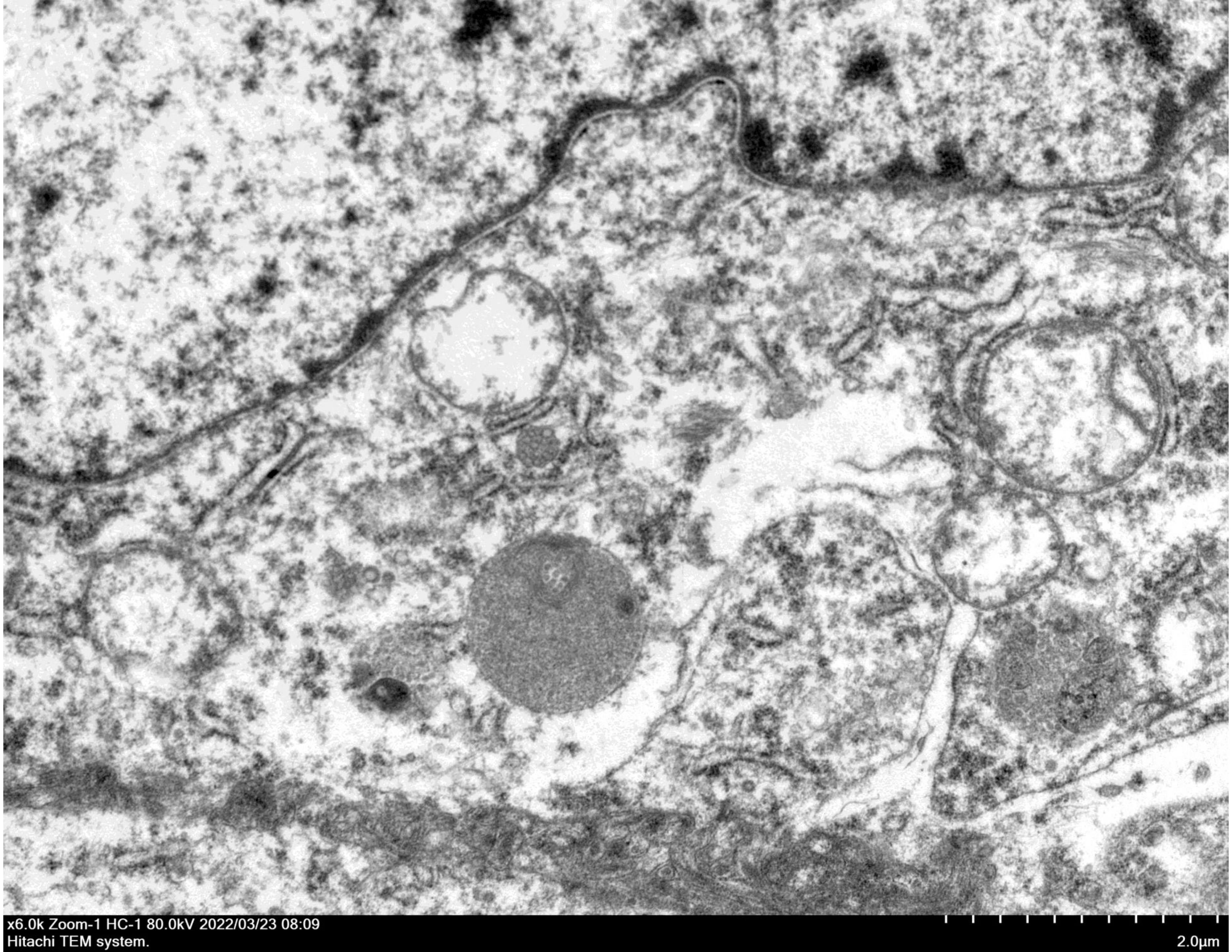

shCtrl

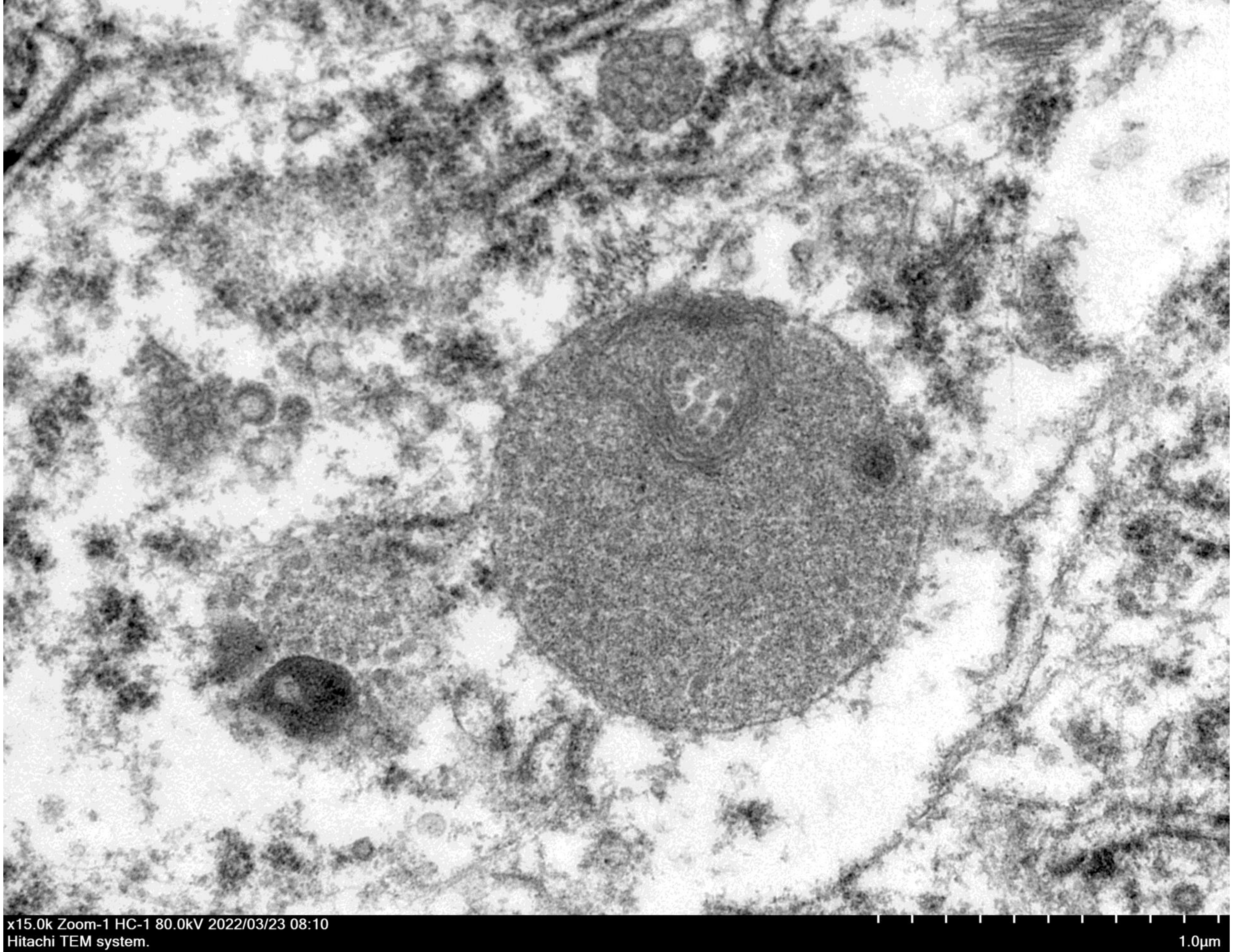
x15.0k Zoom-1 HC-1 80.0kV 2022/03/23 08:10
Hitachi TEM system.
1.0μm

shNEDD4

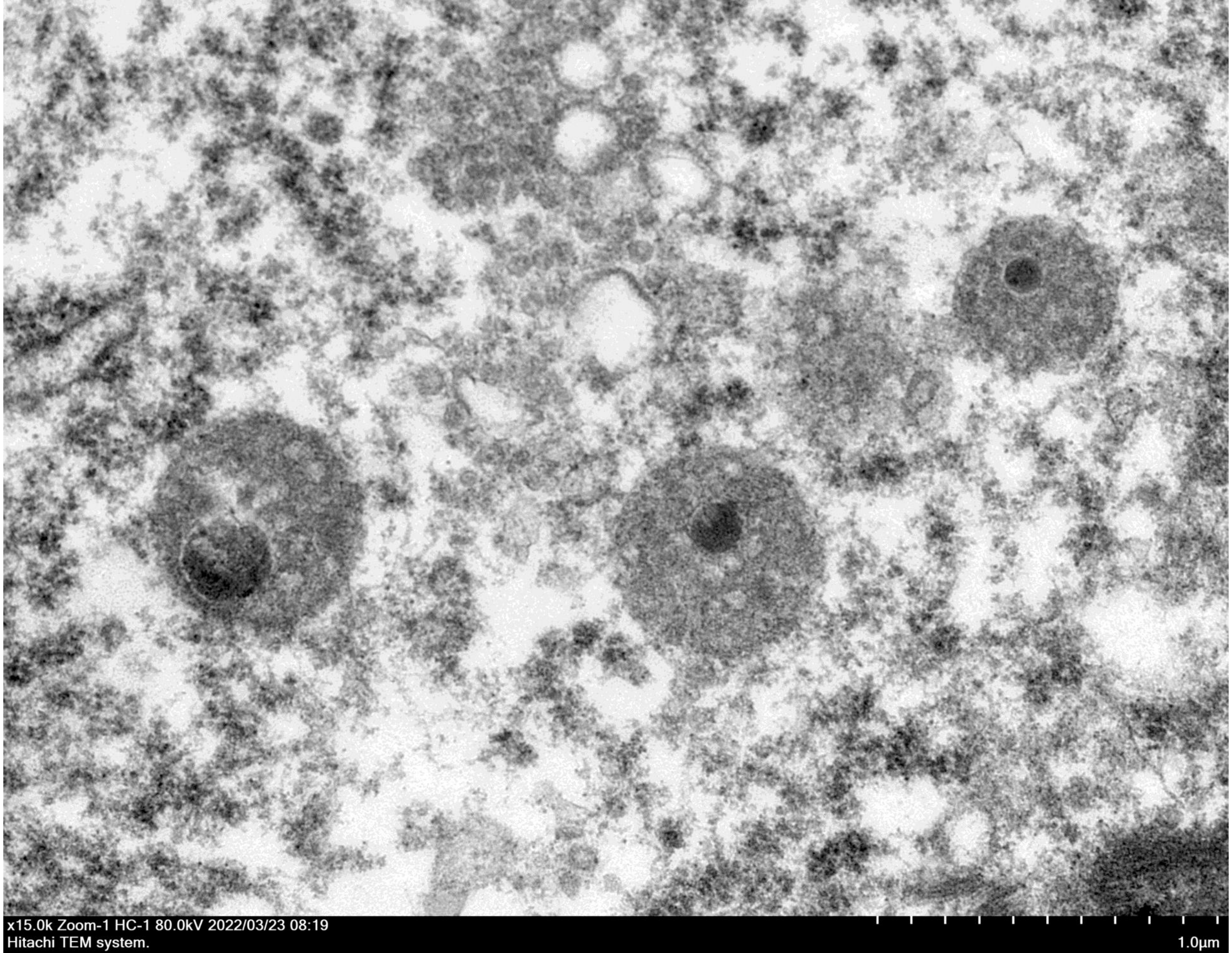
x15.0k Zoom-1 HC-1 80.0kV 2022/03/23 08:19
Hitachi TEM system.
1.0μm

shNEDD4

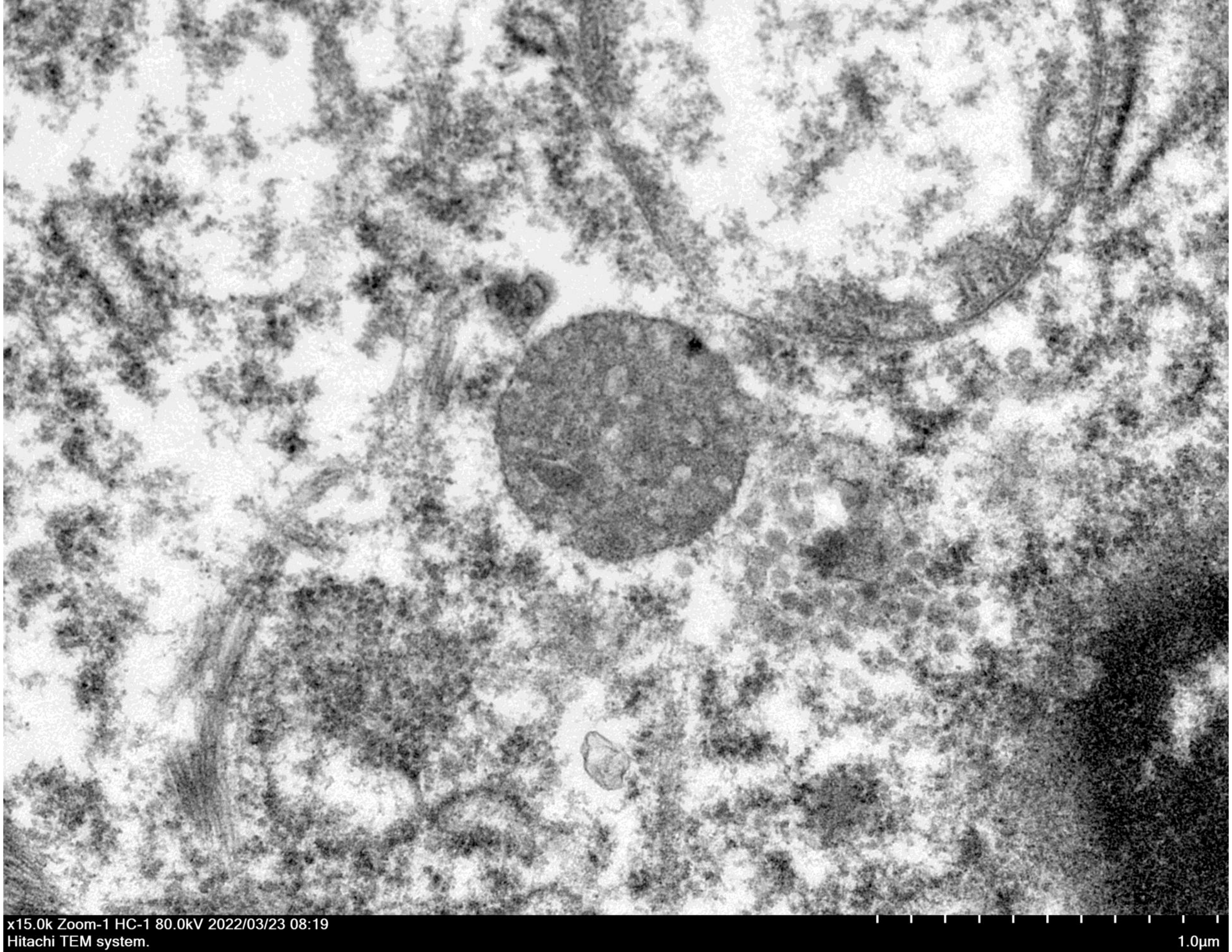
x15.0k Zoom-1 HC-1 80.0kV 2022/03/23 08:19
Hitachi TEM system.
1.0μm

shNEDD4

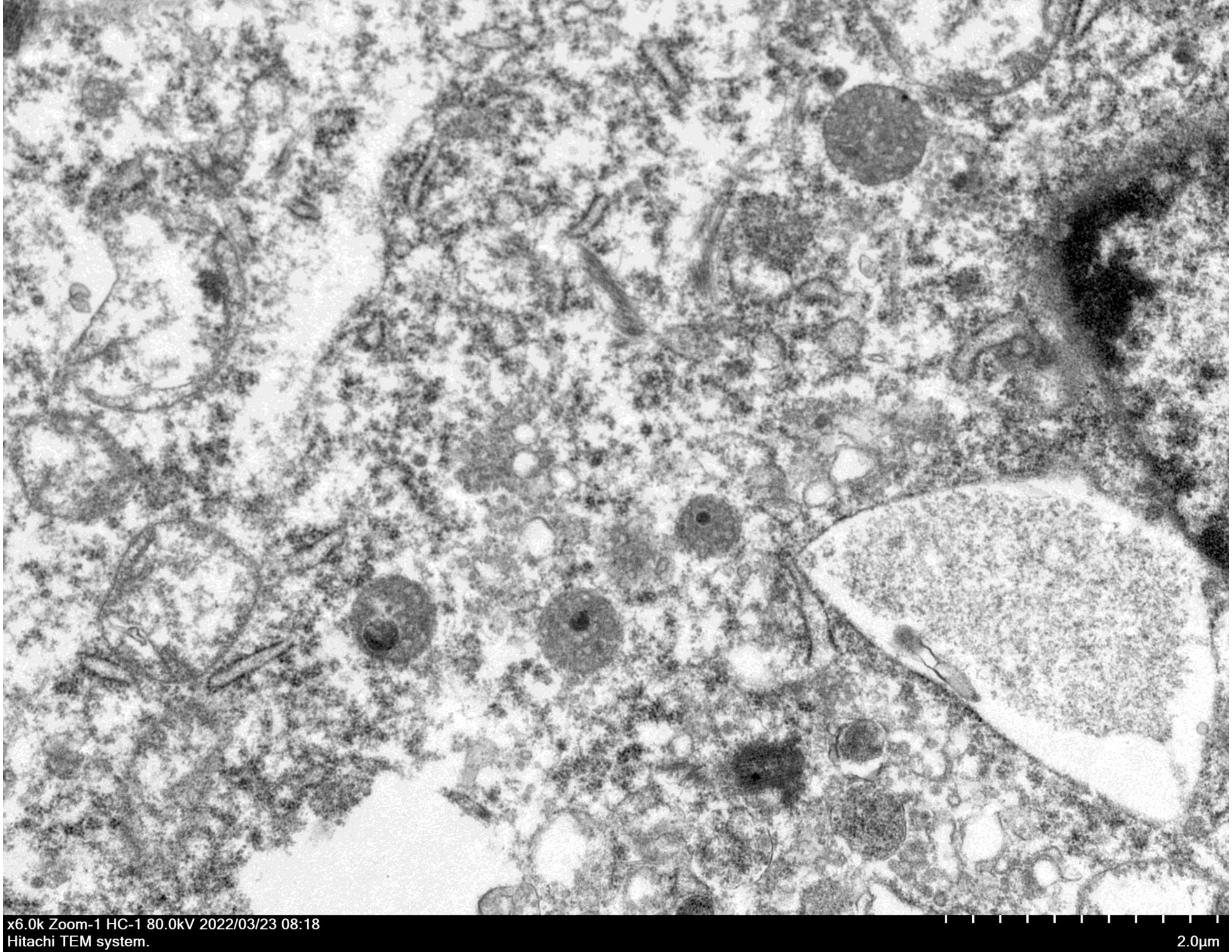

shNEDD4

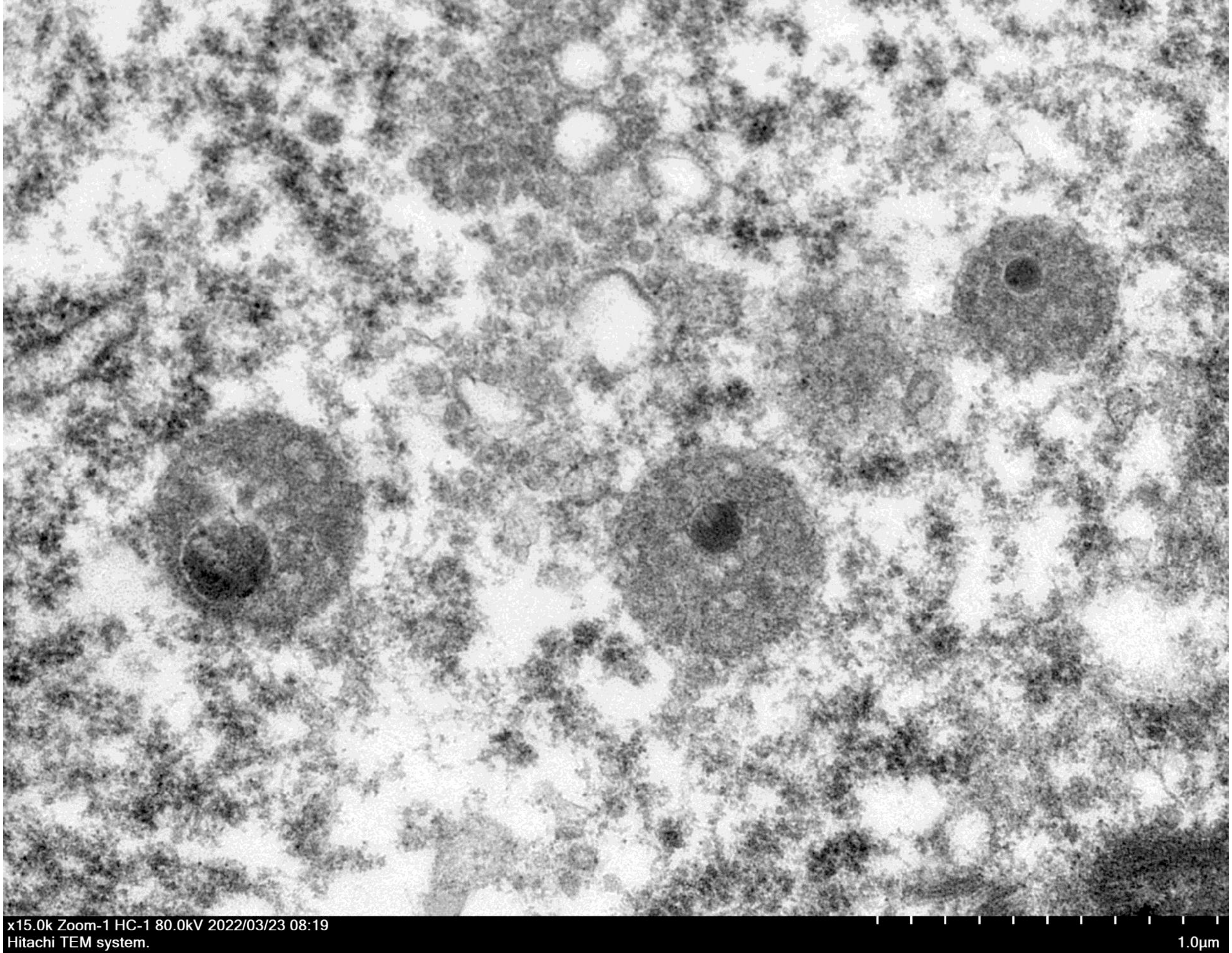
x15.0k Zoom-1 HC-1 80.0kV 2022/03/23 08:19
Hitachi TEM system.
1.0μm

shNEDD4

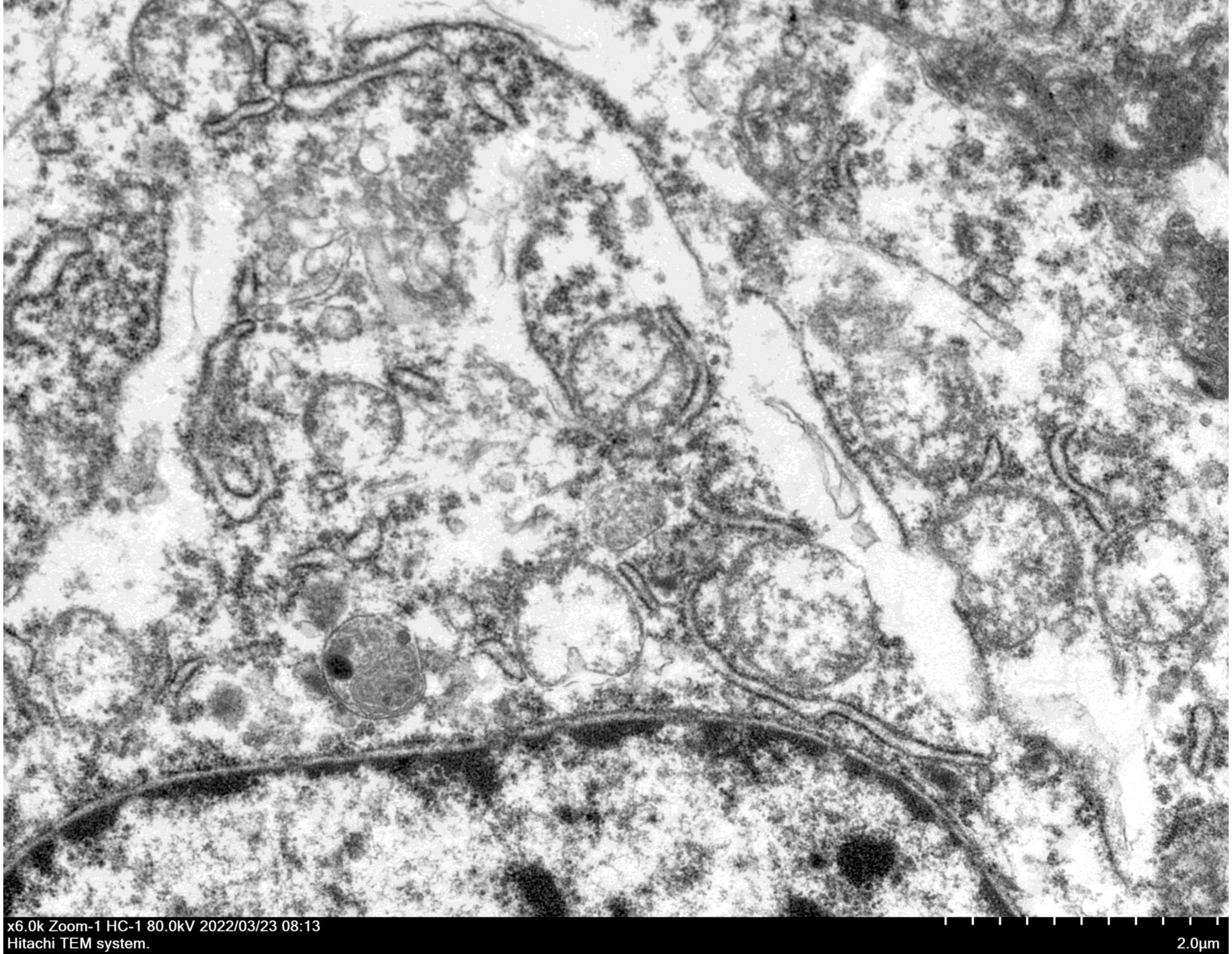

shNEDD4

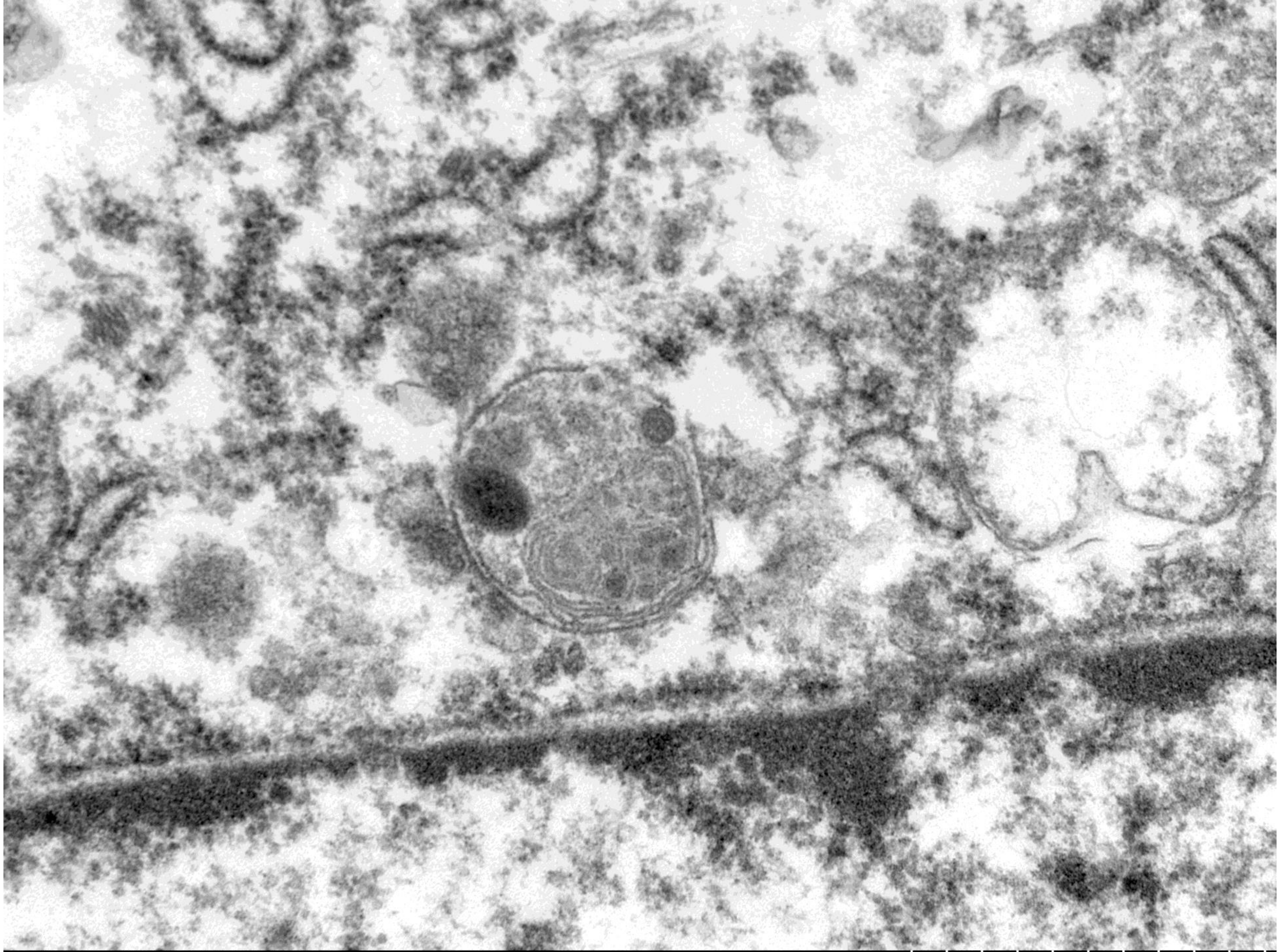

x15.0k Zoom-1 HC-1 80.0kV 2022/03/23 08:13
Hitachi TEM system.
1.0μm

S Fig3A

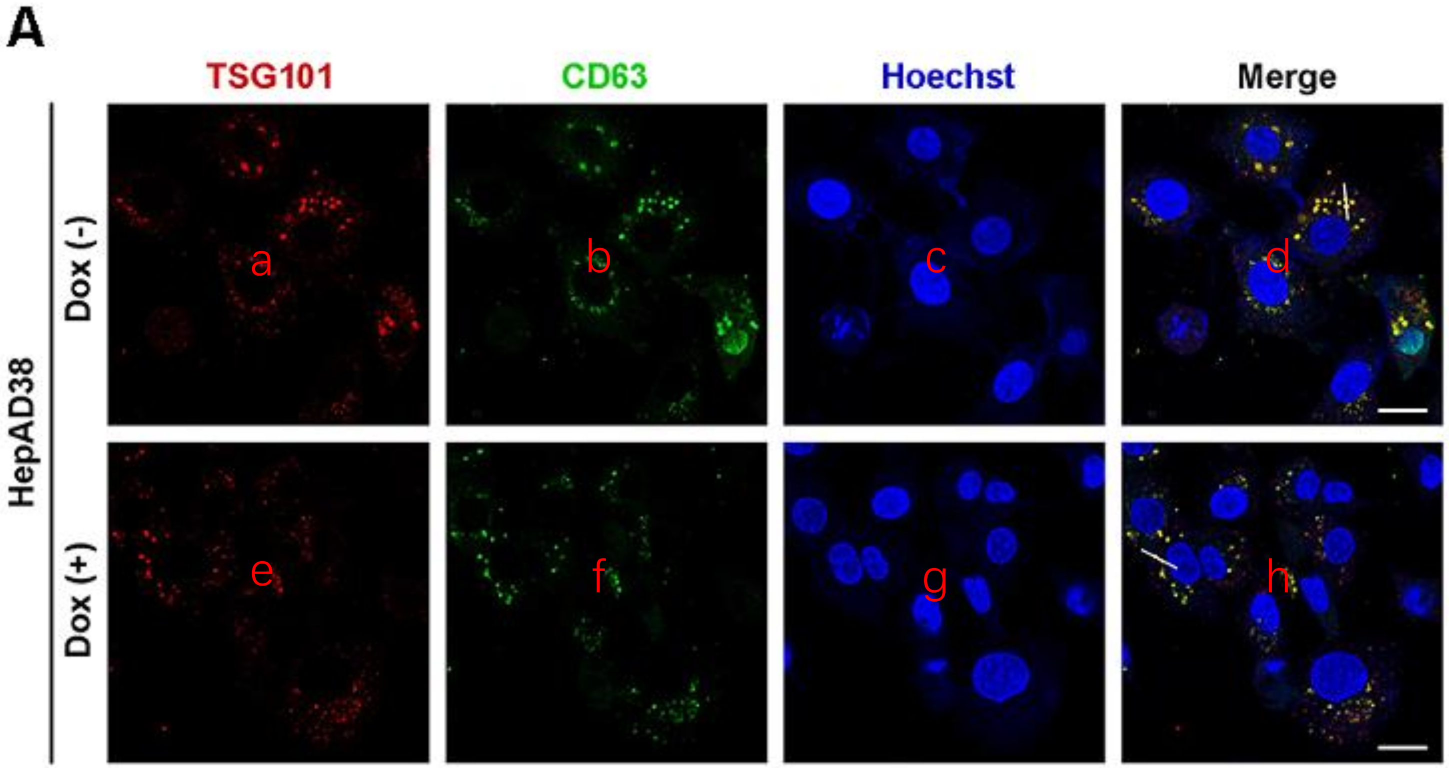

a

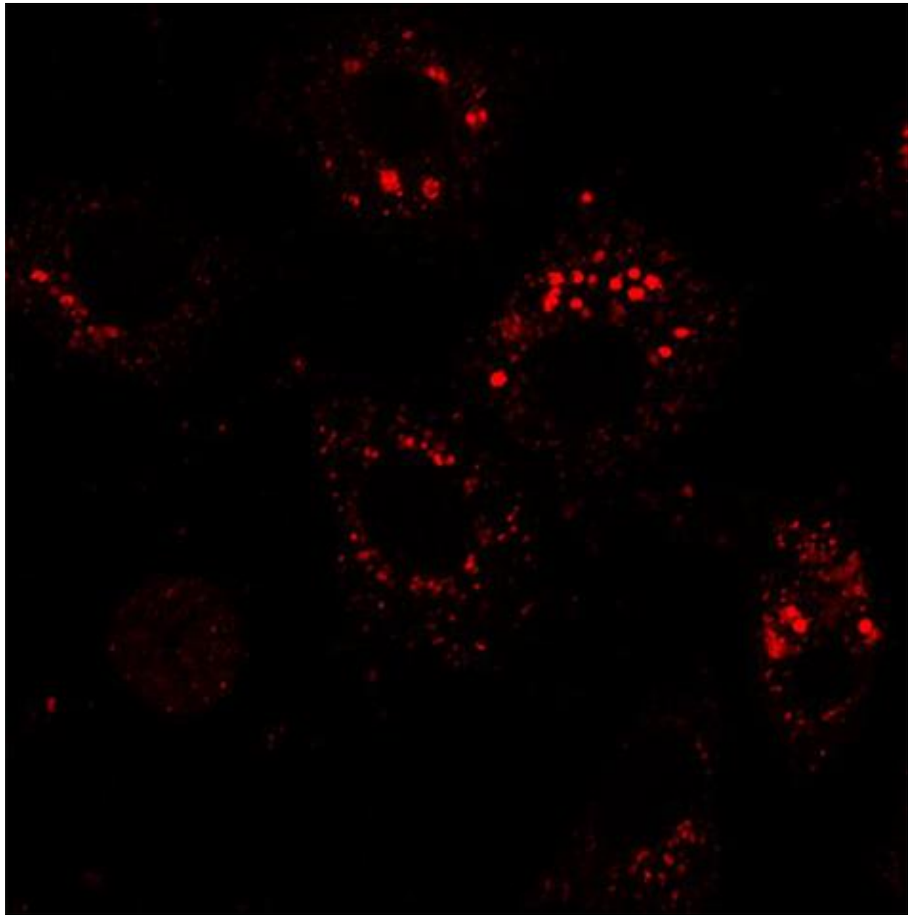

b

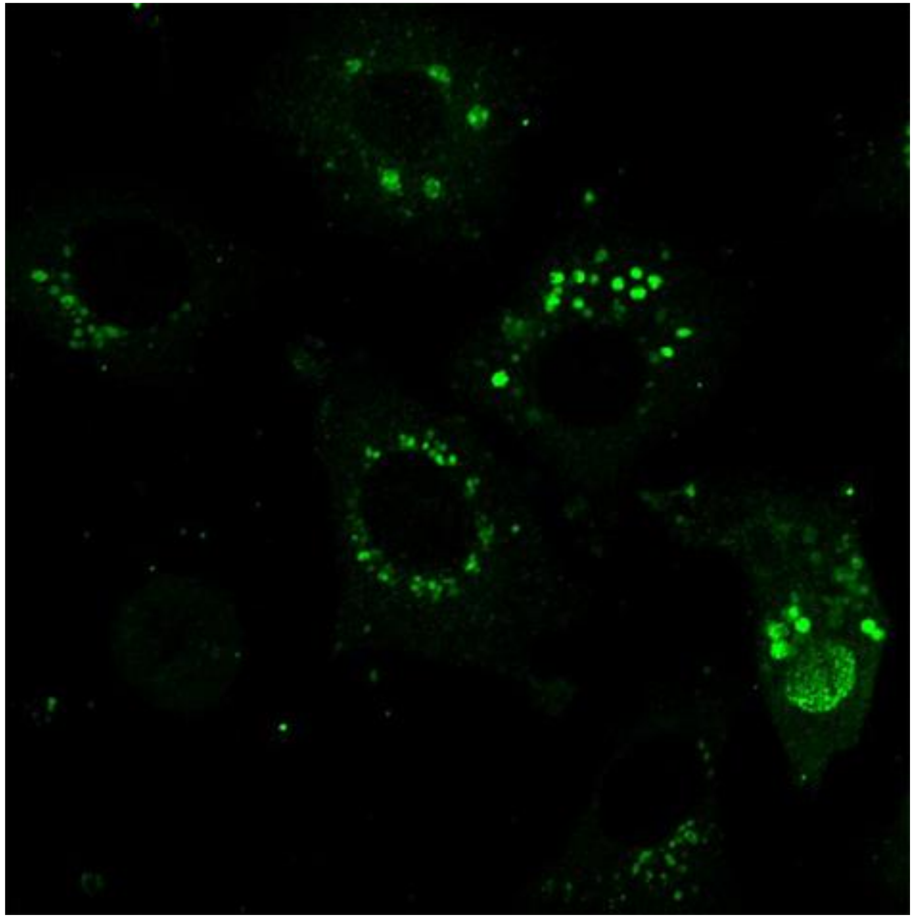

C

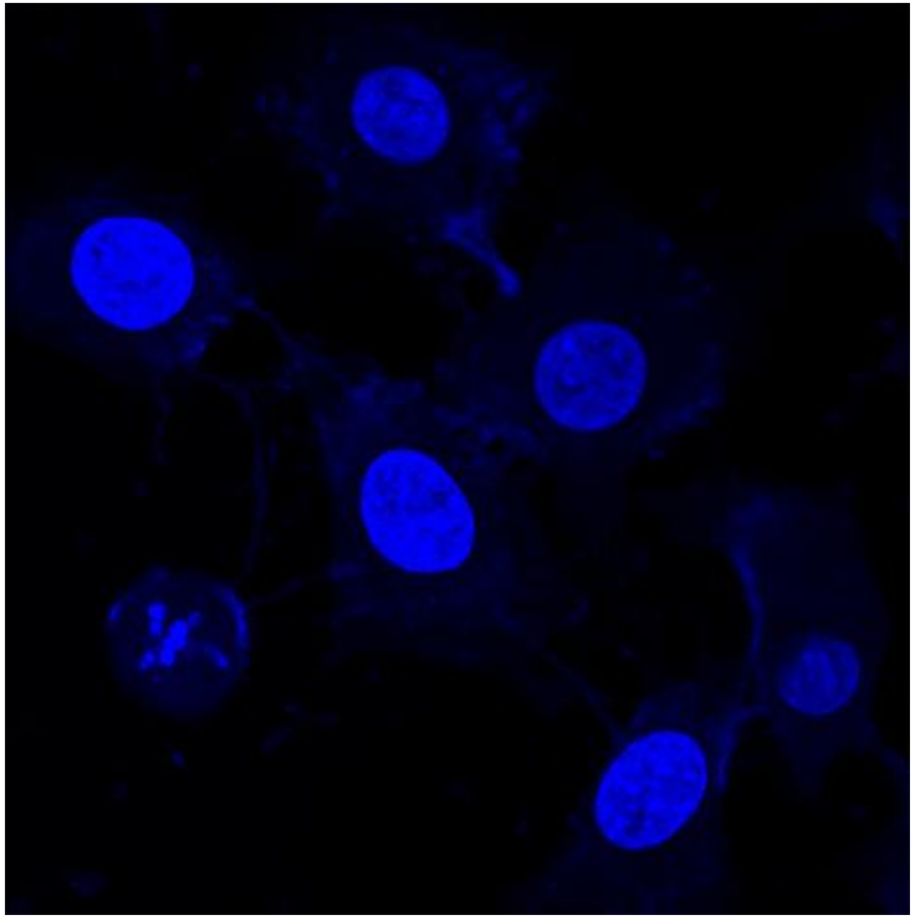

d

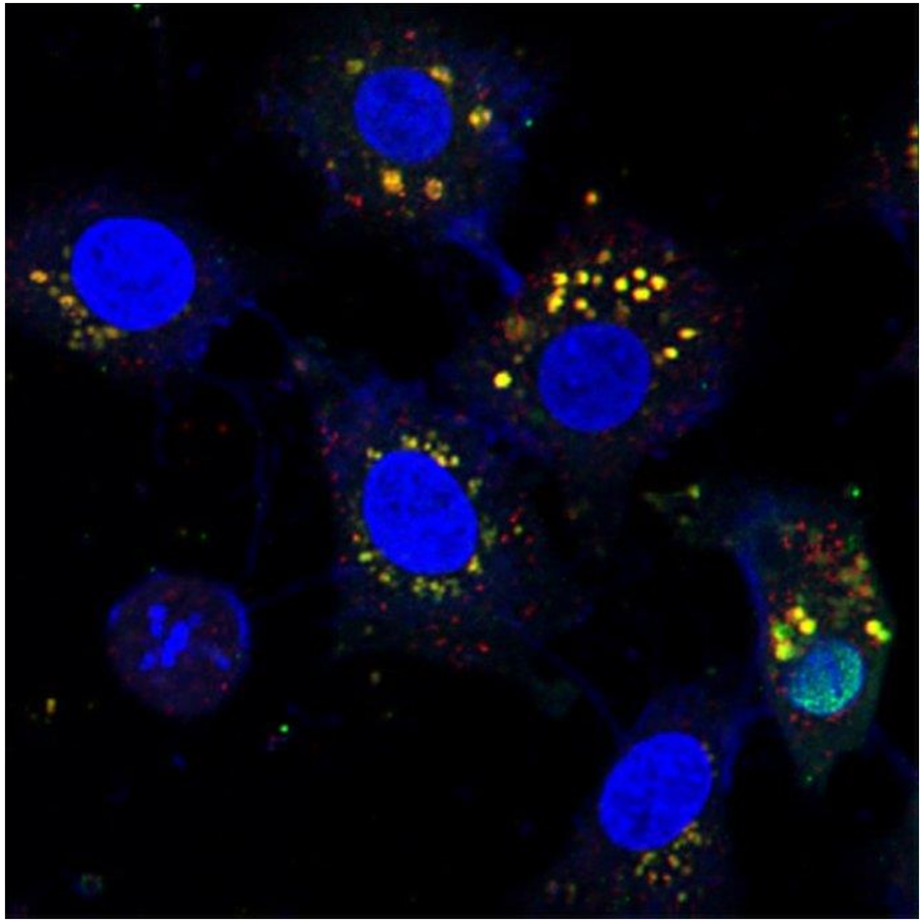

e

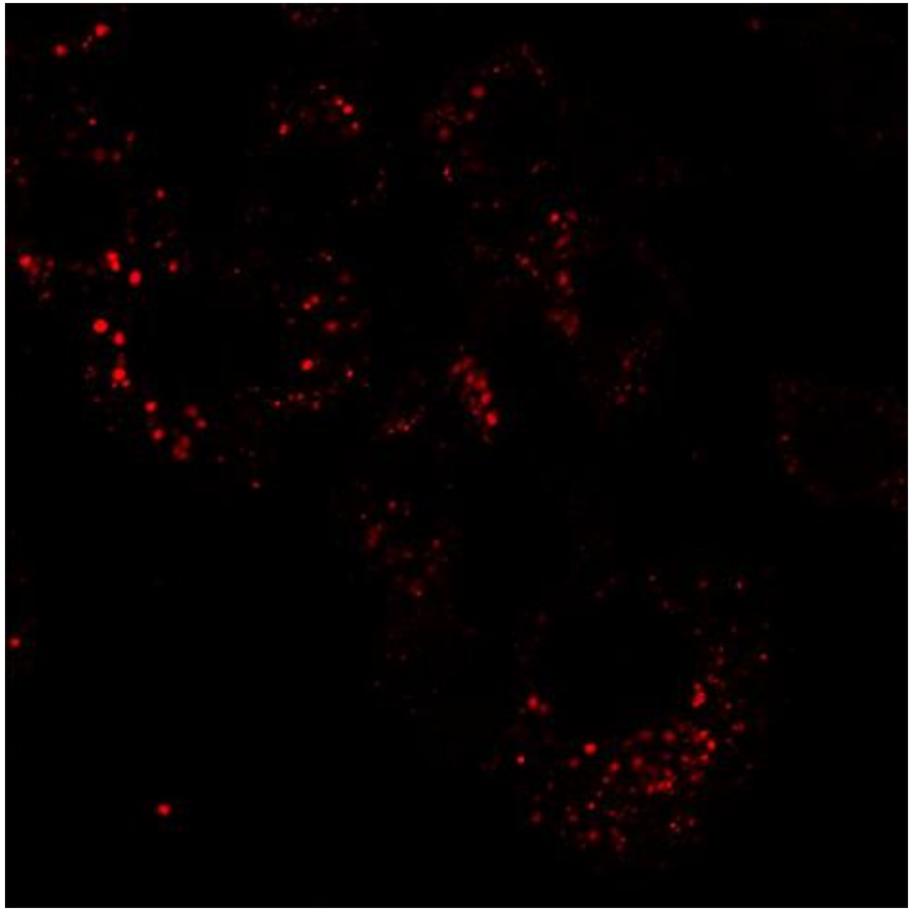

f

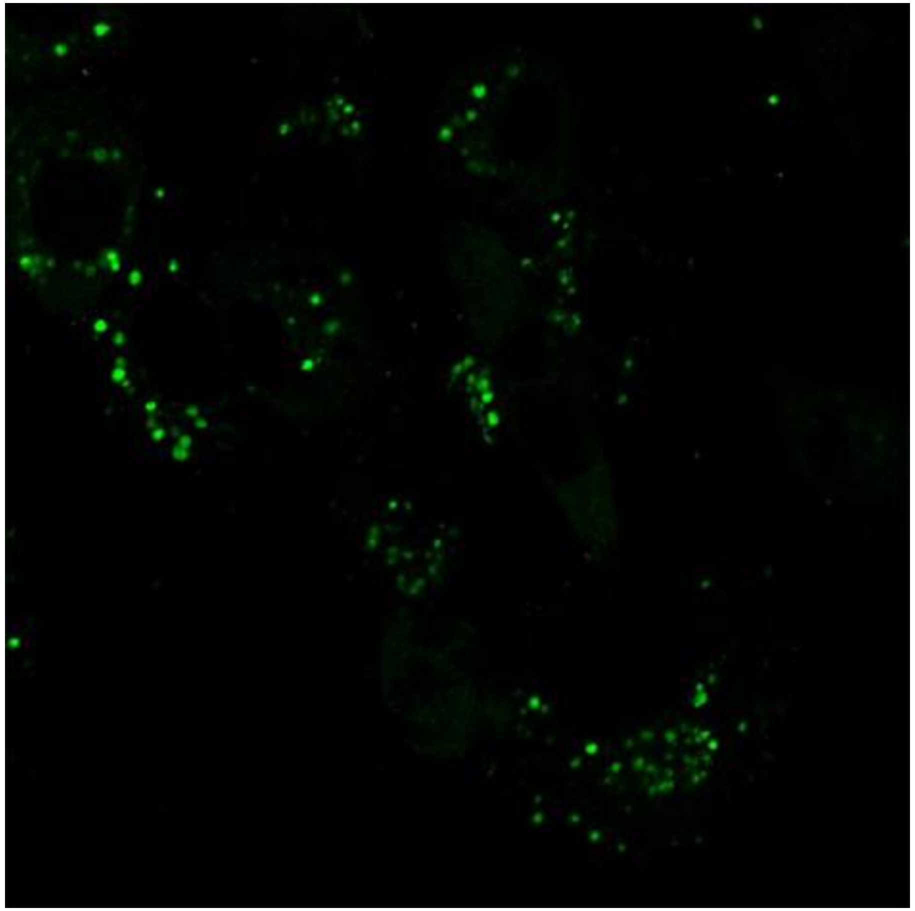

g

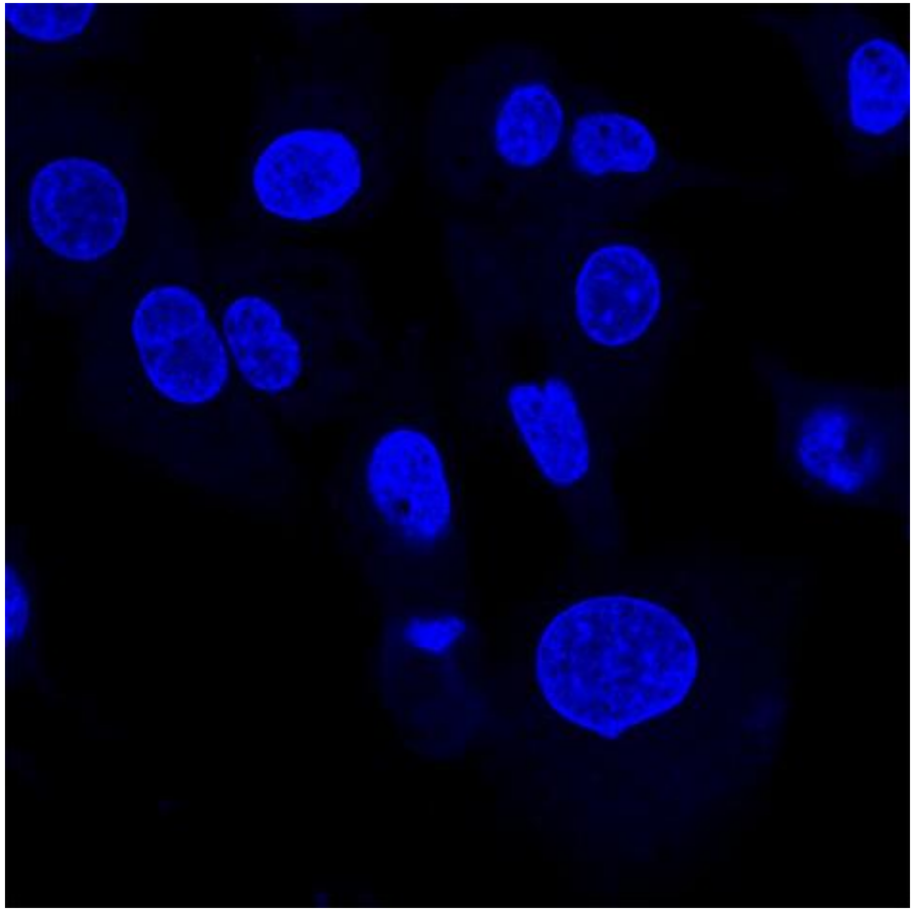

h

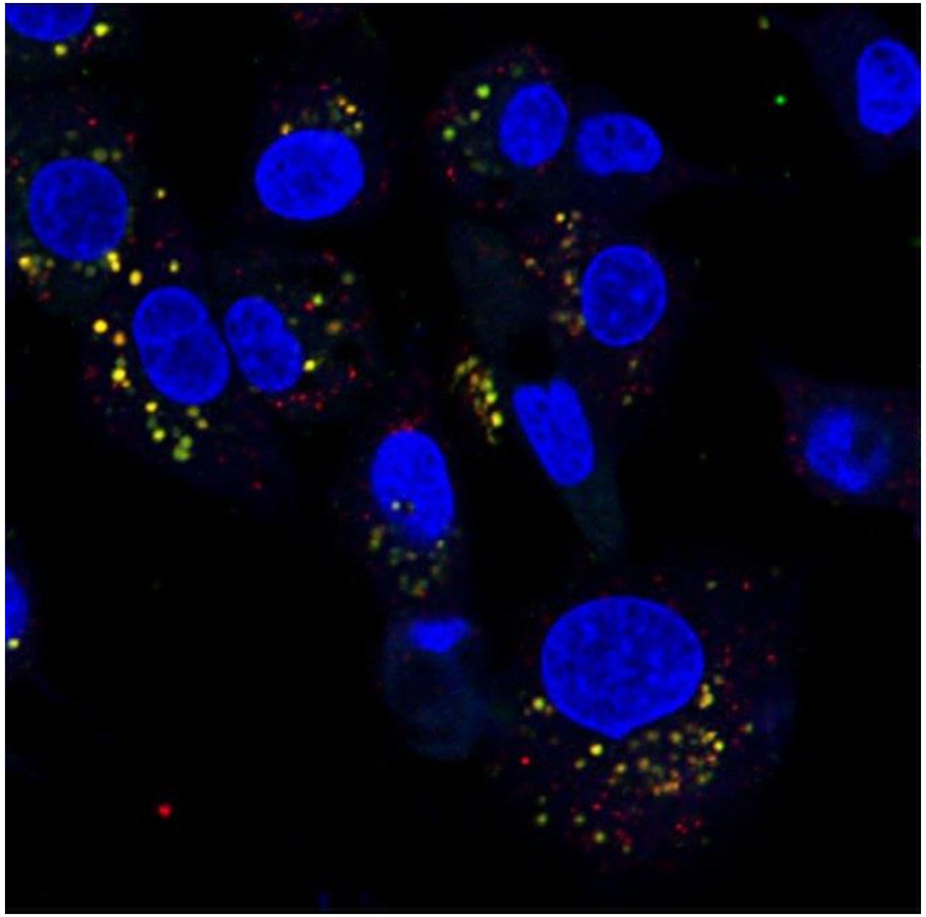

Supplement: S2 Data — (PDF) [file ppat.1011382.s010.pdf]
