# Supplementary material for: Hepatitis B virus hijacks TSG101 to facilitate egress via multiple vesicle bodies
Source: PLoS Pathog. 2023 May 24;19(5):e1011382. doi: 10.1371/journal.ppat.1011382 (PMC10208485; doi:10.1371/journal.ppat.1011382)
Supplement: Attachment — Submitted filename: Response-2023-04-04.docx [file ppat.1011382.s011.docx]

Dear editors,

We thank the reviewers and editors for their helpful comments, and are pleased that the reviewers regarded our work as “well-conceived” and “well organized and clearly stated”. We provided additional data regarding quantification of secreted virions, effects of certain mutations in HBc in supporting viral DNA synthesis, roles of 129PPAY132 motif in HBc-NEDD4 interaction and HBc ubiquitination, confirmation of the lysine residues catalyzed by NEDD4, focused ion beam scanning electron microscope (FIB-SEM) assay for MVBs in HepAD38 with TSG101 knockdown, and revised our manuscript based on constructive suggestions from the reviewers. Please find out point-by-point response below in *italic blue*.

**Part I - Summary**
Please use this section to discuss strengths/weaknesses of study, novelty/significance, general execution and scholarship.

Reviewer #1: Yingcheng Zhang, et al. report in this manuscript the essential role of TSG101 interaction with core protein in HBV virion morphogenesis. Specifically, in agreement with the prior work demonstrating the essential role of Cp K96 (but not K7) in HBV virion production (PMID: 35867543) and later domain activity of Cp 129PPAY132 motif (PMID: 24009707) as well as the critical role of ESCRT complexes in HBV virion budding, this report presents evidence showing that HBV virion production depends on TSG101 interaction with Cp in CpK96-, NEDD4- and later domain motif (Cp 129PPAY132)-dependent manner. Importantly, the work demonstrated, for the first time, that Cp is ubiquitylated. Overall, the study is well conceived. The results support the notion that HBV virion production depends on the later domain-mediated Cp ubiquitylation by NEDD4 and TSG101 interaction with ubiquitylated Cp. I have the following specific points to help further improving the manuscript.

Reviewer #2: This study assessed the mechanism on how HBV virions were sorted into MVBs for egress, and indicated that TSG101 recognition for NEDD4 ubiquitylated HBc is critical for MVBs-mediated HBV egress. The manuscript is well-organized and clearly stated. Some concerns need to be addressed.

**Part II – Major Issues: Key Experiments Required for Acceptance**
Please use this section to detail the key new experiments or modifications of existing experiments that should be absolutely required to validate study conclusions.

Generally, there should be no more than 3 such required experiments or major modifications for a "Major Revision" recommendation. If more than 3 experiments are necessary to validate the study conclusions, then you are encouraged to recommend "Reject".

Reviewer #1: 1. It is well known that naked capsids can be secreted (released) into culture media and interfere the accurate quantification of secreted virions by qPCR assays. Because the method for quantification of HBV DNA (virions) in culture media is not provided, it is not clear how this technical issue was addressed in this study.

*This is indeed a very important issue. To clarify viral particle types affected by TSG101, we performed CsCl intensity gradient centrifugation of HepAD38 cells culture supernatant concentration. As shown in Fig 1I, the fractions of HepAD38 cell supernatant represent HBV virion and naked capsid showed decreased HBV DNA levels after TSG101 knockdown. The results indicated that the majority of secreted viral DNA are encapsidated in Dane particles (3.67E+09 /* *4.76E+09 = 77.24%), which confirmed previously published data (PMID: 27245431).*

*To further evaluate the effects of TSG101 knockdown on virion secretion, we determined levels of HBV DNA after HBsAg antibody (NOVUS Biologicals) immunoprecipitation of culture media from HBV infected HepG2-NTCP cells. The data showed that TSG101 knockdown reduced HBV DNA levels of HBsAg antibody immunoprecipitated viral particles in culture medium. Based on these observations, we used supernatant HBV DNA as indicator of secreted HBV virion. The data is showed in new Fig 1E.*

*TSG101 expression was knocked down in HepG2-NTCP cells with HBV infection. The cells and culture medium were harvested 5 days post infection. Levels of HBV DNA in HBsAg antibody immunoprecipitation of culture medium were determined by absolute quantification qPCR.*

2. For the results presented in Fig. 4B-D and Fig. 4F-G, intracellular HBV core DNA should be detected to rule out the possibility that the reduced virion production is due to the deficiency of mutant Cp to support pgRNA packaging and/or viral DNA synthesis.

*Thanks for the suggestion. We co-transfected 1.2 HBV ΔHBc with HBc_WT_, HBc_K7R_, HBc_K96R_ or HBc_7/96R_ into Huh7 cells and determined the levels of HBc-associated HBV DNA. There was no significant difference among the mutants (A). We also investigated the effects of PP129AA and PPAY129AAAA mutants on pgRNA packaging and/or viral DNA synthesis. As expected, HBc_PPAY129AAAA_ failed to produce any HBc-associated HBV DNA, due to its capsid formation disability. However, the PP129AA mutation behaved similar to WT in intracellular HBV core DNA production (B). These results suggested that the mutants of HBc did not affect pgRNA packaging and viral DNA synthesis. This new data is included into new Fig 4E and I.*

***Effects of the HBc mutations on the levels of HBc-associated HBV DNA.*** *(A) 1.2 HBV ΔHBc was co-transfected with HBc_WT_, HBc_K7R_, HBc_K96R_, or HBc_K7/96R_. (B) 1.2 HBV ΔHBc was co-transfected with HBc_WT_, HBc_PP129AA_, or HBc_K7/96R_. Levels of HBc-associated HBV DNA were determined by qPCR.*

3. For results presented in Fig. 6A, it should be tested whether NEDD4 interaction with HBc is 129PPAY132 motif-dependent.

*As suggested by the reviewer, we transfected Myc-HBc_WT_ or Myc-HBc_PP129AA_ along with HA-NEDD4 into Huh7 cells. Co-IP assay revealed that PP129AA failed to interact with NEDD4. This data suggested that NEDD4 interaction with HBc was PPAY motif-dependent. The data is presented in new Fig. 6A.*

***PPAY motif is required for NEDD4 and HBc interaction.*** *Myc-HBc_WT_ or Myc-HBc_PP129AA_ was co-transfected with HA-NEDD4 into Huh7 cells. The interaction between HBc and NEDD4 was detected by Co-IP assay.*

4. It is not clear if the polyubiquitylation of HBc depends on the 129PPAY132 motif. You can also use your K7R and K96R mutant HBc to determine which lysine residue is ubiquitylated.

*Thank you for the suggestion. To confirm if the polyubiquitylation of HBc depends on the 129PPAY132 motif, we immunoprecipitated HBc_WT_ and HBc_PP129AA_ from HA-ubiquitin co-transfected Huh7 with NEDD4 knockdown, and detected the levels of ubiquitination by WB. The results showed that 129PPAY132 mutant demonstrated strongly suppressed ubiquitination level in the presence of NEDD4, and knockdown of NEDD4 only reduced the levels of wild type HBc ubiquitination but not PP129AA mutant (A), suggesting that PPAY motif was critical for HBc ubiquitination. To further dissect which lysine residue was catalyzed by NEDD4, we investigated ubiquitination levels of HBc_K7R_, HBc_K96R_, and HBc_K7/96R_. Interestingly, while both K7R and K96R mutants showed mitigated ubiquitination levels, K7/96R presented dramatic loss of ubiquitination signal (B). NEDD4 knockdown further inhibited the ubiquitination levels of wild type HBc, HBc_K7R_, and HBc_K96R_, suggesting that both K7 and K96 residue ubiquitination were catalyzed by NEDD4. The new data is presented in new Fig 6C and G.*

***PPAY motif is critical for NEDD4 catalyzed HBc ubiquitination. NEDD4 catalyzes K7 and K96 ubiquitination.*** *(A) HBc_PP129AA_ was co-transfected with HA-ubiquitin into the Huh7 cells with NEDD4 knockdown. (B) HBc_K7R_, HBc_k96R_, or HBcK_7/96R_ was co-transfected with HA-ubiquitin into the Huh7 cells with NEDD4 knockdown. Ubiquitination levels of immunoprecipitated HBc were determined by WB.*

5. It is also interesting to know if the HBc ubiquitylation occurs in the context of nucleocapsids, empty capsids and/or free dimers. In another word, is the morphogenesis of complete virions and incomplete virions via the same or distinct mechanism?

*This is indeed a very interesting question. However, the aim of our present study is to dissect the mechanism of HBV virion egress.* *The morphogenesis of incomplete virions is out of our scope.*

*Nevertheless, we showed in Fig 1I that secreted levels of naked capsid reduced when TSG101 was knockdown. To further evaluate the effect of TSG101 knockdown on nucleocapsids secretion, we immunoprecipitated capsids in TSG101 knockdown HepAD38 cell culture medium and determined the levels of HBc and HBV DNA after capsid disruption. The data below showed that levels of HBc and HBV DNA in immunoprecipitation were decreased by TSG101 knockdown indicating that TSG101 knockdown restrained nucleocapsid release. Detailed mechanisms of incomplete virions morphogenesis, including empty capsids and free dimers, will be conducted in a separate study.*

***Nucleocapsids secretion was restrained by TSG101 knockdown.*** *Capsids in TSG101 knockdown HepAD38 cell culture supernatants were immunoprecipitated. Levels of HBc were determined by WB. Levels of HBV DNA were determined after capsid disruption.*

Reviewer #2: 1. In most figures (for example Fig 1B), the knockdown efficiency of siRNA should be determined by protein level using Western blot.

*It is indeed important to confirm knockdown efficiency of every siRNA as the reviewer pointed out. For our initial siRNA screening of 6 genes, we only wanted to select one factor which showed strong effect on HBV replication. Due to the availabilities, uncertain performance, and cost to obtain all antibodies, we used qPCR to evaluate the siRNAs knockdown efficiency. Further investigation led us to focus on TSG101. We performed WB to confirm knockdown efficiency when we confirmed the phenomenon of TSG101, and later NEDD4 (Figure 1G and K; Figure 5C and E; Figure S2B and S2F; Figure S5A and S5D).*

2. Line 184: TSG101 ubiquitination at these two sites should be performed.

*The reviewer raised a very important question. In this revision, we conducted additional experiments to study the role of K7 and K96 in HBc ubiquitylation. Please see our response to reviewer 1 question 4 for the details.*

3. Fig 2B and 2D: Is there any basis for the morphological identification of MVBs in transmission electron microscopy experiments.

*Thanks for pointing this issue out. Late endosomes/MVBs were a single layer membrane with a patch of high electron density. To further confirm the morphological identification of MVBs, we performed focused ion beam scanning electron microscope (FIB-SEM). As showed in new Fig 2E, TSG101 knockdown reduced the size of MVBs in HepAD38 cells. We also provided the scanning of whole cells as supplementary videos (Video 1 and 2).*

*Representative images of FIB-SEM assay for MVBs (red arrow) in HepAD38 cells with TSG101 knockdown.*

**Part III – Minor Issues: Editorial and Data Presentation Modifications**
Please use this section for editorial suggestions as well as relatively minor modifications of existing data that would enhance clarity.

Reviewer #1: 1. Line 37, the “ESCRT” should be spelled out.

*We have spelled out “ESCRT”as “The Endosomal Sorting Complex Required for Transport” in the revised manuscript.*

2. The sentence starting at line 70 “However, it is still unclear which host factor(s) recognizes HBV virions and how is the sorting into MVBs orchestrated for HBV to egress the cell.” is not a clear statement. My understanding is that MVBs are the place for HBV virion morphogenesis and sorting/secretion. Accordingly, a better statement is: “it is still unclear which and how host factors recognize HBV capsids and facilitate the morphogenesis and egress of HBV virions from hepatocytes”.

*We have replaced the sentence as suggested.*

3. Although the result presented in Fig. S1A showed that flagged-tagged HBc assembles into capsids. Do you know the Cp pulled down in your IP-mass spectrometry analysis is in the form of capsids or not?

*To clarify this point, we performed immunoprecipitation of the Cp by Myc antibody coated agarose beads and eluted with 1M glycine (pH = 2). However, we encountered technical problems as the elution buffer itself resulted in capsids disruption (probably caused by high concentrations of H^+^).*

4. The rationale for the selection of the six proteins for further investigation is not clearly described.

*GO analysis of biological process and cellular component for the identified HBc-associated factors showed that they were associated with “protein transport”, “viral process” and located in “cytoplasm” and “extracellular exosome”. This suggested that the HBc binder might regulate virus egress. So, we selected the common factors under the terms “protein transport”, “viral process”, and “extracellular exosome”. We have updated relative statement in revised manuscript.*

5. Fig. 1H, it appears that knockdown of TSG101 also reduced the production of SVPs. The role of TSG101 in SVP secretion should be more thoroughly discussed.

*TSG101 is a component of ESCRT regulating MVB biogenesis (PMID: 16707569). TSG101 is also a cargo sorting substrate into exosome and is commonly used as exosome marker (PMID: 12802020). Exosome are derived within MVBs (PMID: 3597417). It has been reported that a fraction of HBV virions and subviral particles can be released as exosomes (PMID: 34278172; PMID: 36184032). So, it is reasonable that subviral particles secretion were also restrained when TSG101 expression was inhibited. We have added the discussion in the revised manuscript.*

6. In Fig S3A, in DOX (-) and DOX (+), at least core or other viral markers should be examined to confirm the experimental condition.

*We have performed WB to determine the levels of HBc. The treatment of 1 μg/ml DOX for 10 days significantly reduced the level of HBc in HepAD38 cells. The data is included in new Fig 2A.*

*HepAD38 cells were treated with 1 μg/ml DOX for 10 days. Levels of HBc were determined by WB.*

7. In the legend of Fig. 7, is there published evidence for tenatoprazole inhibition of HBV virion production? If yes, it should be cited and discussed in the text.

*We have deleted the sentence “Tenatoprazole could bind TSG101 and restrict HBV egress” from the legend of Figure 7.*

8. In the sentence from line 285 - 287, the “HBV particles” indicate HBV virions or capsids? The amounts of virions or virion-like particles are reduced in TSG101 knockdown cells (Fig. 2E).

*We have revised “HBV particles” in line 285 - 287 as “virions or virion-like particles”.*

9. Sentence from line 333 - 334, no direct evidence to support this claim.

*We have revised the sentence as “so we speculated that TSG101 might influence exosome mediated HBV virion and subviral particle release”.*

Reviewer #2: 1. In most figures (for example Fig 1B), the knockdown efficiency of siRNA should be determined by protein level using Western blot.

*It is indeed important to confirm knockdown efficiency of every siRNA as the reviewer pointed out. For our initial siRNA screening of 6 genes, we only wanted to select one factor which showed strong effect on HBV replication. Due to the availabilities, uncertain performance, and cost to obtain all antibodies, we used qPCR to evaluate the siRNAs knockdown efficiency. Further investigation led us to focus on TSG101. We performed WB to confirm knockdown efficiency when we confirmed the phenomenon of TSG101, and later NEDD4 (Figure 1G and K; Figure 5C and E; Figure S2B and S2F; Figure S5A and S5D).*

2. Line 184: TSG101 ubiquitination at these two sites should be performed.

*The reviewer raised a very important question. In this revision, we conducted additional experiments to study the role of K7 and K96 in HBc ubiquitylation. Please see our response to reviewer 1 question 4 for the details.*

3. Fig 2B and 2D: Is there any basis for the morphological identification of MVBs in transmission electron microscopy experiments.

*Thanks for pointing this issue out. Late endosomes/MVBs were a single layer membrane with a patch of high electron density. To further confirm the morphological identification of MVBs, we performed focused ion beam scanning electron microscope (FIB-SEM). As showed in new Fig 2E, TSG101 knockdown reduced the size of MVBs in HepAD38 cells. We also provided the scanning of whole cells as supplementary videos (Video 1 and 2).*

*Representative images of FIB-SEM assay for MVBs (red arrow) in HepAD38 cells with TSG101 knockdown.*
